# Exploring the Natural Compounds in Flavonoids for Their Potential Inhibition of Cancer Therapeutic Target MEK1 Using Computational Methods

**DOI:** 10.3390/ph15020195

**Published:** 2022-02-03

**Authors:** Wejdan M. AlZahrani, Shareefa A. AlGhamdi, Torki A. Zughaibi, Mohd Rehan

**Affiliations:** 1 Department of Biochemistry, Faculty of Sciences, King Abdulaziz University, Jeddah 21589, Saudi Arabia; wmuhammadalzahrani@stu.kau.edu.sa; 2 King Fahd Medical Research Center, King Abdulaziz University, Jeddah 22252, Saudi Arabia; taalzughaibi@kau.edu.sa; 3 Department of Medical Laboratory Sciences, Faculty of Applied Medical Sciences, King Abdulaziz University, Jeddah 21589, Saudi Arabia

**Keywords:** MEK1, flavonoids, virtual screening, molecular docking, ADMET, molecular dynamic (MD) simulation

## Abstract

The Mitogen-Activated Protein Kinase (MAPK) signaling pathway plays an important role in cancer cell proliferation and survival. MAPKs’ protein kinases MEK1/2 serve as important targets in drug designing against cancer. The natural compounds’ flavonoids are known for their anticancer activity. This study aims to explore flavonoids for their inhibition ability, targeting MEK1 using virtual screening, molecular docking, ADMET prediction, and molecular dynamics (MD) simulations. Flavonoids (*n* = 1289) were virtually screened using molecular docking and have revealed possible inhibitors of MEK1. The top five scoring flavonoids based on binding affinity (highest score for MEK1 is −10.8 kcal/mol) have been selected for further protein–ligand interaction analysis. Lipinski’s rule (drug-likeness) and absorption, distribution, metabolism, excretion, and toxicity predictions were followed to find a good balance of potency. The selected flavonoids of MEK1 have been refined with 30 (ns) molecular dynamics (MD) simulation. The five selected flavonoids are strongly suggested to be promising potent inhibitors for drug development as anticancer therapeutics of the therapeutic target MEK1.

## 1. Introduction

Cancer is a leading cause of death in nearly every country in the world, as well as being the most significant barrier to extending life expectancy in the twenty-first century [1]. More than nearly 20 million new cases are predicted to be registered by 2025. Various implications are considered as hallmarks of cancer cells, including proliferative signaling, growth suppressor escape, cell death resistance, immortality, and angiogenesis induced by invasion–metastasis [2]. Since cancer is generally associated with several mutations that affect the main signaling pathways [3], targeted cancer therapy takes advantage of tumor-specific genetic vulnerabilities and mutations and designs therapeutics directly targeting cancer cells. The important therapeutic targets (mostly enzymes) are those whose suppression eventually kills cancer cells and that have minimal effect on normal tissues as they are either mutated or over-expressed in cancer cells [4,5]. One of the pathways that is commonly altered and activated, having a role in oncogenesis, the progression of the tumor, and drug resistance in cancer, is the Mitogen-Activated Protein Kinases (MAPK) signaling pathway [3,6]. The MAPK cascade, which is also known as RAS-regulated RAF-MEK1/2-ERK1/2 or ERK signaling pathway [7], is comprised of a number of kinases that carry out specific cell fate decisions in response to the input signal, explaining why numerous targeted therapies have been targeting this pathway [3,8]. By activating the epidermal growth factor receptor (EGFR) and the RAS small guanosine triphosphatases (GTPases) upstream, MAPK signaling promotes cell proliferation, survival, and metastasis. Mitogen-Activated Protein Kinase/Extracellular Regulated Kinase/Kinase 1 and 2 (MEK1/2) dual-specificity protein kinases are phosphorylated and activated by RAF kinases. MEK1/2 then phosphorylate and activate the Extracellular Regulated Kinase 1 and 2 (ERK1/2). Activated ERKs phosphorylate and regulate the activities of over 160 proteins that are estimated to be involved [9].

Protein kinases and phosphatases control the phosphorylation of proteins which makes them control nearly every aspect of the cell, therefore making them ideal candidates for evolutionary studies [10,11]. MEK1 and MEK2 are dual-specificity protein kinases; both are tyrosine (Y-) and serine/threonine (S/T-) protein kinases. A particular aspect of these protein kinases is that they are the core component of kinases for the MAPK/ERK signaling cascade as well as the gatekeepers of ERK1/2 activity, which is especially exciting since downstream ERK has multiple targets and transcription factors that control the critical processes in the cell. Thus, therapeutic targeting of MEK1/2 is relatively specific [12,13,14]. MEK1 and MEK2 are nearly identical, with a distinct pocket structure which is proximate to the ATP-binding site despite being distinct at the same time. Several conformational changes occur when an inhibitor binds to this region, locking unphosphorylated MEK1/2 into a catalytically inactive state. This occurs through the highly conserved DFG-out motif (Asp, Phe, and Gly) in the activation loop which exposes a site adjacent to the ATP binding site where inhibitors could bind to and lock the protein in an inactive state. Furthermore, since this ATP-noncompetitive process does not inhibit other protein kinases via the highly conserved ATP-binding pocket, unwanted side effects, such as inadvertent inhibition of other protein kinases and the challenge of competing with millimolar intracellular ATP concentrations, are largely avoided [15,16,17]. Unfortunately, most ATP-competitive kinase inhibitors interact with numerous members of the protein kinase family, making it challenging to build selective inhibitors for a single kinase target. Moreover, as a result of changes in the kinase domain, cancer cells can develop acquired drug resistance, making specific kinase inhibitors less effective. For these reasons, finding ATP-noncompetitive kinase inhibitors is becoming more desirable as a therapeutic development method [18]. Various popular pharmaceutical companies have shown great interest in MEK1 since it’s highly selective of the MAPK/ERK pathway [19], and several MEK inhibitors are currently under clinical development [20].

Natural products can help treat a range of human disorders, including the world’s second largest cause of death: cancer [21]. Lately, the interest in the natural products to be used as anticancer agents has increased, and phytochemicals have become valuable in anticancer drug development. More than 75% of the approved anticancer drugs between 1981 and 2007 are either natural products or have been developed based on them [22,23]. Flavonoids are among the proposed natural products for cancer prevention that have been increasingly found to have a major health impact [24]. Flavonoids are polyphenolic compounds obtained as small plant derivatives’ secondary metabolites [25]. Since the discovery of the first flavonoid (rutin) in 1930, more than 6500 different flavonoids have been reported in various plant species, and their actual number is estimated to exceed 8000 [26,27]. A wide variety of biological and pharmacological effects have been found in flavonoids, such as their numerous antioxidant, antiproliferative, anti-inflammatory, antihypertensive, anti-carcinogenic, etc. effects, but the most notable activity is their potential role as anticancer agents [22,25,26,28,29,30,31]. Regarding the exciting feature of flavonoids is that they are by far the largest group of natural compounds that inhibit protein kinases and fit well into the ATP binding pocket and the neighboring area [32]. Others reported flavonoids’ inhibition activity against Ser/The protein kinases 18 and receptor tyrosine kinases (RTKs) [33,34]. Various in silico, in vitro, and in vivo studies demonstrate the anticancer activities of flavonoids in several types of cancer [35,36,37,38,39,40,41,42,43,44]. Few studies have shown their anticancer relationship with the ERK and MAPK cascade in various cancer types [45,46,47,48,49,50,51]. Myricetin is one of the flavonoids which has been found to inhibit MEK1 in vivo and in vitro, in addition to being a potent ATP-noncompetitive inhibitor of MEK1 [52,53]. Another flavonoid is nobiletin, which has shown an in vitro antitumor effect against MEK in human fibrosarcoma HT−1080 cells [54]. Isorhamnetin is a flavonoid that inhibits MEK1 in the ATP-noncompetitive binding site [55]. A flavonoid that has shown potent and specific ATP-noncompetitive inhibition activity of MEK1 is PD 098059, which is the first MEK inhibitor to be discovered [56] and mostly used in laboratory experiments.

The current work involved the use of computational methods for the design of an MEK1 inhibitor. The computational methods, including molecular docking and binding simulation analysis, have increasingly been used for binding pose and designing of novel inhibitors or better derivatives of known existing inhibitors [57,58,59,60,61,62,63,64,65,66,67,68]. 

The current study proposed five novel MEK1 inhibitors and anticancer drug candidates by virtual screening of the natural compound flavonoids. A library of all known flavonoids was prepared and the top five screened compounds were proposed as potential MEK1 inhibitors and anticancer agents. The proposed compounds were further checked for key interacting residues, molecular interactions, binding energy, and dissociation constant using various methods. Finally, to show the stability of the protein–ligand, complexes were subjected to MD simulation analysis. This study will provide novel MEK1 inhibitors and anticancer agents with their action mechanism. The proposed flavonoids can further be tested experimentally for their potential use as novel anticancer agents.

## 2. Results and Discussion

### 2.1. Virtual Screening of Natural Compound Class Flavonoids for MEK1 Inhibition

**Figure 1:**
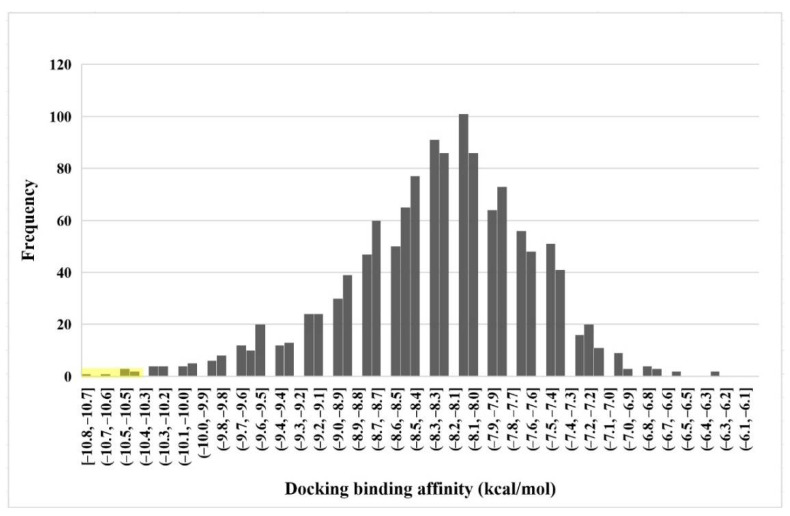
Histogram plot of dock binding affinity of all flavonoids. The dock scores of the selected flavonoids in the left part are highlighted in yellow.

**Figure 2:**
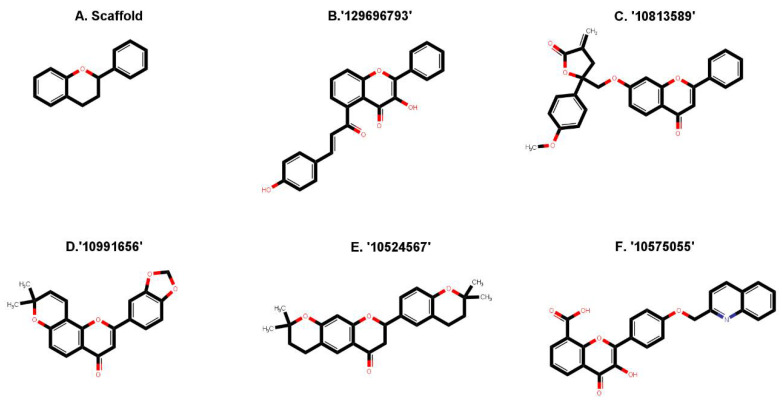
Two-dimensional sketch of flavonoid scaffold (**A**) and top selected flavonoids for MEK1 (**B**–**F**). The flavonoids are represented by their PubChem CID. Heteroatoms oxygen (O), and nitrogen (*N*)with their balancing hydrogens are shown as red, blue, and green, respectively.

The capacity of molecular docking studies to anticipate the right binding conformations of small molecules as ligands to the appropriate target binding site is an important feature of in silico drug discovery. In this view, the goal of our study was to do virtual screening of flavonoids for possible MEK1 inhibition, which is an essential protein target in the MAPK pathway in cancer cells. (Figure 1) shows the histogram of dock scores for the screened compounds (Supplementary File S1) where the selected top five ranked compounds are highlighted in the left. Most of the compounds are providing docking affinity (>−7.0 kcal/mol), but the highest scoring and best fit compounds were selected and the redundant compounds were omitted to give the best and most diverse structure compounds. The top five selected flavonoids (Figure 2) against MEK1 in (Table 1) show the highest docking score of (−10.8 kcal/mol) and the fifth rank with a score of (−10.4 kcal/mol) compared to the docking score of native inhibitor (−9.0 kcal/mol). The native inhibitor of the 3D structure is PD318008 (5-bromo-*N*-(2,3-dihydroxypropoxy)-3,4-dofluoro−2-[(2-fluoro-4-iodophenyl)amino]benzamide), an analog of PD184352 which is a highly selective, ATP-noncompetitive, and potent MEK1 and MEK2 inhibitor [16]. These results suggest that the selected flavonoids are probably better inhibitors of MEK1. Further, the molecular docking of trametinib, a specific and highly selective inhibitor of MEK1/2, was performed and gave the score of (−9.7 kcal/mol), which is also lower than those of the selected flavonoids, providing credence to the selected compounds [69].

**Figure 3:**
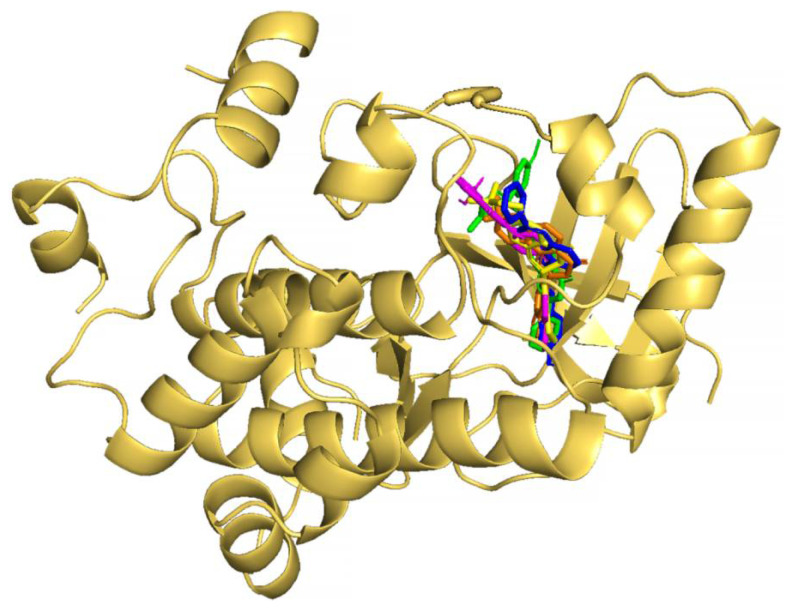
Molecular docking of top 5 selected flavonoids to MEK1. The protein is shown in cartoon representation colored yellow-orange, while compounds are shown in stick representations in various colors: binding of flavonoids ‘129696793’ (blue), ‘10813589’ (green), ‘10991656’ (orange), ‘10524567’ (yellow), and ‘10575055’ (magenta).

**Figure 4:**
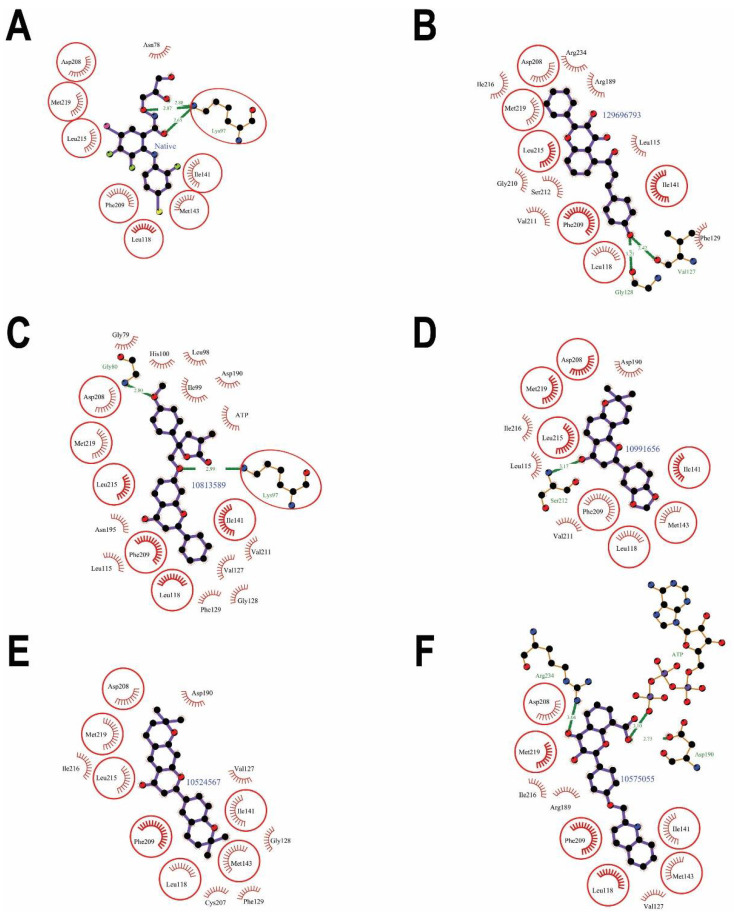
MEK1 protein–ligand interaction plots of native inhibitor (**A**) and selected flavonoids (**B**–**F**). The residues forming non-bonding interactions are shown as red bristles, while residues forming hydrogen bond and the bound ligand are shown as ball-and-stick representations. The carbon atoms are shown as black balls, nitrogen atoms as blue balls, oxygen atoms as red balls, fluorine atoms as green balls, bromine atom as pink balls, and iodine atom as a yellow ball. The interacting residues common with those of the native inhibitor are shown in circles. The hydrogen bonds are shown as green dashed lines labeled with bond length (in Å).

The molecular docking results of the first rank flavonoid (CID: 129696793) against the MEK1 binding pocket showed that the pocket fit well in the catalytic site (Figure 3) and interacted with 16 amino acids: Leu-115, Leu-118, Val-127, Gly-128, Phe-129, Ile-141, Arg-189, Asp-208, Phe-209, Gly-210, Val-211, Ser-212, Leu-215, Ile-216, Met-219, and Arg-234 (Figure 4B). The binding strength scores’ docking affinity (−10.8 Kcal/mol), binding energy (−10.25 Kcal/mol), and dissociation constant ($pK_{d}$, 7.52) showed quality binding as required for adequate inhibition (Table 1). The molecular interaction (Table 2) showed 2 hydrogen bonds and 62 (16) non-bonded contacts (hydrophobic interactions). The key interacting residues were Leu-118, Ile-141, Asp-208, Phe-209, and Ile-216 with 5, 6, 5, 9, and 9 non-bond contacts, respectively. The Met-219 turned out to have the maximum ΔASA (loss in Accessible Surface Area) (42.37 Å2) followed by Asp-208 (39.18 Å2). Hydrogen bonds were formed by Val-127 and Gly-128 measure 2.42 Å and 3.21 Å, respectively. Observing amino acid residues from the flavonoid binding site within the target protein seeks to predict the interactions that occur and that are thought to contribute to the flavonoid compound’s pharmacological activities, such as MEK kinase inhibition. Inhibition of enzymatic activity of a protein by a compound is largely due to non-covalent bonds, including non-bonded contacts and hydrogen bonds [70]. The binding of native inhibitor with interacting residues and their interactions is also provided for comparison (Figure 4A). Comparing to the binding of the native inhibitor, there are six residues, Leu-118, Ile-141, Asp-208, Phe-209, Leu-215, and Met-219, common to the lists of interacting residues of this compound and the native inhibitor, and they include the key residues Asp-208 and Phe-209. The fact that the proposed compound binds to the similar group of residues in the catalytic site validates our prediction that it is comparable to the native inhibitor.

The second highest ranked flavonoid (CID: 10813589) against the MEK1 binding pocket docked well (Figure 3) and interacted with ATP and 19 amino acids residues: Gly-79, Gly-80, Lys-97, Leu-98, Ile-99, His-100, Leu-115, Leu-118, Val-127, Gly-128, Phe-129, Ile-141, Asp-190, Asn-195, Asp-208, Phe-209, Val-211, Leu-215, and Met-219 (Figure 4C). The strength of binding scores’ docking affinity of (−10.6 Kcal/mol), binding energy (−10.96 Kcal/mol) and dissociation constant ($pK_{d}$, 8.03) were reasonably high, as required for good inhibition (Table 1). The protein–ligand complex was stabilized by non-bonding interactions (Table 3) through 78 (20) non-bonded contacts and 2 hydrogen bonds. The key interacting residues were Ile-99, Asp-208, Lys-97, and Phe-209 with 15, 9, 8, and 6 non-bonding interactions of each, respectively. Met-219 turned out to have the maximum ΔASA with (43.97 Å^2^), followed by Asp-208 with (39.24 Å^2^) and Lys-97 with (31.09 Å^2^). Hydrogen bonds in Gly-80 and Lys-97 measured 2.80 Å and 2.99 Å, respectively. Of the 19 interacting residues, 7 residues were common with those of the binding of the native inhibitor: Lys-97, Leu-118, Ile-141, Asp-208, Phe-209, Leu-215, and Met-219 (Figure 4A), including the key residues Lys-97, Asp-208, and Phe-209. The proposed compound bound to the same set of residues in the catalytic site, making it promising as the native inhibitor.

The third rank flavonoid (CID: 10991656) bound to the MEK1 binding pocket (Figure 3) and showed interactions with 12 residues: Leu-115, Leu-118, Ile-141, Met-143, Asp-190, Asp-208, Phe-209, Val-211, Ser-212, Leu-215 Ile-216, and Met-219 (Figure 4D). The predicted binding strength scores for the protein–ligand complex were docking affinity of (−10.5 Kcal/mol), binding energy (−9.49 Kcal/mol), and dissociation constant ($pK_{d}$, 6.95) and showed quality binding as required for good inhibition (Table 1). The molecular interaction showed one hydrogen bond through Ser-212 measures 3.17 Å and 54 (12) non-bonded contacts (hydrophobic interactions). The key interacting residues (Table 4) are Asp-208 and Phe-209, with 13 and 10 non-bonding interactions of each, respectively. The maximum ΔASA is with Met-219 residue (43.97 Å^2^) followed by Asp-208 with (40.1 Å^2^). The interacting residues and their interactions with the native inhibitor are also provided for comparison (Figure 4A). Out of the 12 interacting residues, 7 residues were common with those of the native inhibitor: Leu-118, Ile-141, Met-143, Asp-208, Phe-209, Leu-215, and Met-219, which included the key residues Asp-208 and Phe-209. This also suggested that the proposed compound was blocking the same set of residues as the native inhibitor, thereby inhibiting the protein’s action.

The dock results of the fourth ranked flavonoid (CID: 10524567) bound in the MEK1 binding pocket (Figure 3) showed interactions with 61 non-bonded contacts (hydrophobic interactions) with 13 amino acids: Leu-118, Val-127, Gly-128, Phy-129, Ile-141, Met-143, Asp-190, Cys-207, Asp-208, Phe-209, Leu-215 Ile-216, and Met-219 (Figure 4E). The binding strength scores’ docking affinity (−10.5 Kcal/mol), binding energy (−10.83 Kcal/mol), and dissociation constant ($pK_{d}$, 7.94) showed as quality binding, as required for good inhibition (Table 1). The key interacting residues are Asp-208 and Phe-209 with 11 and 14 non-bonding interactions of each, respectively. Met-219 turned out to have the maximum ΔASA with (52.28 Å^2^), then Asp-208 with (38.96 Å^2^) and next Asp-190 with (30.23 Å^2^) (Table 5). Compared to the native inhibitor (Figure 4A), there are seven common amino acids: Leu-118, Ile-141, Met-143, Asp-208, Phe-209, Leu-215, and Met-219, and they share the same key residue of Asp-208 and Phe-209 and thus, they may inhibit MEK1 kinase activity in the same way as the native inhibitor does. 

This compound is a chiral compound and possesses R and S stereoisomeric configurations, as shown in Supplementary Figure S1. The above docking results of this compound are in R-stereoisomer configuration. In order to check the effect of S-stereoisomer of this compound on the binding to MEK1, the molecular docking was performed. The molecular docking of the (S) configuration of the chiral compound also showing high affinity, with (−10.9 Kcal/mol), binding energy (−10.85 Kcal/mol), and dissociation constant ($pK_{d}$, 7.96). The interaction showed 1 hydrogen bond and 58 non-bonded contacts with ATP and 16 amino acids: Lys-97, Ile-99, Leu-115, Leu-118, Val-127, Ile-141, Met-143, Asp-190, Leu-206, Cys-207, Asp-208, Phe-209, Gly-210, Val-211, Leu-215, and Met-219 (Table S2 in Supplementary file2). The hydrogen bond with Lys-97 measures 3.01 Å. The key interacting residues are: Phe-209 and Met-219 with 12 and 6 non-bonded contacts, respectively. Met-210 has the maximum with (48.6 Å^2^), then Asp-208 with (44.44 Å^2^). Comparing the interaction to the native inhibitor, the eight residues are common (Supplementary Figure S2): Lys-97, Leu-118, Ile-141, Met-143, Asp-208, Phe-209, Leu-215, and Met-219, and they share the same key residue, Phe-209. However, there is no literature upon the stereoisomerism of this compound. This can be a good chance to study the effect of stereoisomerism of this compound on the binding to the MEK1 kinase.

The dock results of the fifth ranked flavonoid (CID: 10575055) against the MEK1 binding pocket fit well within the catalytic site (Figure 3) and interacted with ATP and 11 amino acids residues, namely Leu-118, Val-127, Gly-128, Ile-141, Met-143, Arg-189, Asp-190, Asp-208, Phe-209, Met-219, and Arg-234 (Figure 4F). The results showed docking affinity of (−10.4 Kcal/mol), binding energy (−10.11 Kcal/mol), and dissociation constant ($pK_{d}$, 7.41) were also reasonably high as required for adequate MEK1 kinase inhibition (Table 1). The molecular interaction shows 3 hydrogen bonds and 44 (11) non-bonded contacts (hydrophobic interactions). The three hydrogen bonds with Asp-190, Arg-234, and ATP measure 2.73 Å, 3.04 Å, and 3.10 Å, respectively. The key interacting residues are Asp-208 and Phe-209, with 9 and 11 non-bonding interactions, respectively. Met-219 turned out to have the maximum ΔASA with (50.93 Å2), and then Asp-208 with (40.01 Å2) (Table 6). Of the 11 interacting residues, 6 residues were common among the interacting residues of the native inhibitor: Leu-118, Ile-141, Met-143, Asp-208, Phe-209, and Met-219 (Figure 4A), including the same key residues Asp-208 and Phe-209; thus, they might inhibit the MEK1 kinase activity similar to the native inhibitor.

### 2.2. Drug-Likeness and Pharmacokinetics Prediction

The strength of the ligand binding on the target protein is not the only factor in the discovery of novel medications. To assess the degree of effectiveness and therapeutic efficacy, it is also studied in terms of drug-likeness, pharmacokinetics, and toxicity. Lipinski’s rule of five predicts the drug-likeness of the selected compounds. Moreover, pharmacokinetics including Absorption, Distribution, Metabolism, Excretion, and Toxicity (ADMET) play an important role in medicinal chemistry, which describes how drugs move through the body. The prediction of drug-likeness for the selected inhibitor for MEK1 is presented in (Table 7). Considering the desired values: molecular weight < 500, H-bond donors < 5, H-bond acceptors < 10, Rotatable bonds < 10, and lipophilicity (logP) < 5, all the selected compounds follow the desired values, except for (CID: 10813589) and (CID: 10524567), where the lipophilicity is slightly higher than 5. All the ADMET predicted properties of the top selected inhibitors for MEK1 are presented in (Table 8). The efficacy of the selected compounds as oral medicine was determined using two models for measuring absorption properties, including CaCO2 permeability and intestinal absorption. Where the desired CaCO2 permeability is >0.90 and intestinal absorption >30%, the prediction of the screened compounds shows the CaCO2 permeability values all in positive integers, with exception of (CID: 10575055). While intestinal absorption shows a high percentage, all are higher than 70%, which is considered as good absorption. Skin permeability is the next important factor in absorption. The ideal skin permeability is >−2.5 log Kp and all of the compounds under study have permeability values of less than −2.5 log Kp, indicating poor skin permeability. The ATP-binding cassette (ABC) transporter, which is necessary for efficient molecular transport across cell membranes, contains P-glycoprotein. P-glycoprotein substrates, and inhibitors of P-glycoprotein I and II which were examined in all of the substances that were screened. Except for (CID: 10813589) and (CID: 10524567), all of the compounds were found to be substrates, indicating that they can be transported through the cell membrane through the ABC transporter. The (CID: 10575055) was found to be ineffective as an inhibitor of P-glycoprotein I transporter, suggesting that they could be incapable of inhibiting these drug efflux pumps. The distribution of the substances in the body was determined using four distinct assays: volume of distribution (VDss), fraction unbound, BBB permeability, and central nervous system (CNS) permeability. To begin with, in the VDss assay, which is used to evaluate the total amount of drugs needed for uniform drug distribution in the bloodstream, readings less than −0.15 log are considered negative, while values greater than 0.45 log are considered good diffusion. Thus, (CID: 10991656) and (CID: 10524567) show average VDss values, while other compounds have low distribution volume. The potential of a drug to reach the brain is determined by the permeability of the blood–brain barrier (BBB). If the logBB values are more than 0.3, they will cross BBB. The logBB value of the screened compounds are less than 0.3, meaning that none of them will be able to cross BBB except for (CID: 10991656). The desired value of the CNS permeability is >−2 and the screen shows good results except for (CID: 10575055), where it was less than the desired value. Seven distinct cytochrome models were used to examine the test drug’s metabolism in the body. All of the compounds were tested for their capacity to inhibit CYP1A2, CYP2C19, CYP2D6, CYP2C9, and CYP3A4 as well as their ability to function as a substrate for CYP2D6 and CYP3A4. The total clearance rates of all of the examined compounds were varied, and none of them appeared to be a substrate for organic cation transporter 2 (OCT2). They also failed to anticipate AMES toxicity, showing that these chemicals are neither carcinogenic nor mutagenic, except for: (CID: 129696793), and (CID: 10813589). Three of the selected flavonoids predicted to be negative for hepatotoxicity were (CID: 10813589), (CID: 10991656), and (CID: 10524567), whereas none of the substances tested positive for skin sensitization. Overall, the selected compounds proposed to be safe drug-candidates for human cancer therapy.

### 2.3. MD Simulation

**Figure 5:**
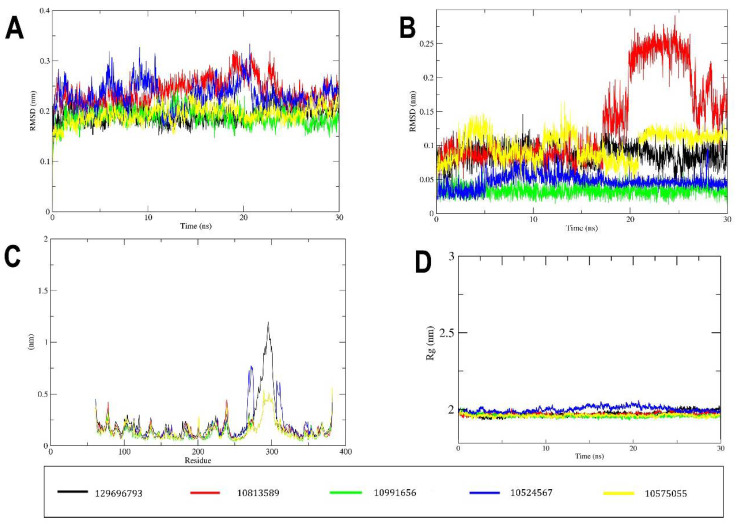
MD simulation results of the docked flavonoids obtained from virtual screening. (**A**) Root–mean–square deviation (RMSD) curve for the protein backbone of the protein–ligand complex. The RMSD plot provides quantification of the overall stability of the protein backbone during 30 ns simulation. (**B**) RMSD curve for ligands’ heavy atoms through the simulation. (**C**) Root–mean–square fluctuations’ (RMSF) curve of the MEK1 residues for the protein–ligand complexes. (**D**) Radius of gyration (total) of protein–ligand complex.

**Figure 6:**
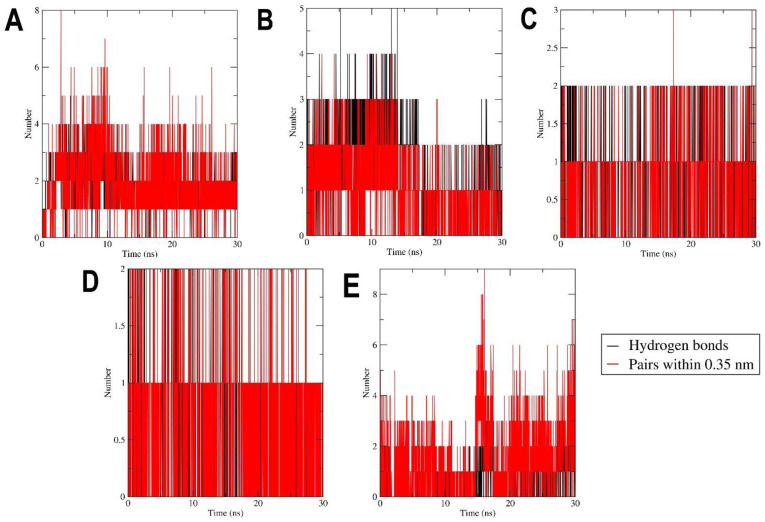
Time evolution of number of hydrogen bonds formed between MEK1 and the compounds. (**A**) 129696793, (**B**) 10813589, (**C**) 10991656, (**D**) 10524567, and (**E**) 10575055.

Molecular Dynamic (MD) simulation was performed to refine and assess the stability of protein after adding the missing loop (Supplementary Figure S3) and the binding stability of the protein–ligand complex system. The MD simulation evaluates and delineates the dynamic behavior of the ligand and the binding site residues. The best conformation flavonoids obtained from virtual screening for MEK1 inhibition advanced to MD simulation. The MD results were examined for RMSD of backbone, RMSD of heavy ligand atoms, RMSF values, hydrogen bonds number, and the radius of gyration to assess the stability of the protein–ligand complex. The RMSD value is a measure of how far a protein molecule deviates from its initial conformation over the course of the simulation. The RMSD values for the first rank were found to be within 0.2 nm for the backbone and 0.05–0.1 nm through the simulation, and the protein–ligand complex was considered to be stable (Figure 5A). The second rank RMSD value of backbone began at 0.2 nm then rose to 0.3 nm in 20 ns, then went back to stabilize at 0.2 nm. For the heavy atoms of ligand RMSD (Figure 5B), they showed values in a range of 0.05–0.1 nm then rose up to 0.25 nm after 20 ns to stabilize back at 0.15 nm. RMSD values of interaction of the third rank showed a very stable range, between 0.15 and 0.2 nm, throughout the 30 ns simulation for the backbone and 0.025–0.05 nm for the ligand’s heavy atoms. Fourth rank RMSD also showed fluctuations around 0.2–0.3 nm for the backbone and 0.025–0.05 nm for the ligand’s heavy atoms. The RMSD values of the fifth rank showed similar fluctuation, between 0.15 and 0.2 for the backbone and between 0.025 and 0.125 nm for the ligand’s heavy atoms. The low RMSD fluctuations indicate that the equilibration of a system is achieved through the simulation. The RMSF of the protein coordinates from their beginning positions for each residue was computed to define the flexible areas of the protein during the course of the simulation. The RMSF is a metric that measures how much ligand binding affects the flexibility of MEK1 residues. The RMSF values (Figure 5C) show that the maximum fluctuation was in the amino acid residue region 276–320, which is a proline rich loop and highly flexible region, and the fluctuation was within the range of 1.25 nm. From this perspective also, owing to low fluctuation in RMSF, the protein–ligand complex seems stable (Figure 5C). The radius of gyration is a measure of protein compactness, and the low fluctuations in its value indicate stability of the protein backbone. The radius of gyration fluctuates in a range of around 2.0 nm for all the four simulations and during the entire simulation, which points towards stability of the protein (Figure 5D). The presence of hydrogen bonds is critical for a protein complex’s stability. Analysis of the number of hydrogen bonds in (Figure 6) shows them appearing and disappearing during the course of simulation. Looking more closely at (Figure 6), the maximum number of hydrogen bonds and pairs reaches eight for first rank compound, five for the second, two for the third and fourth ranks, and eight for the fifth rank compound. Due to the isolated hydrogen bonds and low average hydrogen-bond number per time frame, the hydrogen-bond network in the complexes appeared to be weak. Other interactions were thought to hold hydrogen bonds in places where they had disappeared. As a result, no notable conformational changes in the complexes were observed across the simulated time period. These findings suggested that the MD simulation trajectory for the complex after equilibrium was reliable enough for future investigation.

## 3. Materials and Methods

### 3.1. Data Retrieval and Preparation

The three-dimensional structure coordinate of MEK1 complexed with the native inhibitor is retrieved from the protein data bank (PDB, https://www.rcsb.org/, accessed on 19 January 2022). The structure of MEK1 (PDB code: 1S9J) with 2.40 Å resolution [16] was selected and used for the study. MODLLER was used to model the missing prolin-rich loop in the crystallized structure (residues from 276 to 305), which is a high flexible region located after the catalytic site [71]. The unique allosteric site for MEK1 was verified [72], and the coordinates for the grid box covering the catalytic site were prepared using AutoDockTools-1.5.6 [73]. Other preparations included: deleting water, checking for missing atoms, removing heteroatoms, adding polar hydrogens, computing and adding charges, and finally converting the protein into a (pdbqt) file for the molecular docking, also performed using AutoDockTools-1.5.6 software package. A drug-like library prepared from PubChem (www.ncbi.nlm.nih.gov, accessed on 4 April 2021) and 2630 flavonoids were filtered to 1289 by 3D structure availability and the Lipinski rule of five [74]. Ligands were prepared for virtual screening using Open Babel command line [75] and converted from (sdf) file to (pdbqt) after adding charges and hydrogens. The 2-D illustrations for the chemical compounds were prepared using MarvinSketch v18.4, ChemAxon (http://www.chemaxon.com/products/marvin, accessed on 6 June 2021). 

### 3.2. Molecular Docking

Docking was carried out using AutoDock Vina [76] after preparing the configuration file with the details of the grid box coordinates, with energy range of 4 and maximum exhaustiveness of 24. Best mode, least RMSD, and highest docking affinity results were taken and ranked for MEK1. For further analysis of the docking, protein–ligand interaction plots of selected flavonoids with MEK1 was performed using Ligplot+ v1.4.5 [77] and illustrations of the docking were prepared using PyMOL v2.4.0 [78]. Further, calculations of binding energy and dissociation constant were performed by XScore v1.2.11 [79]. The degree of ligand filling the binding site was evaluated by loss in accessible surface area (ASA).

$$\Delta ASA_{i}=ASA_{i}^{protien}-ASA_{i}^{protien-ligand}$$

A residue is said to be taking part in filling the binding site if it loses more than 10 ${Å}^{2}$ ASA due to binding [80]. All the ASA calculations of the protein–ligand complexes and the unbound proteins were performed by Naccess v2.1.1 [81]. 

### 3.3. Drug-Likeness and Pharmacokinetics Prediction

The “pkCSM-pharmacokinetics” online web-server (http://biosig.unimelb.edu.au/pkcsm/, accessed on 20 July 2021) was used for predictions of drug-likeness and pharmacokinetic properties: Absorption, Distribution, Metabolism, Excretion, and Toxicity (ADMET) [82].

### 3.4. Molecular Dynamic Simulation

The docked protein–ligand complexes were subjected to energy minimization using Gromacs v2020.5 [83] with the CHARMM36 all atom force field. Ligand and protein were separated to add ligand hydrogen atoms using Avogadro v2018 (https://avogadro.cc/, accessed on 6 January 2022) and then converted for topology using CHARMM force field (https://cgenff.umaryland.edu/, accessed on 6 January 2022), then wrote back with the complex topology file. The models were solvated with a water model in a cubic periodic box with 1 nm distance from the edge of the complex atoms. The solvated system was neutralized by five sodium ions. Energy minimization was carried out through 50,000 steps. An equilibration was conducted by number of particles, volume, and temperature (NVT), and number of particles, pressure, and temperature (NPT) temperature was coupled for ligand, protein, solvent, and ions, separately. Then the system proceeded to the actual MD simulation. The final models obtained at the end of MD were validated and illustrated by VMD (https://www.ks.uiuc.edu/Research/vmd/, accessed on 5 September 2021). For the analysis, Gromacs and Xmgrace (https://plasma-gate.weizmann.ac.il/Grace/, accessed on 7 September 2021) were used. 

## 4. Conclusions

In conclusion, the molecular docking results suggest that some flavonoids could be better inhibitors of MEK1 compared to the native inhibitor based on the binding affinity and ligand interactions. The selected flavonoids could be potential drug candidates after re-engineering to improve the pharmacokinetic properties. Further, MD simulation studies with 100 ns time scale confirm the stability of the first rank flavonoid and MEK1 complex by root–mean–square deviation, root–mean–square fluctuation, and the radius of gyration. Our findings suggest that natural flavonoids are a promising and readily available source of anticancer targeted therapy in the future. However, these interpretations need further confirmatory analysis and validations for the screened molecules to ascertain their efficacy in the illness treatment.

## Figures and Tables

**Table 1:** The selected flavonoids and the binding strength score against MEK1 (Autodock Vina docking energy, X-Score binding energy and $pK_{d}$). The higher the absolute values of the scores, the better the binding.

Rank	Flavonoids	Docking Affinity (kcal/mol)	Binding Energy (Kcal/mol)	$pK_{d}$ $\mathrm{or}\;-\log(K_{d})$
1	129696793	−10.8	−10.25	7.52
2	10813589	−10.6	−10.96	8.03
3	10991656	−10.5	−9.49	6.95
4	10524567	−10.5	−10.83	7.94
5	10575055	−10.4	−10.11	7.41
Native		−9.0	−8.95	6.56

**Table 2:** The MEK1 residues interacting with selected flavonoid (CID: 129696793) are listed with the number of non-bonding interactions and Δ*ASA*.

Interacting Residues	Hydrogen Bonds	Non-Bonding Interactions	$\Delta ASA\;(\mathrm{In}\;{Å}^{2})$
Leu-115	0	1	3.89
Leu-118	0	5	17.12
Val-127	1	2	10.27
Gly-128	1	1	0.78
Phe-129	0	2	6.45
Ile-141	0	6	21.79
Arg-189	0	3	11.93
Asp-208	0	5	39.18
Phe-209	0	9	25.06
Gly-210	0	1	8.22
Val-211	0	4	4.59
Ser-212	0	4	2.41
Leu-215	0	4	14.8
Ile-216	0	9	25.95
Met-219	0	4	42.37
Arg-234	0	2	16.78

**Table 3:** The MEK1 residues interacting with selected flavonoid (CID: 10813589) are listed with the number of non-bonding interactions and Δ*ASA*.

Interacting Residues	Hydrogen Bonds	Non-Bonding Interactions	$\Delta ASA\;(\mathrm{In}\;{Å}^{2})$
Gly-79	0	3	17.63
Gly-80	1	4	15.78
Lys-97	1	8	31.09
Leu-98	0	1	0.55
Ile-99	0	15	27.12
His-100	0	2	16.96
Leu-115	0	2	3.89
Leu-118	0	3	16.47
Val-127	0	2	9.47
Gly-128	0	1	0.78
Phe-129	0	1	5.57
Ile-141	0	2	21.79
Asp-190	0	1	20.27
Asn-195	0	2	4.05
Asp-208	0	9	39.24
Phe-209	0	6	22.18
Val-211	0	3	4.59
Leu-215	0	1	12.45
Met-219	0	5	43.97
ATP	0	7	

**Table 4:** The MEK1 residues interacting with selected flavonoid (CID: 10991656) are listed with the number of non-bonding interactions and Δ*ASA*.

Interacting Residues	Hydrogen Bonds	Non-Bonding Interactions	$\Delta ASA\;(\mathrm{In}\;{Å}^{2})$
Leu-115	0	2	3.89
Leu-118	0	1	15.6
Ile-141	0	2	21.79
Met-143	0	1	9.92
Asp-190	0	4	27.2
Asp-208	0	13	40.1
Phe-209	0	10	24.33
Val-211	0	3	4.59
Ser-212	1	3	2.41
Leu-215	0	5	14.8
Ile-216	0	5	13.79
Met-219	0	5	46.4

**Table 5:** The MEK1 residues interacting with the selected flavonoid (CID: 10524567) are listed with the number of non-bonding interactions and Δ*ASA*.

Interacting Residues	Hydrogen Bonds	Non-Bonding Interactions	$\Delta ASA\;(\mathrm{In}\;{Å}^{2})$
Leu-118	0	6	16.62
Val-127	0	2	9.35
Gly-128	0	1	0.78
Phy-129	0	1	5.48
Ile-141	0	5	21.79
Met-143	0	6	9.92
Asp-190	0	2	30.23
Cys-207	0	2	4.33
Asp-208	0	11	38.96
Phe-209	0	14	24.77
Leu-215	0	4	14.8
Ile-216	0	3	21.97
Met-219	0	4	52.28

**Table 6:** The MEK1 residues interacting with selected flavonoid (CID: 10575055) are listed with the number of non-bonding interactions and Δ*ASA*.

Interacting Residues	Hydrogen Bonds	Non-Bonding Interactions	$\Delta ASA\;(\mathrm{In}\;{Å}^{2})$
Leu-118	0	4	16.73
Val-127	0	1	9.63
Ile-141	0	4	21.79
Met-143	0	3	9.92
Arg-189	0	3	14.72
Asp-190	1	4	41.14
Asp-208	0	9	40.01
Phe-209	0	11	25.06
Ile-216	0	1	18.14
Met-219	0	2	50.93
Arg-234	1	2	13.09
ATP	1	0	

**Table 7:** Structures and chemical properties of the selected flavonoids against MEK1 to predict the drug-likeness: molecular weight, lipophilicity (LogP), number of (#) rotatable bonds, hydrogen acceptors and hydrogen donors, and polar surface area.

Rank	Compound (CID)	Structure	Molecular Weight	LogP	#Rotatable Bonds	#Acceptors	#Donors	Surface Area
1	129696793	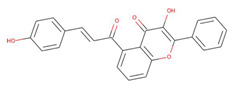	384.387	4.7673	4	5	2	165.349
2	10813589	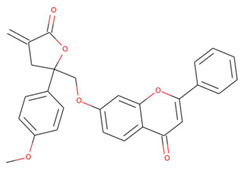	454.478	5.246	6	6	0	195.555
3	10991656	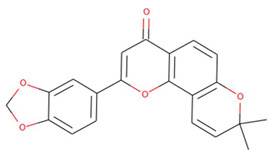	348.354	4.3729	1	5	0	148.966
4	10524567	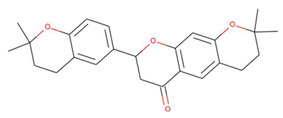	392.495	5.6003	1	4	0	171.717
5	10575055	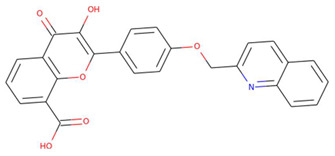	439.423	4.991	5	6	2	186.688

**Table 8:** ADMET properties of the selected flavonoids against MEK1.

Property	Model Name	Predicted Value					Unit
		129696793	10813589	10991656	10524567	10575055	
Absorption	Water solubility	−4.456	−5.914	−4.984	−5.161	−3.903	Numeric (log mol/L)
	Caco2 permeability	1.089	1.073	1.004	1.116	0.549	Numeric (log Papp in 10^−6^ cm/s)
	Intestinal absorption (human)	90.869	98.737	95.566	96.435	88.343	Numeric (% Absorbed)
	Skin Permeability	−2.735	−2.731	−2.589	−2.729	−2.734	Numeric (log Kp)
	P-glycoprotein substrate	Yes	No	Yes	No	Yes	Categorical (Yes/No)
	P-glycoprotein I inhibitor	Yes	Yes	Yes	Yes	No	Categorical (Yes/No)
	P-glycoprotein II inhibitor	Yes	Yes	Yes	Yes	Yes	Categorical (Yes/No)
Distribution	VDss (human)	−0.696	−0.157	0.121	0.363	−0.432	Numeric (log L/kg)
	Fraction unbound (human)	0.039	0.223	0.195	0.03	0.208	Numeric (Fu)
	BBB permeability	−0.371	−0.749	0.358	−0.005	−0.956	Numeric (log BB)
	CNS permeability	−1.883	−1.903	−1.595	−1.608	−2.889	Numeric (log PS)
Metabolism	CYP2D6 substrate	No	No	No	No	No	Categorical (Yes/No)
	CYP3A4 substrate	Yes	Yes	Yes	Yes	Yes	Categorical (Yes/No)
	CYP1A2 inhibitor	Yes	No	Yes	No	No	Categorical (Yes/No)
	CYP2C19 inhibitor	Yes	Yes	Yes	Yes	No	Categorical (Yes/No)
	CYP2C9 inhibitor	Yes	Yes	Yes	Yes	Yes	Categorical (Yes/No)
	CYP2D6 inhibitor	No	No	No	No	No	Categorical (Yes/No)
	CYP3A4 inhibitor	Yes	Yes	Yes	Yes	No	Categorical (Yes/No)
Excretion	Total Clearance	0.184	0.81	0.345	0.087	0.551	Numeric (log mL/min/kg)
	Renal OCT2 substrate	No	No	No	No	No	Categorical (Yes/No)
Toxicity	AMES toxicity	Yes	Yes	No	No	No	Categorical (Yes/No)
	Max. tolerated dose (human)	0.204	0.64	−0.242	−0.067	0.742	Numeric (log mg/kg/day)
	hERG I inhibitor	No	No	No	No	No	Categorical (Yes/No)
	hERG II inhibitor	Yes	Yes	No	No	Yes	Categorical (Yes/No)
	Oral Rat Acute Toxicity (LD50)	2.767	2.734	2.086	3.018	2.656	Numeric (mol/kg)
	Oral Rat Chronic Toxicity (LOAEL)	0.914	0.805	1.269	1.713	0.755	Numeric (log mg/kg_bw/day)
	Hepatotoxicity	Yes	No	No	No	Yes	Categorical (Yes/No)
	Skin Sensitization	No	No	No	No	No	Categorical (Yes/No)
	*T.Pyriformis* toxicity	0.29	0.287	0.555	0.491	0.285	Numeric (log ug/L)
	Minnow toxicity	0.09	−2.72	−0.483	−0.22	−1.62	Numeric (log mM)

## Data Availability

PDB, https://www.rcsb.org/, accessed on 19 January 2022; www.ncbi.nlm.nih.gov, accessed on 4 April 2021.

## Supplementary Materials

The following supporting information can be downloaded at: https://www.mdpi.com/article/10.3390/ph15020195/s1, Table S1: Autodock Vina docking results of flavonoids against MEK1 binding pocket. Figure S1: Two dimensional sketches of two stereoisomers (R & S) of 4^th^ rank compound (CID: 10524567). Figure S2: S-stereoisomer of (CID: 10524567) interaction with MEK1 compared to the native inhibitor, Table S2: MEK1 interaction residues with S-stereoisomer of (CID: 10524567), Figure S3: Modified MEK1 molecular dynamic (MD) simulation for 2 (ns).
