# Supplementary material for: Exploring the Natural Compounds in Flavonoids for Their Potential Inhibition of Cancer Therapeutic Target MEK1 Using Computational Methods
Source: Pharmaceuticals (Basel). 2022 Feb 3;15(2):195. doi: 10.3390/ph15020195 (PMC8876294; doi:10.3390/ph15020195)
Supplement: Supplementary file 1 [file pharmaceuticals-15-00195-s001.zip › Supplementary_file S2.pdf]

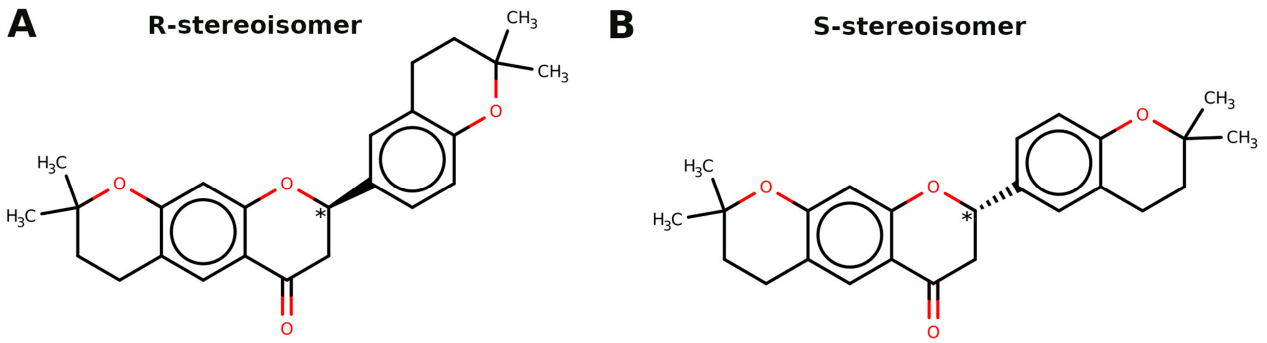

Figure S1. Two dimensional sketches of two stereoisomers (*R* & *S*) of 4th rank compound (CID: 10524567). The chiral carbon is marked with asterisk (*). (**A**) The *R*-stereoisomer with the bond attached to chiral carbon shown as solid (towards us). (**B**) The *S*-stereoisomer with the bond attached to chiral carbon as dashed (away from us).

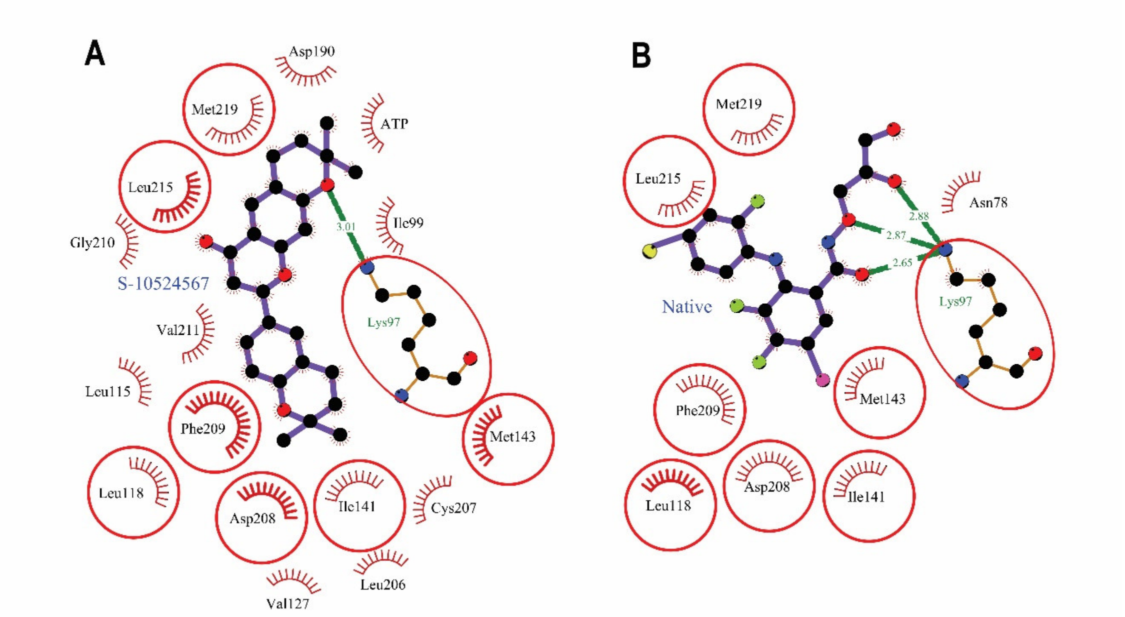

Figure S2. MEK1 protein-ligand interaction plots of the (*S*) configuration of (CID: 10524567) (**A**) and native inhibitor (**B**). The residues forming non-bonding interactions are shown as red bristles, while residues forming hydrogen bond and the bound ligand are shown as ball-and-stick representations. The carbon atoms are shown as black balls, nitrogen atoms as blue balls, oxygen atoms as red balls, fluorine atoms as green balls, bromine atom as pink ball, and iodine

atom as a yellow ball. The interacting residues common with those of the native inhibitor are shown in circle. The hydrogen bonds are shown as green dashed lines labeled with bond length (in Å).

Table S2. The MEK1 residues interacting with selected flavonoid (CID: 10524567) are listed with the number of non-bonding interactions and ΔASA.

**Interacting residues**	**Hydrogen bonds**	**Non-bonding interactions**	Δ*ASA* **(In Å^2)**
Lys-97	1	3	28.07
Ile-99	0	2	10.38
Leu-115	0	1	3.89
Leu-118	0	4	16.42
Val-127	0	4	9.28
Ile-141	0	5	21.79
Met-143	0	2	9.92
Asp-190	0	2	24.35
Leu-206	0	1	0
Cys-207	0	3	4.33
Asp-208	0	4	44.44
Phe-209	0	12	24.35
Gly-210	0	1	6.6
Val-211	0	2	4.59
Leu-215	0	3	14.8
Met-219	0	6	48.6
ATP	0	3	

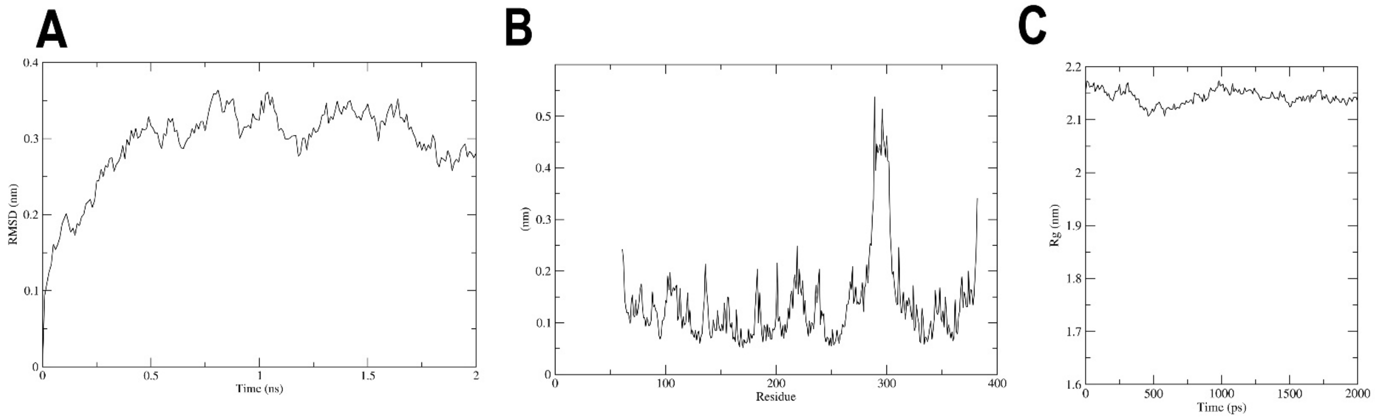

Figure S3. MEK1 modified protein molecular dynamic simulation for 2 (ns). (**A**) Root-mean-square deviation (RMSD) curve for the protein backbone. RMSD plot provides quantification

of the overall stability of the protein backbone during 2 ns simulation. From the graph we can see the values range between 0.1-0.35 nm. (**B**) Root-mean-square fluctuations (RMSF) curve of the MEK1 residues. The highest fluctuation was in the missing loop area up to 0.5 nm which is originally high flexible region and the others resides seem to be fluctuate in only around 0.2 nm (C): Radius of gyration (total) of the protein. The graph shows the protein gyrate only around 2.1–2.2 nm and no big changes through the 2 ns simulation which indicate the stability of the shape of protein.
