# Supplementary material for: Exploring the Natural Compounds in Flavonoids for Their Potential Inhibition of Cancer Therapeutic Target MEK1 Using Computational Methods
Source: Pharmaceuticals (Basel). 2022 Feb 3;15(2):195. doi: 10.3390/ph15020195 (PMC8876294; doi:10.3390/ph15020195)
Supplement: Supplementary file 1 [file pharmaceuticals-15-00195-s001.zip › Supplementary_file_S1.pdf]

Table S1. Autodock Vina docking results against MEK1 binding pocket.

CID number	SMILES	Docking affinity (kcal/mol)
129696793	o1c(c(c(=O)c2C(=[C][C]=[C]c12)C(=O)/[C]=[C]/C1=[C][C]=C([C]=[C]1)O)O)C1=[C][C]=[C][C]=[C]1	-10.8
10813589	O(C1=[C][C]=C2C(=O)[C]=C(OC2=[C]1)C1=[C][C]=[C][C]=[C]1)[C][C@@]1(OC(=O)C(=[C])[C]1)C1=[C][C]=C([C]=[C]1)O[C]	-10.6
10991656	O1C2=[C][C]=C3C(=O)[C]=C(OC3=C2[C]=[C]C1([C])[C])C1=[C]C2=C([C]=[C]1)O[C]O2	-10.5
10981007	O1C2=C3C(=[C][C]=C2C(=O)[C][C]1C1=[C][C]=C2C(=[C]1)O[C]O2)OC([C])([C])[C]=[C]3	-10.5
10524567	O1C2=C([C]=C3C(=[C]2)O[C]([C]C3=O)C2=[C]C3=C([C]=[C]2)OC([C][C]3)([C])[C])[C][C]C1([C])[C]	-10.5
11796528	C1(=[C][C]=C([C]=[C]1)O[C]C1=[C][C]=C2[C]=[C][C]=[C]C2=N1)C1=[C]C(=O)C2=C(O1)C(=[C][C]=[C]2)C(=O)O	-10.4
10575055	C1(=[C][C]=C([C]=[C]1)O[C]C1=[C][C]=C2[C]=[C][C]=[C]C2=N1)c1oc2c(c(=O)c1O)[C]=[C][C]=C2C(=O)O	-10.4
100941751	O1C2=C(C(=C(C(=[C]2)O)C2=[C][C]=C([C]=[C]2)O[C])O)C(=O)[C][C]1C1=[C][C]=C(C(=[C]1)O)O[C]	-10.3
100941748	O1C2=C(C(=C(C(=[C]2)O)C2=[C][C]=C([C]=[C]2)O[C])O)C(=O)[C][C]1C1=[C][C]=C([C]=[C]1)O	-10.3
23991884	o1c([C])c(c(=O)c2C(=[C]C(=[C]c12)O[C]C1=[C][C]=C(O1)C(=O)O)O)C1=[C][C]=[C][C]=[C]1	-10.3
180439	O1C(=[C]C(=O)C2=C1[C]=C(C(=[C]2)[C][C]=C([C])[C])O)C1=[C]C2=C([C]=[C]1)O[C]O2	-10.3
100943380	C1(=[C][C]=[C]C(=[C]1)C1=[C]C(=O)C2=[C][C]=[C][C]=C2O1)S(=O)(=O)NC1=[C][C]=C([C])[C]=[C]1	-10.2
53982668	O1C2=[C][C]=[C][C]=C2C(=O)/C(=[C]\N[C]C2=[C][C]=C([C]=[C]2)C(=O)O)/[C]1C1=[C][C]=[C][C]=[C]1	-10.2
11247668	O1C2=[C]C(=C(C(=C2C(=O)[C][C]1C1=[C][C]=C(C(=[C]1)O)O)O)[C]/[C]=C(\[C])/[C][C][C](C(=[C])[C])O)O	-10.2
10338671	C1(=[C][C]=C2C(=O)[C]=C(OC2=[C]1)C1=[C][C]=[C][C]=[C]1)O[C][C@]1(OC(=O)C(=[C])[C]1)[C]	-10.2
129853633	o1c(c(c(=O)c2C(=[C][C]=[C]c12)C(=O)/[C]=[C]/C1=[C][C]=C(C(=[C]1)O)O)O)C1=[C][C]=[C][C]=[C]1	-10.1
42608050	O1C([C])([C])[C]=[C]C2=C3C(=[C]C(=C12)[C]1[C]C(=O)C2=C([C]=C([C]=C2O1)O)O)[C]=[C]C([C])([C])O3	-10.1
42608003	O1c2c(c3c(C(=O)[C][C](O3)C3=[C][C]=C(C(=[C]3)O)O)c3OC([C])([C])[C][C]c23)[C][C]C1([C])[C]	-10.1
12019529	O1C(=[C]C(=O)C2=C([C]=C([C]=C12)OC(=O)[C]C1=[C][C]=[C][C]=[C]1)O)C1=[C][C]=[C][C]=[C]1	-10.1
100986463	O1C(=[C]C(=O)C2=C1[C]=C([C]=C2O)OC(=O)[C]/[C]=[C]/C1=[C][C]=[C][C]=[C]1)C1=[C][C]=[C][C]=[C]1	-10

**85823044**	O1C(=[C]C(=O)C2=C([C]=[C][C]=C12)O[C]C1=[C][C]=[C][C]=[C]1)C1=[C][C]=C([C]=[C]1)[C]=O	-10
**42607995**	O1c2c(c(c(c(c2[C][C](C(=[C])[C])O)O)[C][C]=C([C])[C])O)C(=O)[C][C]1C1=[C][C]=C(C(=[C]1)O)O	-10
**24133364**	o1c([C])c(c(=O)c2[C]=[C]C(=[C]c12)O[C]C1=[C][C]=C(O1)C(=O)O)C1=[C][C]=[C][C]=[C]1	-10
**907980**	o1c([C])c(c(=O)c2=[C][C]=C([C]=c12)O[C]C1=[C][C]=[C][C]=[C]1)C1=[C][C]=[C][C]=[C]1	-10
**101974135**	O1C2=C(C(=[C]C(=C2C(=O)[C][C]1C1=[C][C]=[C][C]=[C]1)O)O)[C]c1c(=O)oc(c([C])c1O)[C][C]	-9.9
**73265448**	O1[C]=C(C(=O)C2=C1[C]=C([C]=C2O)O[C]C1=[C][C]=[C][C]=[C]1)C1=[C][C]=C([C]=[C]1)N(=O)=O	-9.9
**56649181**	C1(=C([C]=C2C(=C1O)C(=O)[C]=C(O2)C1=[C][C]=[C][C]=[C]1)O)O[C]C1=[C][C]=C(F)[C]=[C]1	-9.9
**54721824**	O1C2=C(C(=[C]C(=C2C(=O)[C][C]1C1=[C][C]=[C][C]=[C]1)O)O)[C]c1c(=O)oc(c([C])c1O)[C][C]	-9.9
**44382145**	C1(=[C]C2=C(C(=[C]1)O)C(=O)[C]=C(O2)C1=[C][C]=[C][C]=[C]1)OC(=O)C1=[C][C]=C(F)[C]=[C]1	-9.9
**23644952**	c1(c(c(=O)c2[C]=[C]C(=[C]c2o1)O[C]C1=N[C]=[C][C]=[C]1)C1=[C][C]=[C][C]=[C]1)S[C]C1=[C][C]=N[C]=[C]1	-9.9
**154699985**	O(C1=[C][C]=C2C(=O)[C][C](OC2=[C]1)C1=[C][C]=[C][C]=[C]1)[C]1O[C]([C]([C]([C]1O)O)O)C(=O)O	-9.8
**101685222**	O1C2=[C]C(=C(C(=C2C(=O)[C]([C]1C1=[C][C]=C([C]=[C]1)O)O)O)[C]C1=[C][C]=C([C]=[C]1)O)O	-9.8
**101245387**	C1(=c2c(=C([C]=C1O)O)c(=O)c(c(o2)C1=[C][C]=C([C]=[C]1)O[C])O)O[C][C]1O[C]1C1=[C]C(=C([C]=[C]1)O)O	-9.8
**100941747**	O1C2=[C]C(=C(C(=C2C(=O)[C][C]1C1=[C][C]=C([C]=[C]1)O)O)C1=[C][C]=[C][C]=[C]1)O	-9.8
**44382144**	O1C(=[C]C(=O)C2=C1[C]=C([C]=C2O)OC(=O)C1=[C][C]=C([C])[C]=[C]1)C1=[C][C]=[C][C]=[C]1	-9.8
**10551087**	O1C2=C3C(=[C]C(=C2[C][C](C1([C])[C])O)[C]1[C]C(=O)C2=C([C]=C([C]=C2O1)O)O)[C][C]C([C])([C])O3	-9.8
**1522807**	O1[C]=C(C(=O)C2=[C][C]=C([C]=C12)O[C]C1=[C][C]=C([C]=[C]1)N(=O)=O)C1=[C][C]=[C][C]=[C]1	-9.8
**491718**	O1c2c([C])c(c(c(c2C(=O)[C][C]1C1=[C][C]=C([C]=[C]1)O)O)[C][C]=C([C])[C])O	-9.8
**154699598**	O([C]1O[C]([C]([C]([C]1O)O)O)C(=O)O)C1=[C][C]=[C][C]=C1C1=[C]C(=O)C2=[C][C]=[C][C]=C2O1	-9.7
**76333661**	O(C1=[C][C]=C2C(=O)[C]=C(OC2=[C]1)C1=[C][C]=[C][C]=[C]1)[C]1O[C]([C]([C]([C]1O)O)O)[C]C(=O)[C]	-9.7
**42607799**	O1C2=C(C(=[C][C]=C2C(=O)[C][C]1C1=[C][C]=[C][C]=[C]1)O[C][C]=C([C])[C])/[C]=[C]/C([C])([C])O	-9.7
**24861960**	O1C2=[C]C(=C(C(=C2C(=O)[C][C]1C1=[C][C]=C(C(=[C]1)O[C])O)O)[C]/[C]=C(\[C])/[C]/[C]=[C]/C([C])([C])O)O	-9.7
**23644933**	o1c2=[C]C(=[C][C]=c2c(=O)c(c1N1[C]=N[C]=[C]1)C1=[C][C]=[C][C]=[C]1)O[C]C1=[C][C]=[C][C]=[C]1	-9.7
**19835337**	O(C1=[C]C2=C([C]=[C]1)C(=O)[C]=C(O2)C1=[C][C]=[C][C]=[C]1)[C][C](O)[C]N[C][C][C][C]C1=[C]N=[C][C]=[C]1	-9.7

**16737249**	O1C2=C(C(=[C]C(=[C]2)O)O)C(=O)[C][C]1C1=[C]C2=C([C]=[C]1)OC([C]=[C]2)([C])[C]	-9.7
**14304997**	C(=O)(N[C][C][C][C]N1[C][C]N([C][C]1)C1=N[C]=[C][C]=[C]1)c1c(=O)c2c(oc1C1=[C][C]=[C][C]=[C]1)[C]=[C][C]=[C]2	-9.7
**12109666**	O1C2=C3C(=[C][C]=C2C(=O)[C][C]1C1=[C][C]=C([C]=[C]1)O[C])OC([C])([C])[C]=[C]3	-9.7
**11742974**	c1(c2c([C]=[C]C([C])([C])O2)c(c2C(=O)[C][C](Oc12)C1=[C][C]=C([C]=[C]1)O)O)[C][C]1OC1([C])[C]	-9.7
**11358607**	O1C2=C(C3=C([C]=[C]2)C(=O)[C]=C(O3)C2=[C][C]=[C][C]=[C]2)[C]=[C]C1([C])[C]	-9.7
**1760386**	O1[C]=C(C(=O)C2=[C][C]=C([C]=C12)O[C]C1=[C][C]=[C][C]=[C]1)C1=[C][C]=C([C]=[C]1)O[C]	-9.7
**101938669**	C1(=[C][C]=C([C]=[C]1)c1c(c(=O)c2[C]=[C][C]=[C]c2o1)O)N1O[C][C]O[C][C]O[C][C]O[C][C]O[C]1	-9.6
**101238241**	C1(=[C]C2=C(C(=O)C(=[C]O2)C2=[C][C]=C([C]=[C]2)O)[C]=C1C(=O)[C])O[C]1O[C]([C]([C]([C]1O)O)O)[C]O	-9.6
**44559902**	O1C2=[C]C(=C(C(=C2C(=O)[C]([C]1C1=[C][C]=C([C]=[C]1)O)O)O)[C]C1=[C][C]=C([C]=[C]1)O)O	-9.6
**15383190**	O1C2=[C]C(=C3C(=O)[C]=C(OC3=C2[C]=[C]C1([C])[C])C1=[C][C]=[C][C]=[C]1)O	-9.6
**11582389**	C1(=[C]C2=C([C]=[C]1)C(=O)[C]=C(O2)C1=[C][C]=[C][C]=[C]1)O[C]1O[C]([C]([C]([C]1O)O)O[C])[C]O	-9.6
**11395168**	O1C(=[C]C(=O)C2=C1[C]=C([C]=C2O)O[C]C1=[C][C]=[C][C]=[C]1)C1=[C]C(=C([C]=[C]1)O[C])O	-9.6
**11314387**	O1C(=[C]C(=O)C2=C(C(=C([C]=C12)O)O[C]C1=[C][C]=[C][C]=[C]1)O)C1=[C][C]=[C][C]=[C]1	-9.6
**10449654**	O1C2=C(C(=C3C(=[C]2)OC(=[C]C3=O)C2=[C][C]=C([C]=[C]2)O)O)[C]=[C]C1([C])[C]	-9.6
**563997**	O1[C]=C(C(=O)C2=[C][C]=C([C]=C12)O[C]C1=[C][C]=[C][C]=[C]1)C1=[C][C]=[C][C]=[C]1	-9.6
**440215**	C1(=[C][C]=C2C(=O)C(=[C]OC2=[C]1)C1=[C][C]=[C][C]=[C]1)O[C]1O[C]([C]([C]([C]1O)O)O)[C]O	-9.6
**154699552**	O([C]1O[C]([C]([C]([C]1O)O)O)C(=O)O)C1=[C][C]=[C]C(=[C]1)C1=[C]C(=O)C2=[C][C]=[C][C]=C2O1	-9.5
**129881269**	o1c2=[C][C]=C([C]=c2c(=O)c(c1C1=[C]C2=C([C]=[C]1)O[C]O2)O)C(=O)O	-9.5
**129867277**	O1C(=[C]C(=C2[C]=[C][C]=[C][C]12)[N]NC(=O)C1=[C][C]=[C][C]=[C]1)C1=[C][C]=[C][C]=[C]1	-9.5
**129834514**	O1C2=[C]C(=[C]C(=C2C(=O)[C][C]1C1=[C][C]=C([C]=[C]1)OC(=O)[C])O)O[C]C1=[C][C]=[C][C]=[C]1	-9.5
**129791328**	C1(=[C]c2c([C]=[C]1)c(=O)c(c(o2)C(=O)O)C1=[C][C]=[C][C]=[C]1)O[C]c1sc2[C]=[C][C]=[C]c2n1	-9.5
**122366411**	C1(=[C][C]=C([C]=[C]1)C1=[C]C(=O)C2=[C][C]=[C][C]=C2O1)OS([O])([O])C(F)(F)F	-9.5

**102577930**	C1(=[C]C(=C2C(=O)[C]=C(OC2=[C]1)C1=[C][C]=C([C]=[C]1)O)O)OS([O])([O])C1=[C][C]=C([C])[C]=[C]1	-9.5
**54490901**	O1C2=[C][C]=[C][C]=C2C(=O)/C(=[C]\C2=[C][C]=[C]C(=[C]2)NC(=O)[C])/[C]1C1=[C][C]=[C][C]=[C]1	-9.5
**53249095**	O1C2=[C]C3=C([C]=[C]C([C])([C])O3)C(=C2C(=O)[C][C]1C1=C([C]=C([C]=[C]1)O)O)O	-9.5
**44584124**	O1C2=[C]C3=C([C]=[C]C([C])([C])O3)C(=C2C(=O)[C][C]1C1=C([C]=[C][C]=[C]1)O)O	-9.5
**25070110**	O1C2=[C]C(=[C]C(=C2C(=O)[C][C]1C1=[C][C]=C([C]=[C]1)O)O)O[C]C1=[C][C]=[C][C]=[C]1	-9.5
**23644934**	o1c2=[C]C(=[C][C]=c2c(=O)c(c1N1[C]=N[C]=[C]1)C1=[C][C]=C([C]=[C]1)O[C])O[C]C1=[C][C]=[C][C]=[C]1	-9.5
**15939984**	o1c2=[C][C]=C([C])[C]=c2c(=O)c(c1C1=[C][C]=C2C(=[C]1)O[C]O2)O	-9.5
**15342845**	o1c(c(c(=O)c2=C([C]=C([C]=c12)O)OC(=O)C1=[C][C]=[C][C]=[C]1)O[C])C1=[C][C]=[C][C]=[C]1	-9.5
**14626316**	O(C1=[C][C]=C2C(=O)[C]=C(OC2=[C]1)C1=[C][C]=[C][C]=[C]1)[C][C](O)[C]N[C][C]C1=[C][C]=C([C]=[C]1)O	-9.5
**14408549**	[C](OC1=[C][C]=C2C(=O)[C]=C(OC2=[C]1)C1=[C][C]=[C][C]=[C]1)[C](O)[C]N[C][C]C1=[C][C]=C(C(=[C]1)O[C])O[C]	-9.5
**12071400**	C1(=[C]C(=[C]c2oc(c(c(=O)c12)O)C1=[C]C(=C([C]=[C]1)O[C])O[C])O[C])OS([O])([O])C1=[C][C]=C([C]=[C]1)[C]	-9.5
**11718263**	C1(=[C][C]=C2C(=[C]1)C(=O)[C]=C(O2)C1=[C]C(=C([C]=[C]1)O)O)O[C][C](O)[C]NC([C])([C])[C]	-9.5
**625630**	O1C(=[C]C(=O)C2=C1C(=[C]C1=C2[C]=[C][C]=[C]1)O)C1=[C][C]=[C][C]=[C]1	-9.5
**440195**	C1(=[C]C2=C([C]=[C]1)C(=O)[C][C](O2)C1=[C][C]=[C][C]=[C]1)O[C]1O[C]([C]([C]([C]1O)O)O)[C]O	-9.5
**102203496**	O1C(=[C]C(=O)C2=C([C]=C([C]=C12)O[C]O[C])O[C]O[C])C1=[C][C]=C([C]=[C]1)O[C]C1=[C][C]=[C][C]=[C]1	-9.4
**101887721**	C1(=C([C]=C(C2=C1OC(=[C]C2=O)C1=[C][C]=C([C]=[C]1)O)O)O[C])[C]1O[C]([C])[C](C(=O)[C]1)O	-9.4
**101396888**	O1c2c3C(=O)C(=[C]Oc3c(c(c2[C]=[C]C1([C])[C])O)[C][C](C([C])([C])O)O)C1=[C][C]=C([C]=[C]1)O	-9.4
**69941512**	C1(=[C]C2=C([C]=[C]1)OC(=[C]C2=O)C1=[C][C]=[C][C]=[C]1)O[C][C][C]1OC(=O)C(=C1O)O	-9.4
**56589538**	C1(=C(C(=C2C(=O)[C]=C(OC2=[C]1)C1=[C][C]=[C][C]=[C]1)O)O)O[C]C1=[C][C]=C(F)[C]=[C]1	-9.4
**42607962**	O1C2=C(C(=[C]C3=C2[C]=[C]C(O3)([C])[C])O)C(=O)[C][C]1C1=[C][C]=C([C]=[C]1)O[C]	-9.4
**15342843**	o1c(c(c(=O)c2C(=[C]C(=[C]c12)OC(=O)C1=[C][C]=[C][C]=[C]1)O)O[C])C1=[C][C]=[C][C]=[C]1	-9.4
**15108704**	O1C(=[C]C(=O)C2=C(C(=C([C]=C12)O[C]C1=[C][C]=[C][C]=[C]1)O)O)C1=[C][C]=C(C(=[C]1)O)O	-9.4
**14187087**	O1C2=C(C(=C3C(=[C]2)OC([C]=[C]3)([C])[C])O)C(=O)[C][C]1C1=[C][C]=C([C]=[C]1)O	-9.4
**11247278**	O1C2=C(C(=[C]C(=C2[C][C]=C([C])[C])OC(=O)[C])O)C(=O)[C][C]1C1=[C][C]=C([C]=[C]1)OC(=O)[C]	-9.4
**5466794**	C1(=[C]C(=O)C2=C(C(=C([C]=C2O)O)[C]2[C][C]N([C])[C][C]2O)O1)C1=C(Cl)[C]=[C][C]=[C]1	-9.4

**629905**	C1(=[C][C]=C(C(=[C]1)O[C]C1=[C][C]=[C][C]=[C]1)O[C])c1oc2=[C]C(=[C][C]=c2c(=O)c1O[C])O[C]	-9.4
**154699919**	O([C]1O[C]([C]([C]([C]1O)O)O)C(=O)O)C1=[C][C]=C([C]=[C]1)C1=[C]C(=O)C2=C([C]=[C][C]=[C]2)O1	-9.3
**131751372**	O1C2=C3[C]=[C]C([C])([C])OC3=[C][C]=C2C(=O)[C]([C]1C1=[C][C]=C(C(=[C]1)[C][C]=C([C])[C])O)O	-9.3
**100986464**	O1C(=[C]C(=O)C2=C1[C]=C([C]=C2O)OC(=O)[C]/[C]=[C]/C1=[C][C]=C([C]=[C]1)O[C])C1=[C][C]=[C][C]=[C]1	-9.3
**69567244**	O1C(=[C]C(=O)C2=C1[C]=C([C]=C2O)O[C]C1=[C][C]=[C][C]=[C]1)C1=[C][C]=C([C]=[C]1)O	-9.3
**14626328**	C1(=[C][C]=C2C(=O)[C]=C(OC2=[C]1)C1=[C][C]=[C][C]=[C]1)O[C][C](O)[C][C][C]O[C]1O[C][C][C][C]1	-9.3
**14304994**	c1(c(=O)c2[C]=[C][C]=[C]c2oc1C1=[C][C]=[C][C]=[C]1)C(=O)N1[C][C]N([C][C]1)[C]C1=[C][C]=[C][C]=[C]1	-9.3
**12019528**	O1C(=[C]C(=O)C2=C([C]=C([C]=C12)OC(=O)C1=C([C]=[C][C]=[C]1)OC(=O)[C])O)C1=[C][C]=[C][C]=[C]1	-9.3
**11995581**	C1(=[C]C(=C(C(=[C]1)[C][C]=C([C])[C])O[C])/[C]=[C]/C([C])([C])O)[C]1OC2=[C]C(=[C][C]=C2C(=O)[C]1)O	-9.3
**11132477**	o1c2=[C][C]=[C][C]=c2c(=O)c(c1C1=[C][C]=[C][C]=[C]1)[C](C1=[C][C]=C([C]=[C]1)N(=O)=O)O	-9.3
**637111**	O1c2c(c(c3[C]=[C]C([C])([C])Oc3c2[C][C]=C([C])[C])O)C(=O)[C]([C]1C1=C([C]=[C][C]=[C]1)O)O	-9.3
**636525**	O1C2=C(C(=[C]C3=C2[C]=[C]C(O3)([C])[C])O)C(=O)[C][C]1C1=[C]C(=C([C]=[C]1)O[C])O	-9.3
**563887**	C1(=[C][C]=C(C(=[C]1)O[C])O[C]C1=[C][C]=[C][C]=[C]1)c1oc2[C]=[C][C]=[C]c2c(=O)c1O[C]	-9.3
**49700**	C1(=c2c(=[C][C]=C1O[C])c(=O)c([C])c(o2)C1=[C][C]=[C][C]=[C]1)[C]N1[C][C]O[C][C]1	-9.3
**137350369**	C1(=[C][C]=[C]C2=C1O[C]=C(C2=O)C1=[C][C]=[C][C]=[C]1)[C]1O[C]([C]([C]([C]1O)O)O)[C]O	-9.2
**129856151**	O(c1c(=O)c2=[C][C]=[C][C]=c2oc1C1=[C][C]=[C][C]=[C]1)C(=O)[C]1O[C]([C]([C]([C]1O)O)O)O	-9.2
**101887720**	C1(=C([C]=C(C2=C1OC(=[C]C2=O)C1=[C][C]=C([C]=[C]1)O)O)O)[C]1O[C]([C])[C]([C]([C]1)O)O	-9.2
**101887719**	C1(=C([C]=C(C2=C1OC(=[C]C2=O)C1=[C][C]=C([C]=[C]1)O)O)O[C])[C]1O[C]([C])[C]([C]([C]1)O)O	-9.2
**101429132**	C1(=[C]C(=C(C(=[C]1)/[C]=[C]/C([C])([C])O)O[C])[C][C]=C([C])[C])[C]1OC2=C([C]=[C]C(=[C]2)O)C(=O)[C]1	-9.2
**101214939**	C1(=[C][C]=C([C]=[C]1)c1c(c(=O)c2[C]=[C][C]=[C]c2o1)O)N1[C][C]N([C][C]1)C1=C([C]N[C]=[C]1)S([O])([O])O	-9.2
**52951514**	C1(=[C]C(=C([C]=[C]1)O[C][C](C([C])([C])O)O)O[C])C1=[C]OC2=[C]C3=C([C]=C2C1=O)[C]=[C]C(O3)([C])[C]	-9.2

**52951513**	O1C2=[C]C3=C(C(=O)C(=[C]O3)C3=[C][C]=C(C(=[C]3)O[C])O[C][C](C([C])([C])O)O)C(=C2[C]=[C]C1([C])[C])O[C]	-9.2
**42607996**	O1C2=C(C(=[C]C(=C2C(=O)[C][C]1C1=[C]C(=C([C]=[C]1)O)O)O)O)[C]/[C]=C(\[C])/[C]OC(=O)[C]	-9.2
**25030431**	o1c(c(c(=O)c2=[C]C(=[C][C]=c12)NC(=O)[C][C]C(=O)O)O)C1=[C]C(=C([C]=[C]1)O)O	-9.2
**24960482**	C1(=[C]C2=C([C]=[C]1)C(=O)[C]=C(O2)C1=[C]C(=[C]C(=[C]1)O)O)O[C][C](O)[C]N[C]([C])[C]	-9.2
**15308126**	C1(=c2c(=[C]C(=C1O)O[C])oc(c(c2=O)O)C1=[C][C]=C([C]=[C]1)O[C])OS([O])([O])C1=[C][C]=C([C])[C]=[C]1	-9.2
**14445197**	C1(=C([C]=C2C(=C1O)C(=O)[C]=C(O2)C1=[C][C]=C(C(=[C]1)O)O[C])O)[C]1O[C]([C])[C]([C]([C]1)O)O	-9.2
**12043067**	C1(=[C]C(=O)C2=C([C]=C(C(=C2O1)[C]1C(=O)[C]N([C])[C][C]1)O)O)C1=[C][C]=[C][C]=C1Cl	-9.2
**11597157**	C1(=[C]C2=C([C]=[C]1)C(=O)[C]=C(O2)C1=[C][C]=C([C]=[C]1)O)O[C]1O[C]([C]([C]([C]1O)O)O[C])[C]O	-9.2
**11545770**	C1(=[C][C]=C2C(=O)[C]=C(OC2=[C]1)C1=[C]C(=[C]C(=[C]1)O)O)O[C][C](O)[C]NC([C])([C])[C]	-9.2
**10741771**	O1C2=C(C(=C([C]=C2[C][C](C1([C])[C])O)[C]1[C]C(=O)C2=C([C]=C([C]=C2O)O)O1)[C][C]=C([C])[C])O	-9.2
**10291777**	O1C2=C(C3=C([C]=[C]2)C(=O)[C][C](O3)C2=C([C]=C([C]=[C]2)O)O)[C][C]1C([C])([C])O	-9.2
**5481228**	O1[C]=C(C(=O)C2=C([C]=C([C]=C12)O)O)C1=C([C]=C(C(=[C]1)[C][C]=C([C])[C])O)O	-9.2
**5378307**	O1C(=[C]C(=O)C2=C1[C]=[C]C1=C2[C]=C([C]=[C]1)O)C1=[C][C]=[C][C]=[C]1	-9.2
**564150**	o1c(c(c(=O)c2=[C][C]=C([C]=c12)O[C]C1=[C][C]=[C][C]=[C]1)O[C])C1=[C]C(=C([C]=[C]1)O[C])O[C]	-9.2
**511789**	O1C2=C(C(=[C][C]=C2C(=O)[C][C]1C1=[C][C]=C([C]=[C]1)O)O[C])[C][C]=C([C])[C]	-9.2
**177731**	O1[C]=C(C(=O)C2=C1[C]=C([C]=[C]2)O[C][C]=C([C])[C])C1=[C][C]=C([C]=[C]1)O[C]	-9.2
**11790**	O1C(=[C]C(=O)C2=[C][C]=C3[C]=[C][C]=[C]C3=C12)C1=[C][C]=[C][C]=[C]1	-9.2
**129834517**	O1C2=C(C(=[C]C(=[C]2)O[C]C2=[C][C]=[C][C]=[C]2)O[C])C(=O)[C][C]1C1=[C][C]=C([C]=[C]1)OC(=O)[C]	-9.1
**129822413**	O1C(=[C]C(=O)C2=C1C(=[C]C(=[C]2)OC(=O)[C])N)C1=[C][C]=C([C]=[C]1)OC(=O)[C]	-9.1
**129716120**	c1(c(=O)c2=[C][C]=[C][C]=c2oc1C1=[C][C]=[C][C]=[C]1)C(=O)[C]1O[C]([C]([C]([C]1O)O)O)O	-9.1
**129703940**	o1c(c(c(=O)c2C(=[C][C]=[C]c12)C(=O)C1=[C]C(=C(C(=[C]1)O)O)O)O)C1=[C][C]=[C][C]=[C]1	-9.1
**101499234**	C1(=[C]C(=C(C(=[C]1)O[C])O[C]([C]O)[C](C1=[C][C]=C([C]=[C]1)O)O)O[C])[C]1OC2=[C]C(=[C]C(=C2C(=O)[C]1)O)O	-9.1
**101396887**	O1c2c3C(=O)C(=[C]Oc3c(c(c2[C][C]1C([C])([C])O)O)[C][C]=C([C])[C])C1=[C][C]=C([C]=[C]1)O	-9.1
**95400994**	O1C2=[C][C]=C3[C]=[C][C]=[C]C3=C2C(=O)[C][C]1C1=[C][C]=[C][C]=[C]1	-9.1

**67984060**	o1c(c(c(=O)c2C(=[C]C(=[C]c12)O[C]C1=[C][C]=[C][C]=[C]1)O)O)C1=[C][C]=C(C(=[C]1)O)O	-9.1
**46881227**	C1(=[C]C2=C(C(=[C]1)O)C(=O)[C][C](O2)C1=[C][C]=[C][C]=[C]1)O[C]1O[C]([C]([C]([C]1O)O)O)[C]O	-9.1
**42608102**	O1c2c3[C]=[C]Oc3c(c(c2C(=O)[C][C]1C1=[C][C]=C2C(=[C]1)O[C]O2)O)O[C]	-9.1
**42608049**	O1C2=[C]C(=C3C(=O)[C][C](OC3=C2[C][C]C1([C])[C])C1=C([C]=C([C]=[C]1)O)O)O	-9.1
**24866261**	C1(=[C]C(=C2C(=O)[C]=C(OC2=[C]1)C1=[C][C]=[C][C]=[C]1)O)O[C]1O[C]([C]([C]([C]1O)O)O[C])[C]O	-9.1
**21576252**	O1[C]=C(C(=O)C2=C([C]=C([C]=C12)O[C][C]=C([C])[C])O)C1=[C][C]=C([C]=C1O)O	-9.1
**15118758**	C1(=[C][C]=C2C(=O)[C]=C(OC2=[C]1)C1=C([C]=[C][C]=[C]1)O)O[C]1O[C]([C]([C]([C]1O)O)O)[C]O	-9.1
**15108702**	O1C(=[C]C(=O)C2=C(C(=C([C]=C12)O[C]C1=[C][C]=[C][C]=[C]1)O)O)C1=[C][C]=[C][C]=[C]1	-9.1
**12428921**	O1C2=[C][C]=[C][C]=C2C(=O)/C(=[C]\C2=[C][C]=[C]C(=[C]2)O)/[C]1C1=[C][C]=[C][C]=[C]1	-9.1
**11618226**	C1(=[C][C]=C2C(=O)[C]=C(OC2=[C]1)C1=[C][C]=C(C(=[C]1)O)O)O[C][C](O)[C]NC([C])([C])[C]	-9.1
**11222065**	o1c2=[C][C]=[C][C]=c2c(=O)c(c1C1=[C][C]=[C][C]=[C]1)[C](C1=[C][C]=C([C]=[C]1)O[C])O	-9.1
**11077963**	o1c2=[C][C]=[C][C]=c2c(=O)c(c1C1=[C][C]=[C][C]=[C]1)[C](C1=[C][C]=[C][C]=[C]1)O	-9.1
**11048450**	O1C([C])([C])[C]=[C]c2c(c3C(=O)[C][C](Oc3c(c12)[C][C](C([C])([C])O)O)C1=[C][C]=C([C]=[C]1)O)O	-9.1
**10970624**	O1c2c(c3c([C]=[C]C([C])([C])O3)c(c2C(=O)[C][C]1C1=[C][C]=C([C]=[C]1)O)O)[C]O	-9.1
**5494866**	O1[C]=C(C(=O)C2=C([C]=C([C]=C12)O)O)C1=[C][C]=C(C(=[C]1)[C][C]=C([C])[C])O	-9.1
**5316782**	O1C2=C(C3=C(C(=[C]2)O)C(=O)[C][C](O3)C2=[C][C]=C([C]=[C]2)O)[C][C]1C([C])([C])O	-9.1
**3085261**	C1(=[C]C2=C([C]=[C]1)C(=O)C(=[C]O2)C1=[C]C2=C([C]=[C]1)O[C]O2)O[C]1O[C]([C]([C]([C]1O)O)O)[C]O	-9.1
**139593877**	O1C(=[C]C(=O)C2=C1C(=[C]C(=[C]2)[C]C(=O)[C])O[C])C1=[C][C]=C([C]=[C]1)O[C]	-9
**129847817**	O1C(=[C]C(=O)C2=C1[C]=C(C(=C2O)[C][C]C(=[C])[C])O)C1=C([C]=C([C]=[C]1)O)O	-9
**129735869**	O1C2=C([C]=[C][C]=C2C(=O)[C][C]1C1=[C][C]=[C][C]=[C]1)/[C]=[C]/C(=[C])[C]	-9
**102005382**	C1(=[C]C(=O)C2=C(C(=C([C]=C2O[C])O[C])C2=[C][C]N([C])[C][C]2)O1)C1=C(Cl)[C]=C([C]=[C]1)Cl	-9
**101999902**	C1(=[C]OC2=[C]C(=C(C(=C2C1=O)O)[C][C]C([C])([C])O)O)C1=[C][C]=C2OC([C]=[C]C2=C1O)([C])[C]	-9
**101650843**	O1C2=[C][C]=[C][C]=C2C(=O)/C(=[C]\C2=[C][C]=[C]C(=[C]2)N(=O)=O)/[C]1C1=[C][C]=[C][C]=[C]1	-9

**101516665**	O1C2=[C]C(=[C]C(=C2C(=O)[C][C]1C1=[C][C]=C([C]=[C]1)OC(=O)[C])O)O[C][C]=C([C])[C]	-9
**74333987**	O1C2=C(C(=C(C(=[C]2)O)[C][C]=C([C])[C])O)C(=O)[C]([C]1C1=[C]C(=C(C(=[C]1)O[C])O)O)O	-9
**58089059**	o1c(c(c(=O)c2[C]=C([C]=[C]c12)NC(=O)[C][C]C(=O)O)O)C1=[C][C]=C([C]=[C]1)O	-9
**54450648**	O1C2=[C][C]=[C][C]=C2C(=O)/C(=[C]\NC2=C([C]=[C][C]=[C]2)O)/[C]1C1=[C][C]=[C][C]=[C]1	-9
**53904139**	C1(=[C][C]=[C]C(=[C]1)C1=[C]C(=O)C2=C([C]=[C][C]=[C]2)O1)S(=O)(=O)N	-9
**49788613**	o1c(c(c(=O)c2=C([C]=C(C(=c12)O[C])O[C][C]=C([C])[C])O)O)C1=[C]C2=C([C]=[C]1)O[C]O2	-9
**46850208**	C1(=C([C]=C2C(=C1O)C(=O)[C]=C(O2)C1=[C]C(=C([C]=[C]1)O)O[C])O)[C]/[C]=C(\[C])/[C][C][C](C([C])([C])O)O	-9
**44257869**	O1C2=[C]C3=C(C(=O)[C]=C(O3)C3=[C][C]=C([C]=[C]3)O)C(=C2[C][C](C1([C])[C])O)O	-9
**42607867**	C1(=[C]C2=C(C(=[C]1)O)C(=O)[C][C](O2)C1=[C][C]=[C][C]=[C]1)O[C][C]1OC1([C])[C]	-9
**19358579**	O1C(=[C]C(=O)C2=C1[C]=C([C]=C2O[C])O[C]C1=[C][C]=[C][C]=[C]1)C1=[C][C]=C(C(=[C]1)O[C])O[C]	-9
**15389725**	C1(=[C]C(=O)C2=[C][C]=[C][C]=C2O1)C1=[C][C]=[C]C(=[C]1)S(=O)(=O)Cl	-9
**14735885**	C1(=[C]C(=O)C2=[C][C]=[C][C]=C2O1)C1=[C][C]=C([C]=[C]1)C(F)(F)F	-9
**12753176**	[C]1(C(=[C])C(=O)C2=[C][C]=[C][C]=C2O1)C1=C(Cl)[C]=C([C]=[C]1)Cl	-9
**12043064**	C1(=[C]C(=O)C2=C([C]=C(C(=C2O1)[C]1[C]N([C])[C][C][C]1O)O)O)C1=[C][C]=[C][C]=C1Cl	-9
**12043063**	C1(=[C]C(=O)C2=C([C]=C(C(=C2O1)[C]1[C]N([C])[C][C][C]1O)O[C])O[C])C1=[C][C]=[C][C]=C1Cl	-9
**10814209**	C1(=C([C]=c2c(=C1O)c(=O)c(c(o2)C1=[C][C]=C([C]=[C]1)O[C])O)O)OS([O])([O])C1=[C][C]=C([C]=[C]1)[C]	-9
**5320053**	O1[C]=C(C(=O)C2=[C][C]=C([C]=C12)O)C1=[C][C]=C(C(=[C]1)[C][C]=C([C])[C])O	-9
**3870517**	O1C2=C(C(=[C]C(=[C]2)OC(=O)[C])O)C(=O)[C][C]1C1=[C][C]=C([C]=[C]1)OC(=O)[C]	-9
**3070243**	c1(c([C])c(=O)c2[C]=[C]C(=C(c2o1)[C]N1[C][C][C][C][C]1)O[C])C1=[C][C]=C(F)[C]=[C]1	-9
**3070242**	c1(c([C])c(=O)c2[C]=[C]C(=C(c2o1)[C]N1[C][C][C][C]1)O[C])C1=[C][C]=C(F)[C]=[C]1	-9
**491719**	O1c2c(c(c([C])c(c2C(=O)[C][C]1C1=[C][C]=C([C]=[C]1)O)O)O)[C][C]=C([C])[C]	-9
**480763**	O1C2=[C]C(=C(C(=C2C(=O)[C][C]1C1=[C][C]=[C][C]=[C]1)O)[C][C]=C([C])[C])O	-9
**399491**	O1C(=[C]C(=O)C2=C1[C]=C(C(=C2O)[C][C]=C([C])[C])O)C1=C([C]=C([C]=[C]1)O)O	-9
**2361**	O1C(=[C]C(=O)C2=C1[C]=[C]C1=C2[C]=[C][C]=[C]1)C1=[C][C]=[C][C]=[C]1	-9
**145945693**	O1[C]([C]([C]([C]([C@]1(c1c(=O)c2=[C][C]=[C][C]=c2oc1C1=[C][C]=[C][C]=[C]1)O)O)O)O)[C]O	-8.9
**129881487**	O1C2=[C][C]=C([C]=C2C(=O)[C]=C1C1=[C][C]=[C][C]=C1C(=O)O)C(=O)O	-8.9

**129834539**	O1C2=C(C(=[C]C(=[C]2)O[C]C2=[C][C]=[C][C]=[C]2)O[C])C(=O)[C][C]1C1=[C][C]=C([C]=[C]1)O	-8.9
**129714302**	O1C2=[C][C]=[C][C]=C2C(=O)[C]([C@@]1(C1=[C][C]=[C][C]=[C]1)[C]C1=[C][C]=[C][C]=[C]1)O	-8.9
**102321611**	O1[C]=C(C(=O)C2=C1[C]=C([C]=C2O)C1=[C][C]2C(=[C][C]=[C]O2)C(=[C]1)O)C1=[C][C]=C(C(=C1O[C])O[C])O	-8.9
**101932121**	C1(=C(C(=C2C(=[C]1)OC(=[C]C2=O)C1=[C][C]=C([C]=[C]1)O)O)O[C])O[C]1O[C]([C]([C]([C]1O)O)O)[C]	-8.9
**101887718**	C1(=C([C]=C(C2=C1OC(=[C]C2=O)C1=[C][C]=C([C]=[C]1)O)O)O)[C]1O[C]([C])[C]([C]([C]1)O)O	-8.9
**101367767**	O1C2=C(C(=[C]C(=C2C(=O)[C][C]1C1=[C][C]=C([C]=[C]1)O)O)O)[C]/[C]=C(\[C])/[C]=O	-8.9
**101064055**	C1(=[C]OC2=[C][C]=[C][C]=C2C1=O)C1=[C][C]=C([C]=[C]1)C(F)(F)F	-8.9
**101028402**	O1C(=[C]C(=O)C2=C1[C]=C(C(=C2O)N[C][C]([C])[C])O)C1=[C][C]=[C][C]=[C]1	-8.9
**100941749**	O1C2=[C]C(=C(C(=C2C(=O)[C][C]1C1=[C]C(=C([C]=[C]1)O[C])O)O)C1=[C][C]=[C][C]=[C]1)O	-8.9
**71588424**	O1C2=[C]C(=O)[C]3C(=O)[C]=C(O[C]3C2=C([C](C1([C])[C])O)O)C1=[C][C]=C([C]=[C]1)O	-8.9
**66783913**	O1C(=[C]C(=O)C2=C(C3=C([C]=C12)O[C]O3)O)C1=[C][C]=C([C]=[C]1)O[C]	-8.9
**66728267**	C1(=[C]C(=[C][C]=C1O)C1=[C]OC2=[C]C(=[C]C(=C2C1=O)O)O)[C][C]C([C])([C])O	-8.9
**54505099**	O1C2=C([C]=[C][C]=[C]2)C(=O)/C(=[C]\N2[C]=N[C]=[C]2)/[C]1C1=[C][C]=[C][C]=[C]1	-8.9
**49788614**	o1c2[C]=C([C]=C(c2c(=O)c(c1C1=[C]C2=C([C]=[C]1)O[C]O2)O[C][C]=C([C])[C])O)O[C]	-8.9
**44578052**	O1C(=[C]C(=O)c2c(c(c(c(c12)[C]N1[C][C][C][C][C]1)O)O)O)C1=[C][C]=[C][C]=[C]1	-8.9
**44265534**	C1(=[C]C(=O)C2=C(C(=C([C]=C2O)O)C2=[C][C]N([C])[C][C]2)O1)C1=C(F)[C]=[C][C]=[C]1	-8.9
**44259326**	O1C2=[C]C(=c3c(=O)c(c(oc3=C2[C]([C])[C]C1([C])[C])C1=[C]C(=C([C]=[C]1)O)O)O)O)O	-8.9
**44258305**	C1(=[C]C(=C2C(=O)[C]=C(OC2=[C]1)C1=[C][C]=C([C]=[C]1)O)O[C])O[C][C]1OC1([C])[C]	-8.9
**42607940**	c1(c2c([C]=[C]C([C])([C])O2)c(c2C(=O)[C][C](Oc12)C1=[C][C]=C([C]=[C]1)O)O)[C]([C]1OC1([C])[C])O	-8.9
**24205532**	C1(=[C]c2c(C(=[C]1)O[C])c(=O)c(c(o2)C1=[C][C]=C([C]=[C]1)O)[C][C]=C([C])[C])O[C]1O[C]([C]([C]([C]1O)O)O)[C]	-8.9
**21315742**	C1(=[C]C(=O)C2=C([C]=[C]C(=[C]2)C(=O)O)O1)C1=[C]C(=[C][C]=C1O[C]([C])[C])[C]	-8.9

**15160702**	O1C2=C([C]=[C]C3=C2[C]=[C]C(O3)([C])[C])C(=O)[C][C]1C1=[C][C]=C(C(=[C]1)O[C])O[C]	-8.9
**14237660**	C1(=[C]OC2=C(C(=[C]C(=[C]2)O)O)C1=O)C1=[C][C]=C2OC([C]=[C]C2=[C]1)([C])[C]	-8.9
**12918915**	C1(=[C][C]=C2C(=O)C(=[C]OC2=[C]1)C1=[C][C]=[C][C]=[C]1)O[C](C1=[C][C]=[C][C]=[C]1)[C][C]N[C]	-8.9
**12093281**	O1C2=[C][C]=[C][C]=C2C(=O)/C(=[C]\C2=[C][C]=C([C]=[C]2)N(=O)=O)/[C]1C1=[C][C]=[C][C]=[C]1	-8.9
**11639597**	C1(=[C][C]=C2C(=O)[C]=C(OC2=[C]1)C1=[C][C]=C(C(=[C]1)O)O)O[C][C](O)[C]N[C]([C])[C]	-8.9
**11024735**	O1C2=C([C]=[C][C]=[C]2)C(=O)/C(=[C]/C2=[C][C]=C([C]=[C]2)N(=O)=O)/[C]1C1=[C][C]=[C][C]=[C]1	-8.9
**10472357**	O1C(=[C]C(=O)C2=C(C(=C([C]=C12)O)N[C][C][C]([C])[C])O)C1=[C][C]=[C][C]=[C]1	-8.9
**10337211**	O1C2=[C]C(=C([C]=C2C(=O)[C][C]1C1=[C][C]=C([C]=[C]1)O)[C][C]=C([C])[C])O[C]	-8.9
**9951997**	C1(=[C]C(=O)C2=C([C]=C(C(=C2O1)C1=[C][C]N([C])[C][C]1)O)O)C1=[C][C]=[C][C]=C1Cl	-8.9
**7330537**	O1C2=[C][C]=C3C(=O)C(=[C]OC3=C2[C]=[C]C1([C])[C])C1=[C]C2=C(C(=[C]1)O[C])O[C]O2	-8.9
**5317765**	c1(c(c(=O)c2=C([C]=C([C]=c2o1)O)O)O)C1=[C]C2=C(OC([C]([C]2)O)([C])[C])[C]=[C]1	-8.9
**5281814**	O1[C]=C(C(=O)C2=C1[C]=C(C(=C2O)[C][C]=C([C])[C])O)C1=[C][C]=C([C]=[C]1)O	-8.9
**509245**	O1C2=C(C(=[C]C(=C2C(=O)[C][C]1C1=[C][C]=C([C]=[C]1)O)O)O)[C][C]=C([C])[C]	-8.9
**343080**	O1[C]=C(C(=O)C2=C1[C]=C([C]=[C]2)O[C][C]=C([C])[C])C1=C([C]=C2C(=[C]1)O[C]O2)O[C]	-8.9
**129224**	C1(=[C]C2=C([C]=[C]1)C(=O)[C]=C(O2)C1=[C][C]=C([C])[C]=[C]1)O[C][C](O)[C]N[C]([C])[C]	-8.9
**122835**	O1C2=[C]C(=C([C]=C2C(=O)[C][C]1C1=[C][C]=C([C]=[C]1)O)[C][C]=C([C])[C])O[C]	-8.9
**136023343**	C1(=[C]C(=O)C2=C([C]=C(C(=C2O1)C1=[C][C]=N[C]=[C]1)O)O)C1=[C][C]=[C][C]=C1Cl	-8.8
**132540297**	o1c2[C]=C([C]=C(c2c(=O)c(c1C1=[C][C]=C(C(=[C]1)OC(=O)[C])OC(=O)[C])O)O)O	-8.8
**102066918**	o1c(c(c(=O)c2=C(C(=C([C]=c12)O[C]C1=[C][C]=[C][C]=[C]1)O[C])O)O)C1=[C][C]=[C][C]=[C]1	-8.8
**102005377**	C1(=[C]C(=O)C2=C([C]=C(C(=C2O1)C1=[C][C]N([C])[C][C]1)O[C])O[C])C1=[C][C]=[C]C(=[C]1)Cl	-8.8
**101843485**	C1(=[C]C2=C([C]=[C]1)O[C]([C]C2=O)C1=[C][C]=[C][C]=[C]1)O[C]1O[C]([C]([C]([C]1O)O)O[C])[C]O	-8.8
**101214938**	C1(=[C][C]=C([C]=[C]1)N1[C][C]N([C][C]1)C1=[C][C]=N[C]=[C]1)c1oc2=[C][C]=[C][C]=c2c(=O)c1O	-8.8
**91724402**	O1C2=[C]C(=[C]C(=C2C(=O)[C][C]1C1=[C][C]=C([C]=[C]1)OC(=O)[C])O)O[C]	-8.8
**71588336**	O1C2=[C]C(=C3C(=O)[C]=C(OC3=C2[C]([C](C1([C])[C])O)O)C1=[C][C]=C([C]=[C]1)O)O	-8.8

**57253387**	O1C2=C([C]=[C][C]=[C]2)[C]([C][C]1C1=[C][C]=[C][C]=[C]1)[N]NC(=O)N	-8.8
**56648913**	C1(=C(C(=C2C(=[C]1)OC(=[C]C2=O)C1=[C][C]=[C][C]=[C]1)OC(=O)[C])OC(=O)[C])O[C]C1=[C][C]=C(F)[C]=[C]1	-8.8
**54488609**	c1(c(=O)c2[C]=[C][C]=[C]c2oc1C1=[C][C]=[C][C]=[C]1)O[C]1O[C]([C]([C]([C]1O)O)O)[C]O	-8.8
**53247947**	C1(=[C][C]=C([C]=[C]1)[C]1[C]C(=O)C2=C([C]=[C][C]=[C]2)O1)O[C]1O[C]([C]([C]([C]1O)O)O)[C]O	-8.8
**46173806**	c1(c(=O)c2[C]=[C][C]=[C]c2oc1C1=[C][C]=[C][C]=[C]1)O[C]1O[C]([C]([C]([C]1O)O)O)[C]O	-8.8
**44258651**	O1C(=[C]C(=O)c2c(c(c(c(c12)O[C])O)O[C])O)C1=[C]C2=C([C]=[C]1)O[C]O2	-8.8
**42608112**	O1c2c(c(c(c(c2C(=O)[C][C]1C1=[C][C]=[C][C]=[C]1)O)[C]C1=[C]C(=[C][C]=C1O)O[C])O[C])O[C]	-8.8
**42608036**	O1[C]([C]C(=O)C2=C1C(=C([C]=C2O)O)[C][C]([C]([C])[C]O)[C][C]=C([C])[C])C1=[C][C]=C([C]=C1O[C])O	-8.8
**42607957**	O1C2=C(C(=[C]C(=C2C(=O)[C][C]1C1=[C][C]=C([C]=[C]1)O[C])O)O)[C]C(=C([C])[C])O	-8.8
**42607939**	O1C([C])([C])[C]=[C]c2c(c3C(=O)[C][C](Oc3c(c12)[C][C](C(=[C])[C])O)C1=[C][C]=C([C]=[C]1)O)O	-8.8
**23724473**	c1(c(=O)c2[C]=[C][C]=[C]c2oc1C1=[C][C]=[C][C]=[C]1)O[C]1O[C]([C]([C]([C]1O)O)O)[C]O	-8.8
**21604819**	O1C2=C(C(=[C][C]=C2[C][C][C]1C1=[C][C]=C([C]=[C]1)O)O)[C][C]=C([C])[C]	-8.8
**20301090**	O1C2=C([C]=[C][C]=[C]2)C(=O)C(=[C])[C]1C1=[C][C]=C([C]=[C]1)N(=O)=O	-8.8
**16126803**	c1(c(=O)c2[C]=[C][C]=[C]c2oc1C1=[C][C]=[C][C]=[C]1)O[C]1O[C]([C]([C]([C]1O)O)O)[C]O	-8.8
**16072086**	C1(=[C]C(=O)C2=C([C]=C(C(=C2O1)C1=[C][C]N([C])[C][C]1)O)O)C1=[C][C]=[C]C(=[C]1)Cl	-8.8
**12820520**	O1C2=C([C]=c3c(=O)c(c(oc3=C2[C]=[C]C1([C])[C])C1=[C]C2=C([C]=[C]1)O[C]O2)O[C])O[C]	-8.8
**12428924**	O1C2=[C][C]=[C][C]=C2C(=O)/C(=[C]\C2=[C][C]=C([C]=[C]2)O)/[C]1C1=[C][C]=[C][C]=[C]1	-8.8
**12146121**	O1C(=[C]C(=O)C2=C1[C]=C([C]=C2O[C][C]=C([C])[C])O[C]O[C])C1=[C][C]=[C][C]=[C]1	-8.8
**12043069**	C1(=[C]C(=O)C2=C(C(=C([C]=C2O)O)C2=[C]N=[C]N=[C]2)O1)C1=C(Cl)[C]=[C][C]=[C]1	-8.8
**11953828**	c1(c(=O)c2=[C][C]=[C][C]=c2oc1C1=[C][C]=[C][C]=[C]1)O[C]1O[C]([C]([C]([C]1O)O)O)[C]O	-8.8
**11552747**	C1(=[C][C]=C2C(=O)[C]=C(OC2=[C]1)C1=[C]C(=[C]C(=[C]1)O)O)O[C][C](O)[C]N[C]([C])[C]	-8.8
**11382659**	C1(=[C]C(=C(C(=[C]1)O)O[C])[C][C]=C([C])[C])[C]1OC2=[C]C(=[C][C]=C2C(=O)[C]1)O	-8.8
**11244593**	O1C2=[C]C(=C([C]=C2[C]=[C]C1([C])[C])C1=[C]OC2=C([C]=[C]C(=[C]2)O)C1=O)O	-8.8
**11111496**	O1C2=C(C(=[C]C(=C2C(=O)[C][C]1C1=[C][C]=C(C(=[C]1)O)O)O)O)[C]/[C]=C(\[C])/[C]O	-8.8
**10598514**	O1[C]([C]C(=O)C2=C1C(=C([C]=C2O)O)[C][C](C(=[C])[C])[C][C]C([C])([C])O)C1=C([C]=[C][C]=[C]1)O	-8.8
**10546844**	C1(=[C]OC2=C(C(=[C]C(=[C]2)O)O[C])C1=O)C1=[C]C(=C2OC([C]=[C]C2=[C]1)([C])[C])O	-8.8
**10543665**	C1(=[C]C(=O)C2=C([C]=[C][C]=C2O[C])O1)C1=[C]C(=[C][C]=[C]1)C(F)(F)F	-8.8
**10050660**	O1C2=C(C(=[C]C(=C2C(=O)[C][C]1C1=[C][C]=C([C]=[C]1)O[C])O[C])O[C])[C]1C(=O)C2=[C][C]=C([C]=C2O1)O[C]	-8.8
**5372237**	o1c2[C]=[C][C]=C(c2c(=O)c(c1C1=[C][C]=C([C]=[C]1)O[C])C(=O)C1=[C][C]=C([C]=[C]1)O[C])O	-8.8

**5317756**	O1C(=O)C(=[C]C2=C(C(=C([C]=C12)O)[C][C]=C([C])[C])O[C])C1=[C][C]=C([C]=C1O)O	-8.8
**5317478**	O1[C]=C(C(=O)C2=C(C(=C([C]=C12)O)[C][C]=C([C])[C])O)C1=[C][C]=C([C]=[C]1)O[C]	-8.8
**5315125**	O1C2=C(C(=[C]C(=[C]2)O)O)C(=O)[C]=C1C1=[C]C(=C(C(=[C]1)O)O)[C][C]=C([C])[C]	-8.8
**5281797**	O1[C]=C(C(=O)C2=C1[C]=C(C(=C2O)[C][C]=C([C])[C])O)C1=[C][C]=C([C]=C1O)O	-8.8
**5281789**	O1[C]=C(C(=O)C2=C1[C]=C([C]=C2O)O)C1=C(C(=C([C]=[C]1)O)[C][C]=C([C])[C])O	-8.8
**624794**	o1c2=[C][C]=[C][C]=c2c(=O)c(c1C1=[C][C]=C2C(=[C]1)O[C]O2)O	-8.8
**462700**	O1C2=[C][C]=[C][C]=C2C(=O)[C][C]1C1=[C]C(=C([C]=[C]1)O)O	-8.8
**343079**	O1[C]=C(C(=O)C2=C1C1=C([C]=[C]2)O[C]O1)C1=[C][C]=C2C(=[C]1)O[C]O2	-8.8
**227443**	o1c(c(c(=O)c2=[C]C(=[C][C]=c12)[C])O)C1=[C][C]=C([C]=[C]1)O[C]	-8.8
**49736**	o1c(c([C])c(=O)c2[C]=[C]C(=C(c12)[C]N1[C][C][C][C][C]1)O[C])C1=[C][C]=[C][C]=[C]1	-8.8
**145706427**	O1C2=C([C]=[C][C]=[C]2)C(=O)[C][C]1C1=[C]C(=[C]C(=[C]1)O)O[C]	-8.7
**139593878**	O1C(=[C]C(=O)C2=C1C(=[C]C(=[C]2)[C][C][C]O)O[C])C1=[C][C]=C([C]=[C]1)O[C]	-8.7
**133611769**	C1(=[C]C2=C([C]=[C]1)C(=O)C(=[C]O2)C1=[C]C(=[C]C(=[C]1)O[C])O)O[C]1O[C]([C]([C]([C]1O)O)O)[C]O	-8.7
**132533490**	O1[C]=C(C(=O)C2=C1[C]=C(C(=C2O)[C][C](C(=[C])[C])O)O)C1=[C][C]=C2C(=[C]1)O[C]O2	-8.7
**129821165**	C1(=C([C]=C2C(=C1O)C(=O)[C][C](O2)C1=[C][C]=[C][C]=[C]1)O[C])[C][C]1OC1([C])[C]	-8.7
**129716748**	[P@]12(=O)O[C]3[C]4C5=C([C]=[C][C]=[C]5)O[P@]5(=O)O[C]6[C]([C]([C]([C]([C]6O1)O2)C3=O)O4)O5	-8.7
**129684435**	O1C2=C([C]=[C][C]=[C]2)[C][C]([C]1C1=[C][C]=[C][C]=[C]1)O	-8.7
**122383344**	O1C2=[C]C(=C([C]=C2C(=O)[C][C]1C1=[C][C]=C([C]=[C]1)O)[C][C](C(=[C])[C])O)O	-8.7
**102341487**	C1(=[C]C(=C2C(=O)[C]=C(OC2=[C]1)C1=[C][C]=C(C(=[C]1)O[C])O[C])O)O[C]1O[C]([C]([C]([C]1O)O)O)[C]	-8.7
**102307255**	c1(c(=O)c2[C]=[C][C]=[C]c2oc1C1=[C][C]=[C][C]=[C]1)C(F)(F)F	-8.7

**102005380**	C1(=[C]C(=O)C2=C(C(=C([C]=C2O[C])O[C])C2=[C][C]N([C])[C][C]2)O1)C1=C(Br)[C]=[C][C]=[C]1	-8.7
**102005373**	C1(=[C]C(=O)C2=C(C(=C([C]=C2O[C])O[C])[C]2[C]N([C])[C][C][C]2O)O1)C1=C(Cl)[C]=[C][C]=[C]1	-8.7
**101932173**	C1(=C(C(=C2C(=O)[C][C](OC2=[C]1)C1=[C][C]=[C][C]=[C]1)O)O[C])O[C]1O[C]([C]([C]([C]1O)O)O)[C]O	-8.7
**101843486**	C1(=[C]C2=C([C]=[C]1)O[C]([C]C2=O)C1=[C][C]=[C][C]=[C]1)O[C]1O[C]([C]([C]([C]1O)O)O[C])[C]O	-8.7
**90300755**	o1c(c(c(=O)c2[C]=[C][C]=C(c12)[C][C]=C([C])[C])O)C1=[C][C]=[C][C]=[C]1	-8.7
**71588337**	O1C2=C(C3=C(C(=[C]2)O)C(=O)[C]=C(O3)C2=[C][C]=C([C]=[C]2)O)[C]([C](C1([C])[C])O)O[C][C]	-8.7
**67424417**	C1(=[C][C]=C([C]=[C]1)[C]1[C]C(=O)C2=C([C]=[C][C]=[C]2)O1)S(=O)(=O)C(F)(F)F	-8.7
**56649091**	C1(=[C]C2=C(C(=C1O)O)C(=O)[C]=C(O2)C1=[C][C]=[C][C]=[C]1)O[C][C]N1[C][C][C][C][C]1	-8.7
**56649090**	C1(=C(C(=C2C(=O)[C]=C(OC2=[C]1)C1=[C][C]=[C][C]=[C]1)O)O)O[C][C]N1[C][C]O[C][C]1	-8.7
**44481784**	O1[C]=C(C(=O)C2=C([C]=C([C]=C12)O[C])O)C1=[C]C(=C([C]=[C]1)O)[C][C](C(=[C])[C])O	-8.7
**44342639**	C1(=C([C]=C(C2=C1OC(=[C]C2=O)C1=[C][C]=[C][C]=[C]1)O)O[C])C(F)(F)F	-8.7
**44265521**	C1(=[C]C(=O)C2=C([C]=C(C(=C2O1)C1=[C][C]N([C])[C][C]1)O)O)C1=[C][C]=C([C]=C1Cl)Cl	-8.7
**44259050**	O1C2=[C]c3c(c(=O)c(c(o3)C3=[C][C]=C([C]=[C]3)O)O)C(=C2[C]=[C]C1([C])[C])O	-8.7
**42607959**	C1(=[C]C(=C(C(=[C]1)[C][C]=C([C])[C])O[C])/[C]=[C]/C([C])([C])O)[C]1OC2=[C]C(=[C]C(=C2C(=O)[C]1)O)O	-8.7
**42607870**	O1C2=C(C(=[C]C(=C2C(=O)[C][C]1C1=[C][C]=[C][C]=[C]1)O)O)[C]/[C]=C(\[C])/[C]=O	-8.7
**25067674**	C1(=[C]C(=O)C2=C([C]=C(C(=C2O1)[C]1[C][C]N([C])[C][C]1O)O[C])O[C])C1=[C][C]=[C][C]=C1Cl	-8.7
**22737159**	O1C2=[C][C]=[C][C]=C2[C][C]([C]1C1=[C][C]=[C][C]=[C]1)OC(=O)C1=[C]C(=C(C(=[C]1)O)O)O	-8.7
**21325023**	O1C2=C([C]=[C]C(=[C]2)C(=O)O)C(=O)[C]=C1C1=C([C]=[C][C]=[C]1)O[C]([C])[C]	-8.7
**21325016**	C1(=[C]C(=O)C2=[C]C(=[C][C]=C2O1)C(=O)O)C1=C([C]=[C][C]=[C]1)O[C][C]([C][C])O	-8.7
**15389730**	C1(=[C][C]=[C]C(=[C]1)C1=[C]C(=O)C2=C([C]=[C][C]=[C]2)O1)S(=O)(=O)N([C][C])[C][C]	-8.7
**14033976**	O1C2=C(C3=C(C(=[C]2)O[C])C(=O)[C]=C(O3)C2=[C][C]=C3C(=[C]2)O[C]O3)[C]=[C]C1([C])[C]	-8.7
**12753167**	[C]1(C(=[C])C(=O)C2=C([C]=[C][C]=[C]2)O1)C1=[C][C]=C(Cl)[C]=[C]1	-8.7
**12318031**	O1C2=C([C]=[C][C]=[C]2)[C][C]([C]1C1=[C][C]=[C][C]=[C]1)O	-8.7
**12052874**	O1C2=C([C]=[C]C(=[C]2)O)[C][C][C]1C1=[C]C2=C([C]=[C]1)O[C]O2	-8.7
**12043065**	C1(=[C]C(=O)C2=C([C]=C(C(=C2O1)[C]1[C]N([C])[C][C][C]1O)O)O)C1=[C][C]=[C][C]=C1Cl	-8.7

**11810419**	O1C2=C(C(=[C]C(=C2C(=O)[C][C]1C1=[C][C]=C([C]=C1O)O)O)O)[C][C]=C([C])[C]	-8.7
**11723752**	O1[C]=C(C(=O)C2=C1[C]=C([C]=C2[C])OC(=O)O[C][C])C1=[C][C]=[C][C]=[C]1	-8.7
**11711336**	c1(c(=O)c2=[C][C]=[C][C]=c2oc1C1=[C][C]=[C][C]=[C]1)O[C]1O[C]([C]([C]([C]1O)O)O[C])[C]O	-8.7
**11611332**	C1(=[C]C2=C([C]=[C]1)C(=O)[C]=C(O2)C1=[C][C]=C(C(=[C]1)O[C])O[C])O[C][C](O)[C]N[C]([C])[C]	-8.7
**11507421**	O1C2=[C][C]=[C][C]=C2C(=O)[C]=C1C1=[C][C]=C([C]=[C]1)OC(=O)[C]	-8.7
**11177340**	O1C(=[C]C(=O)C2=C1[C]=C([C]=C2O[C][C]=C([C])[C])O[C]O[C][C]O[C])C1=[C][C]=[C][C]=[C]1	-8.7
**11164320**	c1(c(c(=O)c2[C]=[C]C(=[C]c2o1)O)C1=[C][C]=[C][C]=[C]1)S[C]C1=[C][C]=N[C]=[C]1	-8.7
**10889522**	O=C1C2=C([C]=[C][C]=[C]2)N[C]([C]1)C1=[C][C]=[C][C]=[C]1	-8.7
**10832934**	O1C2=[C][C]=C([C]=C2[C]([C](C1([C])[C])O)O)C1=[C]OC2=C(C(=[C]C(=[C]2)O)O)C1=O	-8.7
**10317448**	c1(c(c(=O)c2[C]=[C]C(=[C]c2o1)O)C1=[C][C]=C([C]=[C]1)O)S[C]C1=[C][C]=N[C]=[C]1	-8.7
**9948081**	C1(=[C]C2=C([C]=[C]1)C(=O)[C]=C(O2)C1=[C][C]=[C][C]=[C]1)O[C][C]1O[C]1	-8.7
**5469928**	O1C(=[C]C(=O)C2=C1C(=C([C]=C2O)O)[C]1[C][C][C][C]N1)C1=[C][C]=[C][C]=[C]1	-8.7
**5326329**	O1[C]=C(C(=O)C2=[C][C]=C(C(=C12)[C][C]=C([C])[C])O)C1=[C]C(=C([C]=[C]1)O[C])O	-8.7
**3707243**	O1C2=C([C]=[C][C]=[C]2)[C][C]([C]1C1=[C][C]=[C][C]=[C]1)O	-8.7
**3534982**	O1C2=C([C]=[C][C]=[C]2)C(=O)[C][C]1C1=[C]C(=[C][C]=[C]1)O	-8.7
**3070247**	c1(c([C])c(=O)c2[C]=[C]C(=C(c2o1)[C]N1[C][C][C][C]1)O[C])C1=[C][C]=C(Cl)[C]=[C]1	-8.7
**3033107**	C1(=[C][C]=C([C]=[C]1)c1c([C])c(=O)c2=[C][C]=[C][C]=c2o1)O[C][C](O)[C]NC([C])([C])[C]	-8.7
**688861**	O1C2=C([C]=[C][C]=[C]2)C(=O)[C][C]1C1=[C]C(=[C][C]=[C]1)O	-8.7
**637879**	O1[C]=C(C(=O)C2=C(C3=C([C]=C12)O[C]O3)O[C])C1=[C][C]=C2C(=[C]1)O[C]O2	-8.7
**635396**	C1(=C([C]=C(C2=C1OC(=[C]C2=O)C1=[C][C]=C([C]=[C]1)O[C])O[C])O[C])[C]1O[C]([C]([C]([C]1O)O)O)[C]O	-8.7
**625631**	O1C(=[C]C(=O)C2=C1[C]=[C]C1=C2[C]=[C]C(=[C]1)O)C1=[C][C]=[C][C]=[C]1	-8.7
**466078**	O1C2=C([C]=[C]C(=[C]2)O)[C][C][C]1C1=[C]C2=C([C]=[C]1)O[C]O2	-8.7
**54410**	C1(=[C]C2=C([C]=[C]1)C(=O)[C]=C(O2)C1=[C][C]=[C][C]=[C]1)O[C][C](O)[C]N[C][C][C]	-8.7
**41450**	C1(=[C][C]=C([C]=[C]1)C1=[C]C(=O)C2=[C][C]=[C][C]=C2O1)O[C][C](O)[C]NC([C])([C])[C]	-8.7
**11834**	C1(=[C]C2=C([C]=[C]1)C(=O)[C]=C(O2)C1=[C][C]=[C][C]=[C]1)O[C][C]([C])[C]N([C])[C]	-8.7
**139584124**	O1[C]=C(C(=O)C2=C([C]=C([C]=C12)O[C])O)C1=[C][C]=C([C]=[C]1)O[C][C]=C([C])[C]	-8.6
**129881940**	c1(c(=O)c2=[C][C]=[C][C]=c2oc1C1=[C][C]=[C][C]=[C]1)[C]1O[C]([C])[C]([C]([C]1)O)O	-8.6

**129824498**	O1C(=[C]C(=O)C2=C1C(=C([C]=C2O[C])O[C])C1=[C][C]=C(C(=[C]1)O[C])O[C])C1=[C][C]=[C][C]=[C]1	-8.6
**129675099**	o1c2=[C][C]=[C][C]=c2c(=O)c(c1C1=[C][C]=[C][C]=[C]1)OC(=O)C1=[C]C(=C(C(=[C]1)O)O)O	-8.6
**102369763**	O1C2=[C][C]=C3[C][C][C](OC3=C2[C][C]1C([C])([C])O)C1=[C][C]=C([C]=[C]1)O	-8.6
**102005378**	C1(=[C]C(=O)C2=C(C(=C([C]=C2O[C])O[C])C2=[C][C]N([C])[C][C]2)O1)C1=[C][C]=C(Cl)[C]=[C]1	-8.6
**100943382**	ClC1=[C]C(=[C]C(=C1O[C])S(=O)(=O)N[C]([C])[C])C1=[C]C(=O)C2=C([C]=[C][C]=[C]2)O1	-8.6
**91557562**	O1C2=C([C]=[C]C(=[C]2)O)C(=O)[C][C]1C1=[C][C]=C([C]=C1O)O	-8.6
**76852307**	O1C(=[C]C(=O)C2=C1C(=C([C]=[C]2)O)[C]=O)C1=[C][C]=[C][C]=[C]1	-8.6
**74819425**	O1C2=[C]C3=C(C(=C2C(=O)[C][C]1C1=C([C]=[C][C]=[C]1)O)O)O[C]O3	-8.6
**59059924**	O1[C]=C(C(=O)C2=[C][C]=C([C]=C12)OC(=O)C(=[C])[C])C1=[C][C]=[C][C]=[C]1	-8.6
**57679080**	O1C2=[C][C]=[C][C]=C2[C][C][C]1C1=[C][C]=C(C(=[C]1)O)O	-8.6
**53947837**	O1C2=[C][C]=C([C])[C]=C2[C][C][C]1C1=[C][C]=C([C]=[C]1)O[C]	-8.6
**50909808**	O1[C]=C(C(=O)C2=C([C]=C([C]=C12)O)[O])C1=[C][C]=C2C(=[C]1)O[C]O2	-8.6
**44259047**	C1(=C(c2c([C]=C1O)oc(c(c2=O)O)C1=[C][C]=C([C]=[C]1)O)O)[C][C]C([C])([C])O	-8.6
**42607933**	O1C2=C(C(=[C]C(=C2C(=O)[C][C]1C1=[C][C]=C([C]=[C]1)O)O)O)[C][C](C(=[C])[C])O	-8.6
**25128870**	O1C(=[C]C(=O)C2=C1[C]=C([C]=C2O[C])O[C]C1=N[N]N[N]1)C1=[C][C]=C(C(=[C]1)O[C])O[C]	-8.6
**23286063**	o1c(c(c(=O)c2=[C]C(=[C][C]=c12)C(=O)O)O)C1=[C][C]=[C][C]=[C]1	-8.6
**18324937**	c1(c(c(=O)c2=[C][C]=[C][C]=c2o1)C1=[C][C]=C([C]=[C]1)F)C1=[C][C]=C([C]=[C]1)S(=O)(=O)N	-8.6
**15731439**	O1C(=[C]C(=O)C2=[C][C]=C(C(=C12)OC(=O)[C])OC(=O)[C])C1=[C][C]=[C][C]=[C]1	-8.6
**15389726**	C1(=[C]C(=O)C2=[C][C]=[C][C]=C2O1)C1=[C][C]=C([C]=[C]1)S(=O)(=O)Cl	-8.6
**14630497**	o1c(c(c(=O)c2=C([C]=C3C(=c12)[C]=[C]C([C])([C])O3)O)[C][C]C([C])([C])O)C1=C([C]=C(C(=[C]1)O)O)O	-8.6
**14502737**	O1C2=C(C3=C(C(=[C]2)O)C(=O)C(=[C]O3)C2=[C][C]=C3C(=[C]2)O[C]O3)[C]=[C]C1([C])[C]	-8.6
**14134114**	o1c(c(c(=O)c2C(=[C]C(=C(c12)C(=O)[C][C]([C])C(=O)O)O)O)O)C1=[C][C]=C([C]=[C]1)O	-8.6
**14130926**	o1c(c(c(=O)c2=C([C]=C([C]=c12)OC(=O)[C])OC(=O)[C])OC(=O)[C])C1=[C][C]=C([C]=[C]1)OC(=O)[C]	-8.6
**14016776**	C1(=[C]C(=C2C(=O)[C]=C(OC2=[C]1)C1=[C][C]=C([C]=[C]1)O)O)OS([O])([O])O	-8.6

**12988090**	C1(=[C]C2=C([C]=[C]1)C(=O)[C]=C(O2)C1=[C][C]=[C][C]=[C]1)OP(=O)(O[C][C])O[C][C]	-8.6
**11954210**	C1(=[C][C]=c2c(=O)c(c(oc2=[C]1)C1=[C][C]=[C][C]=[C]1)O)O[C]1O[C]([C]([C]([C]1O)O)O)[C]O	-8.6
**11725803**	O1C2=C(C(=[C]C(=[C]2)O)O)C(=O)[C][C]1C1=[C]C(=C(C(=[C]1)O)O)[C][C]=C([C])[C]	-8.6
**11612258**	C1(=[C]C2=C([C]=[C]1)C(=O)[C]=C(O2)C1=[C]C(=C(C(=[C]1)O[C])O[C])O[C])O[C][C](O)[C]NC([C])([C])[C]	-8.6
**10708855**	C1(=[C]C(=O)C2=C([C]=[C][C]=[C]2)O1)C1=[C]C(=[C][C]=[C]1)[C]N=C=S	-8.6
**10543327**	C1(=[C]C(=O)C2=[C][C]=[C][C]=C2O1)C1=[C][C]=[C]C(=[C]1)[C]Br	-8.6
**10450773**	O1C(=[C]C(=O)C2=C1[C]=C(C(=C2O)/[C]=[C]/[C]([C])[C])O)C1=C([C]=C([C]=[C]1)O)O	-8.6
**9973510**	O1C2=C([C]=C([C]=[C]2)C(=O)O)C(=O)[C]=C1C1=C([C]=[C][C]=[C]1)O[C]([C])[C]	-8.6
**9950264**	C1(=C(C2=C([C]=C1O)OC(=[C]C2=O)C1=[C][C]=[C][C]=[C]1)O)OS([O])([O])O	-8.6
**9928523**	O1C2=C(C(=[C]C(=C2C(=O)[C][C]1C1=[C][C]=C([C]=[C]1)O)O[C])O)[C][C]=C([C])[C]	-8.6
**5481229**	O1C2=C(C(=[C]C(=C2O)O[C])C2=[C]OC3=[C]C(=[C]C(=C3C2=O)O)O)[C]=[C]C1([C])[C]	-8.6
**5471244**	C1(=[C]C(=C2C(=O)[C]=C(OC2=[C]1)C1=[C][C]=[C][C]=[C]1)O)OS([O])([O])O	-8.6
**5318267**	C1(=[C][C]=C2C(=O)C(=[C]OC2=[C]1)C1=[C][C]=C(C(=[C]1)O)O[C])O[C]1O[C]([C]([C]([C]1O)O)O)[C]O	-8.6
**5270553**	O1C2=C([C]=C([C]=[C]2)C(=O)O)C(=O)[C][C]1C1=[C][C]=[C][C]=[C]1	-8.6
**3062797**	O1[C]=C(C(=O)C2=C1[C]=C([C]=[C]2)O[C]([C])C(=O)O)C1=[C][C]=[C][C]=[C]1	-8.6
**725376**	O1C(=[C]C(=O)C2=C1[C]=[C]C(=[C]2)OC(=O)[C])C1=[C][C]=C([C]=[C]1)O[C]	-8.6
**467492**	O1C(=[C]C(=O)C2=C1[C]=C([C]=C2OC(=O)[C])OC(=O)[C])C1=[C][C]=[C][C]=[C]1	-8.6
**466293**	ClC1=[C]c2c([C]=[C]1)oc(c(c2=O)O)C1=[C][C]=C([C]=[C]1)O[C]	-8.6
**462699**	O1C2=[C][C]=C([C]=C2C(=O)[C][C]1C1=[C][C]=C([C]=[C]1)O)O	-8.6
**368925**	O1C2=C(C(=[C]C(=C2[C][C]=C([C])[C])O)O)C(=O)[C]([C]1C1=[C][C]=C([C]=[C]1)O)O	-8.6
**227445**	o1c(c(c(=O)c2[C]=C([C])[C]=[C]c12)O)C1=[C][C]=[C][C]=[C]1	-8.6
**182342**	O1C2=[C][C]=C3C(=O)C(=[C]OC3=C2[C]=[C]C1([C])[C])C1=[C]C2=C([C]=[C]1)O[C]O2	-8.6
**118907**	O1C(=[C]C(=O)C2=C([C]=C([C]=C12)O[C]C(=O)O[C][C])O[C]C(=O)O[C][C])C1=[C][C]=[C][C]=[C]1	-8.6
**11912**	C1(=[C][C]=C2C(=O)[C]=C(OC2=[C]1)C1=[C][C]=[C][C]=[C]1)O[C][C][C]N([C])[C]	-8.6
**139080697**	o1c(c(c(=O)c2C(=[C]C(=[C]c12)O[C][C]=C([C])[C])O)O)C1=[C][C]=C(C(=[C]1)O)O[C]	-8.5
**134821268**	O1[C]2[C]C3=[C][C]=[C][C]=C3O[C@]12C1=[C][C]=[C][C]=[C]1	-8.5

**133064953**	o1c(c(c(=O)c2C(=[C][C]=[C]c12)[C]C#[C])N)C1=[C][C]=[C][C]=[C]1	-8.5
**132517366**	O1C(=[C]C(=O)C2=C1C(=C(C(=[C]2)O)O[C])/[C]=C(\[C])/[C]O)C1=[C][C]=C([C]=[C]1)O[C]	-8.5
**129834507**	O1C2=[C]C(=[C]C(=C2C(=O)[C][C]1C1=[C][C]=C([C]=[C]1)OC(=O)[C])O[C])OC(=O)[C]	-8.5
**129822395**	Clc1c(c(c2C(=O)[C]=C(Oc12)C1=[C][C]=C([C]=[C]1)OC(=O)[C])OC(=O)[C])OC(=O)[C])OC(=O)[C]	-8.5
**129822361**	O1C(=[C]C(=O)C2=C1C(=C(C(=[C]2)OC(=O)[C])OC(=O)[C])N)C1=[C][C]=[C][C]=[C]1	-8.5
**102577932**	C1(=[C]C2=C(C(=[C]1)O)C(=O)[C]=C(O2)C1=[C][C]=C([C]=[C]1)O)OS([C])([O])[O]	-8.5
**102005379**	C1(=[C]C(=O)C2=C(C(=C([C]=C2O[C])O[C])C2=[C][C]N([C])[C][C]2)O1)C1=[C][C]=[C][C]=C1F	-8.5
**102005376**	C1(=[C]C(=O)C2=C(C(=C([C]=C2O[C])O[C])C2=[C][C]N([C])[C][C]2)O1)C1=[C][C]=[C][C]=C1Cl	-8.5
**102003045**	O1C2=[C]C(=[C][C]C2=[C]C(=[C]1)C1=C([C]=C2C(=[C]1)O[C]O2)O[C])O	-8.5
**101680705**	C1(=[C]C2=C([C]=[C]1)C(=O)[C]=C(O2)C1=[C][C]=[C][C]=[C]1)C(=O)[C]Br	-8.5
**101643010**	O1C2=C(C(=[C]C(=C2[C][C]=C([C])[C])O)O)C(=O)[C]([C]1C1=[C][C]=C([C]=[C]1)O)O	-8.5
**101440521**	o1c2[C]=C([C]=C(c2c(=O)c(c1C1=[C][C]=C(C(=[C]1)[C][C](C(=[C])[C])O)O)O)O)O	-8.5
**101367766**	O1C2=C(C(=[C]C(=C2C(=O)[C][C]1C1=[C][C]=C([C]=[C]1)O)O)O)[C]/[C]=C(\[C])/[C]O	-8.5
**101213759**	O1[C]=C(C(=O)C2=C1[C]=C([C]=C2O)O)C1=C([C]=C(C(=[C]1)[C][C]=C([C])[C])O[C])O	-8.5
**101101153**	C1(=C(C(=C2C(=[C]1)OC(=[C]C2=O)C1=[C][C]=[C][C]=[C]1)O)O[C])O[C]1O[C]([C]([C]([C]1O)O)O)[C]O	-8.5
**90753092**	o1c(c(c(=O)c2=C([C])C(=C([C])[C]=c12)[C])O)C1=[C][C]=[C][C]=[C]1	-8.5

**69279473**	O1C2=C([C]=C([C]=[C]2)[C][C][C][C])C(=O)[C]([C]1C1=[C][C]=[C][C]=[C]1)O	-8.5
**54512651**	O1C2=[C][C]=[C][C]=C2C(=O)/C(=[C]\N)/[C]1C1=[C][C]=[C][C]=[C]1	-8.5
**53747690**	C1(=[C]C2=C(C(=[C]1)O)C(=O)[C]=C(O2)C1=[C][C]=[C][C]=[C]1)C(=O)[C]Br	-8.5
**53247945**	C1(=[C][C]=C([C]=[C]1)[C]1[C]C(=O)C2=C([C]=[C][C]=[C]2)O1)O[C]1O[C]([C]([C]([C]1O)O)O[C])[C]O	-8.5
**49800274**	O1C(=[C]C(=O)C2=C1C(=C([C]=C2O[C])O[C])C1=[C][C]N([C])[C][C]1)C1=[C][C]=[C][C]=[C]1	-8.5
**44382543**	C1(=C([C]=C(C2=C1OC(=[C]C2=O)C1=[C][C]=[C][C]=[C]1)O[C])O[C])C(F)(F)F	-8.5
**44380969**	O1C(=[C]C(=O)C2=C([C]=C(C(=C12)N[C][C][C]([C])[C])O)O)C1=[C][C]=[C][C]=[C]1	-8.5
**44380895**	O1C(=[C]C(=O)C2=C([C]=C(C(=C12)N[C][C]([C])[C])O)O)C1=[C][C]=[C][C]=[C]1	-8.5
**44265561**	C1(=[C]C(=O)C2=C(C(=C([C]=C2O)O)C2=[C][C]N([C])[C][C]2)O1)C1=[C][C]=C(Cl)[C]=[C]1	-8.5
**44265528**	C1(=[C]C(=O)C2=C([C]=C(C(=C2O1)C1=[C][C]N([C])[C][C]1)O)O)C1=[C][C]=[C][C]=C1Br	-8.5
**44265522**	O1C(=[C]C(=O)C2=C1C(=C([C]=C2O)O)C1=[C][C]N([C])[C][C]1)C1=[C][C]=[C][C]=[C]1	-8.5
**44258693**	O1C2=C(c3c([C]=C2O[C])c(=O)c(c(o3)C2=[C][C]=C([C]=[C]2)O)O[C])[C]=[C]C1([C])[C]	-8.5
**42607869**	O1C2=C(C(=[C]C(=C2C(=O)[C][C]1C1=[C][C]=[C][C]=[C]1)O)O)[C]/[C]=C(\[C])/[C]O	-8.5
**25201019**	O1C2=C(C(=[C]C(=[C]2)O)[O])C(=O)[C][C]1C1=[C][C]=C([C]=[C]1)O[C]	-8.5
**25058575**	C1(=C([C]=C(C(=[C]1)O[C])O[C][C]=C([C])[C])O[C])C1=[C]OC2=C(C1=O)C(=[C]C(=[C]2)O[C])O[C]	-8.5
**21325058**	O1C2=C([C]=C([C]=[C]2)C(=O)O[C])C(=O)[C][C]1C1=[C][C]=[C][C]=C1N(=O)=O	-8.5
**14791391**	O1C2=C([C]=[C]C(=[C]2)O)C(=O)[C]=C1C1=[C][C]=C([C]=[C]1)N(=O)=O	-8.5
**14033984**	O1C2=C([C]=c3c(=O)c(c(oc3=C2[C]=[C]C1([C])[C])C1=[C][C]=[C][C]=[C]1)O[C])O[C]	-8.5
**13432335**	O1C(=[C]C(=O)C2=C([C])[C]=C([C])[C]=C12)C1=[C][C]=[C][C]=[C]1	-8.5
**13393782**	[C]1([C]C(=O)C2=[C][C]=[C][C]=C2O1)C1=[C][C]=[C]C(=[C]1)F	-8.5
**12753173**	[C]1([C]C(=O)C2=[C][C]=[C][C]=C2O1)C1=C(Cl)[C]=C([C]=[C]1)Cl	-8.5
**12049385**	O1C(=[C][C]=C2[C][C]=C([C]=C12)N([C][C])[C][C])C1=[C][C]=C([C]=[C]1)N([C])[C]	-8.5
**12043066**	C1(=[C]C(=O)C2=C([C]=C(C(=C2O1)[C]1[C][C][C][C][C]1O)O)O)C1=[C][C]=[C][C]=C1Cl	-8.5
**11683340**	C1(=[C]C2=C([C]=[C]1)C(=O)[C]=C(O2)C1=[C]C(=[C]C(=[C]1)O[C])O[C])O[C][C](O)[C]NC([C])([C])[C]	-8.5
**11186717**	O1C2=C([C]=C([C]=C2[C]=[C]C1([C])[C])C1=[C]OC2=C([C]=[C]C(=[C]2)O)C1=O)O	-8.5
**10957930**	O1C2=[C][C]=[C][C]=C2C(=O)/C(=[C]/C2=[C][C]=[C][C]=[C]2)/[C]1C1=[C][C]=[C][C]=[C]1	-8.5
**10848398**	O1C2=C([C]=[C]C(=[C]2)O)C(=O)[C]=C1C1=[C][C]=C([C])[C]=[C]1	-8.5
**10761522**	[C]1([C]C(=O)C2=C([C]=C([C]=C2O)O)O1)C1=[C]C(=C2OC([C]=[C]C2=[C]1)([C])[C])O[C]	-8.5
**10708856**	C1(=[C]C(=O)C2=C([C]=[C][C]=[C]2)O1)C1=[C][C]=C([C]=[C]1)[C]N=C=S	-8.5

**10018499**	O1c2c(c(c([C])c(c2C(=O)[C][C@]1(C1=[C][C]=[C][C]=[C]1)O)O)O)[C]=O	-8.5
**6116651**	O1C2=[C][C]=[C][C]=C2C(=O)/C(=[C]\C2=[C][C]=N[C]=[C]2)/[C]1C1=[C][C]=[C][C]=[C]1	-8.5
**5378945**	O1[C]=C(C(=O)C2=C(C(=C([C]=C12)O)[C]/[C]=C(\[C])/[C]O)O)C1=[C][C]=C([C]=[C]1)O	-8.5
**5322065**	O1C2=C([C]=[C]C(=[C]2)O)C(=O)[C]=C1C1=[C]C(=C([C]=[C]1)O)O	-8.5
**5321987**	[C]1(C2=C([C]=[C]C(=[C]2)O[C])O[C][C]1C1=[C]C(=[C]C(=C1O)O[C])/[C]=[C]/C(=O)O)OS([O])([O])O	-8.5
**5317300**	C1(=C(C(=C([C]=[C]1)O)[C][C]=C([C])[C])O)C1=[C]OC2=C(C1=O)[C]=[C]C(=[C]2)O	-8.5
**5272799**	O1C2=[C][C]=[C][C]=C2C(=O)[C][C]1C1=[C][C]=C([C]=[C]1)C1=N[C][C]O1	-8.5
**5270543**	FC1=[C]C2=C([C]=[C]1)C(=O)[C][C](O2)C1=[C][C]=[C][C]=[C]1	-8.5
**5049148**	O1c2c(c(c3C(=O)C(=[C]Oc3c2[C]=[C]C1([C])[C])C1=[C]C2=C([C]=[C]1)O[C]O2)O[C])O[C]	-8.5
**746742**	C1(=[C]OC2=C([C]=[C]C(=[C]2)O[C]([C])[C])C1=O)C1=[C][C]=C(F)[C]=[C]1	-8.5
**630804**	O1[C]=C(C(=O)C2=C1C(=C([C]=C2OC(=O)[C])OC(=O)[C])[C][C]=C([C])[C])C1=[C][C]=C([C]=[C]1)OC(=O)[C]	-8.5
**342294**	O1C2=[C]C(=[C]C(=C2[C][C][C]1C1=[C][C]=[C][C]=[C]1)O[C])O	-8.5
**188424**	O1C2=C(C(=[C]C(=[C]2)O)O[C])C(=O)[C][C]1C1=[C][C]=C([C]=[C]1)O	-8.5
**187093**	O1C2=[C]C(=[C][C]=C2C(=O)[C][C]1C1=[C]C(=C([C]=[C]1)O[C])O)O	-8.5
**185027**	O1C2=C([C]=[C]C(=C2/[C]=[C]/C(=[C])[C])O[C])C(=O)[C][C]1C1=[C][C]=[C][C]=[C]1	-8.5
**126392**	O1C(=[C]C(=O)C2=C1C(=[C][C]=[C]2)[C]C(=O)O[C][C]N([C])[C])C1=[C][C]=[C][C]=[C]1	-8.5
**8395**	O1C(=[C]C(=O)C2=[C][C]=C([C]=C12)O[C]C(=O)O[C][C])C1=[C][C]=[C][C]=[C]1	-8.5
**1889**	O1C2=[C]C(=[C][C]=C2C(=O)[C][C]1C1=[C][C]=C([C]=[C]1)O)O	-8.5
**12983023 9**	C1(=[C]C(=C([C]=C1c1c(c(=O)c2=C([C]=C([C]=c2o1)O)O)O)O)O)S(=O)(=O)C1=[C][C]=C([C]=[C]1)[C]	-8.4
**12982555 6**	o1c2=[C]C(=C(C(=c2c(=O)c(c1C1=[C]C(=C([C]=[C]1)O)/[C]=[C]/C(=[C])[C])O[C])O)O[C])O	-8.4
**12981335 4**	O1C2=[C]C(=[C][C]=C2C(=O)[C][C]1C1=[C]C(=C(C(=[C]1)O)O)O)O[C]	-8.4
**12978128 8**	O1[C]=C(C(=C2[C]=[C][C]=[C][C]12)[N]O)C1=[C][C]=[C][C]=[C]1	-8.4
**10206428 8**	O1C(=[C]C(=O)C2=C1C(=[C][C]=[C]2)/[C]=[C]/[C])C1=[C][C]=[C][C]=[C]1	-8.4
**10201716 5**	O1C(=[C]C(=O)C2=C([C]=C(C(=C12)[C][C]C(=[C])[C])O)O)C1=[C][C]=[C][C]=[C]1	-8.4

**101763632**	C1(=C(C2=C([C]=C1O)O[C]=C(C2=O)C1=[C][C]=C([C]=[C]1)O)O)[C]C([C])([C])O	-8.4
**101680706**	C1(=C2C(=[C][C]=C1C(=O)[C]Br)C(=O)[C]=C(O2)C1=[C][C]=[C][C]=[C]1)C(=O)[C]Br	-8.4
**101621206**	O1C2=[C][C]=C([C])[C]=C2C(=O)[C][C]1C1=[C][C]=C([C]=[C]1)N([C])[C]	-8.4
**101591280**	C1(=[C][C]=C2C(=C1O[C])C(=O)[C]=C(O2)C1=[C][C]=C(C(=[C]1)O[C])O)O[C][C][C][C][C]	-8.4
**101499235**	C1(=[C]C(=C(C(=[C]1)O[C])O[C]([C]O)[C](C1=[C][C]=C([C]=[C]1)O)O)O[C])[C]1OC2=C(C(=[C]C(=[C]2)O)O)C(=O)[C]1	-8.4
**101028404**	O1C(=[C]C(=O)C2=C1C(=C([C]=C2O)O)N[C]([C])[C])C1=[C][C]=[C][C]=[C]1	-8.4
**101028403**	O1C(=[C]C(=O)C2=C1[C]=C(C(=C2O)N[C][C]=[C])O)C1=[C][C]=[C][C]=[C]1	-8.4
**100943385**	ClC1=C(C(=[C]C(=[C]1)C1=[C]C(=O)C2=[C][C]=[C][C]=C2O1)S(=O)(=O)Cl)O[C]	-8.4
**87771765**	[C]1(C(=O)C2=C([C]=[C][C]=[C]2)O[C@]1(C1=[C][C]=[C][C]=[C]1)C(=O)C1=[C]C(=C(C(=[C]1)O)O)O)OS([O])([O])O	-8.4
**73829901**	O1C2=[C]C3=C(C(=C2C(=O)[C]([C]1C1=[C][C]=[C][C]=[C]1)O)O[C])O[C]O3	-8.4
**71621986**	C1(=C([C]=C2C(=[C]1)O[C]=C(C2=O)C1=[C][C]=C([C]=[C]1)O)O[C])O[C]1O[C]([C]([C]([C]1O)O)O[C])[C]O	-8.4
**70350763**	O1C2=[C][C]=[C][C]=C2C(=O)/C(=[C]\O)/[C]1C1=[C][C]=[C][C]=[C]1	-8.4
**66783912**	C1(=[C]C(=O)C2=C(C3=C([C]=C2O1)O[C]O3)O)C1=[C][C]=C([C]=[C]1)O[C][C]N([C][C])[C][C]	-8.4
**57340187**	O1C2=[C]C(=[C][C]=C2C(=O)[C]=C1C1=[C][C]=C([C]=[C]1)/[C]=[C]/C(=O)O)O	-8.4
**53398699**	C1(=[C]C2=C([C]=[C]1)C(=O)C(=[C]O2)C1=[C][C]=C([C]=[C]1)O)O[C]1O[C]([C]([C]([C]1O)O)O)[C]OC(=O)[C]	-8.4
**44265523**	C1(=[C]C(=O)C2=C(C(=C([C]=C2O)O)C2=[C][C]N([C])[C][C]2)O1)C1=C(I)[C]=[C][C]=[C]1	-8.4
**44257645**	O1C2=[C]C(=C3C(=O)[C]=C(OC3=C2[C]([C]1)[C](C([C])([C])OC(=O)[C])OC(=O)[C])C1=[C][C]=[C][C]=[C]1)O[C]	-8.4
**44257318**	O1[C]=C(C(=O)C2=C1C(=C([C]=C2O)O)[C]/[C]=C(\[C])/[C]O)C1=[C][C]=C([C]=C1O)O	-8.4
**25022738**	O1C2=[C]C(=C([C]=C2C(=O)[C][C]1C1=[C][C]=[C][C]=[C]1)O[C])O	-8.4
**23644938**	c1(c(c(=O)c2[C]=[C]C(=[C]c2o1)O[C])C1=[C][C]=[C][C]=[C]1)SC1=N[C]=NN1	-8.4
**21270125**	C1(=[C]OC2=C([C]=C(C(=[C]2)O)O)C1=O)C1=[C][C]=C(Cl)[C]=[C]1	-8.4
**18324915**	c1(c(c(=O)c2[C]=[C][C]=[C]c2o1)C1=[C]C(=[C][C]=[C]1)F)C1=[C][C]=C([C]=[C]1)S(=O)(=O)[C]	-8.4

**16215025**	C1(=C2C(=C(C(=[C]1)O[C])O[C])O[C]([C]C2=O)C1=[C][C]=[C][C]=[C]1)O[C]1O[C]([C]([C]([C]1O)O)O)[C]O	-8.4
**15491286**	o1c(c(c(=O)c2c(c(c(c12)O)O[C])[C][C]=C([C])[C])O)O)C1=[C]C(=C([C]=[C]1)O)O[C]	-8.4
**15485967**	O1C2=C(C(=C(C(=[C]2)O[C])[C][C]=C([C])[C])O)C(=O)[C][C]1C1=[C][C]=C([C]=[C]1)O	-8.4
**15458309**	O1C2=C(C(=[C][C]=C2C(=O)[C][C]1C1=[C][C]=C([C]=[C]1)O[C])O[C])O[C]	-8.4
**15385487**	O1C2=[C]C(=[C][C]=C2C(=O)[C][C]1C1=[C][C]=C(C(=[C]1)O[C])O)O	-8.4
**15301053**	O1[C]=C(C(=O)C2=C1[C]=C([C]=C2O)O)C1=[C]C2=C([C]=[C]1)O[C]O2	-8.4
**14885875**	O1C2=[C]C(=[C]C(=C2[C][C][C]1C1=[C][C]=[C][C]=[C]1)O[C])O	-8.4
**12813783**	O1C2=C([C]=[C][C]=[C]2)C(=O)[C]=C1C1=[C]C(=[C][C]=[C]1)[C]	-8.4
**12813780**	O1C2=C([C]=[C][C]=[C]2)C(=O)[C][C]1C1=[C]C(=[C][C]=[C]1)[C]	-8.4
**12407921**	O1C2=C([C]=C([C]=[C]2)C(=O)O)C(=O)[C]=C1C1=[C][C]=C([C]=[C]1)O[C][C]O	-8.4
**11739635**	C1(=[C]C(=C(C(=[C]1)O)O[C])[C][C]=C([C])[C])C1=[C]OC2=[C]C(=[C][C]=C2C1=O)O	-8.4
**11704980**	o1c2[C]=[C][C]=[C]c2c(=O)c(c1C1=[C]C(=C(C(=[C]1)O[C])O[C])O[C])C(=O)C1=[C][C]=C([C]=[C]1)O[C]	-8.4
**11583077**	C1(=[C][C]=C2C(=O)[C]=C(OC2=[C]1)C1=[C]C(=C(C(=[C]1)O[C])O[C])O[C])O[C][C](O)[C]N[C]([C])[C]	-8.4
**11494111**	C1(=[C]OC2=[C]C(=[C]C(=C2C1=O)O)O)C1=[C][C]=C(C2=C1[C][C]C(O2)([C])[C])O	-8.4
**11149874**	O1C2=[C][C]=[C][C]=C2C(=O)[C][C]1C1=[C][C]=C([C]=[C]1)N(=O)=O	-8.4
**10980660**	O1C2=C(C(=[C]C(=C2C(=O)[C][C]1C1=[C][C]=[C][C]=[C]1)O)O[C])/[C]=[C]/C(=O)[C]	-8.4
**10901472**	o1c2=[C][C]=[C][C]=c2c(=O)c(c1C1=[C][C]=C([C]=[C]1)[C]=[C])O	-8.4
**10659060**	O1C2=C([C]=[C][C]=[C]2)C(=O)[C]=C1C1=[C]C(=[C][C]=[C]1)[C]C#N	-8.4
**10357090**	C1(=[C][C]=[C]C2=C1O[C]=C(C2=O)C1=[C][C]=[C][C]=[C]1)O[C][C]1O[C]1	-8.4
**10244935**	O1C(=[C]C(=O)C2=C(C(=C([C]=C12)O)N[C][C][C])O)C1=[C][C]=[C][C]=[C]1	-8.4
**10108482**	C1(=[C][C]=C2C(=O)C(=[C]OC2=[C]1)C1=[C][C]=[C][C]=[C]1)O[C][C]1O[C]1	-8.4
**9883305**	o1c(c(c(=O)c2=C(C3=C([C]=c12)O[C]O3)O)O)C1=[C][C]=C([C]=[C]1)O	-8.4
**5393156**	O1C(=[C]C(=O)C2=C([C])[C]=C([C]=C12)O)C1=[C][C]=[C][C]=[C]1	-8.4
**5391140**	O1C(=[C]C(=O)C2=[C][C]=C([C]=C12)O)C1=[C]C(=[C][C]=[C]1)O	-8.4
**5378171**	O1C(=[C]C(=O)C2=C([C]=C([C])[C]=C12)O)C1=[C][C]=C([C]=[C]1)O[C]	-8.4
**5320693**	O1C2=[C]C(=[C][C]=C2C(=O)[C]=C1C1=[C][C]=C([C]=[C]1)O[C])O	-8.4
**5317480**	O1[C]=C(C(=O)C2=C1C(=C([C]=C2O)O)[C][C]=C([C])[C])C1=[C][C]=C([C]=[C]1)O	-8.4
**5281670**	o1c2=[C]C(=[C]C(=c2c(=O)c(c1C1=C([C]=C([C]=[C]1)O)O)O)O)O	-8.4
**5281601**	O1C(=[C]C(=O)C2=C1[C]=C([C]=C2O)O[C])C1=[C][C]=C([C]=[C]1)O[C]	-8.4

**5272796**	O1C2=C([C]=[C][C]=[C]2)C(=O)[C]=C1C1=[C][C]=C([C]=[C]1)C(=O)O	-8.4
**2734580**	O1C2=C([C]=C([C]=[C]2)O)C(=O)[C][C]1C1=[C][C]=[C][C]=[C]1	-8.4
**1232441**	O1C2=C([C]=[C][C]=[C]2)[C][C][C]1C1=[C][C]=[C][C]=[C]1	-8.4
**688859**	O1C2=[C][C]=[C][C]=C2C(=O)[C][C]1C1=[C][C]=C([C]=[C]1)O	-8.4
**688857**	O1C2=C([C]=[C]C(=[C]2)O)C(=O)[C][C]1C1=[C][C]=[C][C]=[C]1	-8.4
**688835**	o1c2[C]=[C]C(=[C]c2c(=O)c(c1C1=[C]C(=[C][C]=[C]1)O[C])O)O[C]	-8.4
**676310**	O1C(=[C]C(=O)C2=[C][C]=C(C(=C12)O)O)C1=[C]C(=[C][C]=[C]1)O	-8.4
**676309**	O1C(=[C]C(=O)C2=C1C(=C([C]=[C]2)O)O)C1=[C][C]=[C][C]=C1O	-8.4
**626146**	o1c([C])c(c(=O)c2[C]=[C]C(=[C]c12)O[C][C]=[C])C1=[C][C]=[C][C]=[C]1	-8.4
**466268**	O1C(=[C]C(=O)C2=[C][C]=C([C]=C12)O[C])C1=[C][C]=[C][C]=[C]1	-8.4
**373261**	O1C2=C(C(=[C]C(=[C]2)O)O)C(=O)[C][C]1C1=[C]C(=C([C]=[C]1)O)O	-8.4
**242065**	[C]1([C]C(=O)C2=[C][C]=[C][C]=C2O1)C1=[C][C]=C(Cl)[C]=[C]1	-8.4
**226342**	O1C2=[C][C]=[C][C]=C2C(=O)[C][C]1C1=[C][C]=C(C(=[C]1)O[C])O	-8.4
**165506**	O1C2=[C][C]=[C][C]=C2C(=O)[C][C]1C1=[C][C]=C([C]=[C]1)O	-8.4
**145726**	O1C2=C([C]=[C][C]=[C]2)C(=O)[C]=C1C1=[C]C(=C([C]=[C]1)O)O	-8.4
**97860**	O1C2=C([C]=C([C]=[C]2)O[C])C(=O)[C][C]1C1=[C][C]=[C][C]=[C]1	-8.4
**55750**	C1(=[C][C]=[C]C2=C1OC(=[C]C2=O)C1=[C][C]=[C][C]=[C]1)[C]C(=O)O[C][C]N([C][C])[C][C]	-8.4
**27443**	o1c(c([C])c(=O)c2=[C]C(=[C][C]=c12)[C]N([C][C])[C][C])C1=[C][C]=[C][C]=[C]1	-8.4
**20489**	O1C(=[C]C(=O)C2=C([C]=C(C(=C12)O)O[C])O)C1=[C][C]=[C][C]=[C]1	-8.4
**1890**	O1C2=C([C]=[C]C(=[C]2)O)C(=O)[C][C]1C1=[C][C]=[C][C]=[C]1	-8.4
**13253615 8**	O1C(=[C]C(=O)C2=C1[C]=C([C]=C2OC(=O)[C]=[C])OC(=O)[C]=[C])C1=[C][C]=C([C]=[C]1)OC(=O)[C]=[C]	-8.3
**13247200 6**	o1c2[C]=C([C])[C]=C(c2c(=O)c([C])c1C1=[C][C]=C([C])[C]=[C]1)O	-8.3
**12986429 4**	O1C2=C([C]=[C][C]=C2C(=O)[C][C]1C1=[C][C]=C([C]=[C]1)O)O[C]	-8.3
**12985216 0**	C1(=c2c(=[C][C]=[C]1)oc(c(c2=O)O)C1=[C][C]=[C][C]=[C]1)[C]1O[C]([C]([C]([C]1O)O)O)[C]O	-8.3

**129835695**	O1C2=[C][C]=[C][C]=C2C(=O)[C@@]([C@]1(C1=[C][C]=[C][C]=[C]1)O)(c1c(=O)c2=[C][C]=[C][C]=c2oc1C1=[C][C]=[C][C]=[C]1)O	-8.3
**129826769**	S=C1C2=C([C]=[C][C]=[C]2)O[C]([C]1)C1=[C][C]=C([C])[C]=[C]1	-8.3
**129824456**	O1C(=[C]C(=C2[C]C(=[C][C]=C12)[C]=O)[N]O)C1=[C][C]=[C][C]=[C]1	-8.3
**129730222**	O1C(=[C]C(=O)C2=C1C(=[C]C(=[C]2)[C])C(=O)[C][C])C1=[C][C]=[C][C]=[C]1	-8.3
**129689875**	O1C(=[C]C(=O)C2=C([C]=[C][C]=C12)OC(O)(O)O)C1=[C][C]=[C][C]=[C]1	-8.3
**102360079**	O1C2=C(C3=C(C(=[C]2)O[C])C(=O)[C][C](O3)C2=[C][C]=[C][C]=[C]2)[C]([C](C1([C])[C])OC(=O)[C])OC(=O)[C]	-8.3
**102154220**	O1C2=[C]C3=C(C(=O)[C]=C(O3)C3=[C][C]=C([C]=[C]3)O)C(=C2[C]([C]C1([C])[C])O)O	-8.3
**102091267**	O1C2=[C][C]=[C][C]=C2[C]([C][C]1C1=[C][C]=[C][C]=[C]1)[N]OC(=O)[C]	-8.3
**101843484**	C1(=[C][C]=[C]C2=C1C(=O)[C]=C(O2)C1=[C][C]=C([C]=[C]1)O)O[C]1O[C]([C]([C]([C]1O)O)O[C])[C]O	-8.3
**101685251**	C1(=[C][C]=C([C]=[C]1)C(=O)N[C][C]O)C1=[C]C(=O)C2=[C][C]=[C][C]=C2O1	-8.3
**101685249**	C1(=[C][C]=C([C]=[C]1)C(=O)N[C][C]O)[C]1OC2=C([C]=[C][C]=[C]2)C(=O)[C]1	-8.3
**101591998**	C1(=[C]C(=C2C(=O)[C]=C(OC2=C1O[C])C1=[C][C]=[C][C]=[C]1)O[C])O[C]1O[C]([C]([C]([C]1O)O)O)[C]O	-8.3
**101499236**	C1(=C([C]=C([C]=C1O[C])C1=[C]C(=O)C2=C([C]=C([C]=C2O)O)O1)O[C])O[C]([C]O)[C](C1=[C][C]=C([C]=[C]1)O)O	-8.3
**101028408**	O1C(=[C]C(=O)C2=C([C]=C(C(=C12)N[C][C][C]([C])[C])O[C])O[C])C1=[C][C]=[C][C]=[C]1	-8.3
**101028405**	O1C(=[C]C(=O)C2=C1C(=C([C]=C2O[C])O[C])N[C]([C])[C])C1=[C][C]=[C][C]=[C]1	-8.3

**100914052**	C1(=[C][C]=C([C]=[C]1)c1c(c(=O)c2[C]=[C][C]=[C]c2o1)O)N1[C][C]O[C][C]O[C][C]O[C][C]O[C][C]1	-8.3
**89858365**	o1c(c(c(=O)c2C(=[C][C]=[C]c12)C(=O)[C])O)C1=[C][C]=[C][C]=[C]1	-8.3
**86236611**	C1(=[C][C]=C2C(=O)[C]=C(OC2=[C]1)C1=[C]C(=C([C]=[C]1)O[C])O[C])O[C][C]1O[C]1	-8.3
**85751070**	[N](C1=C2C(=[C][C]=[C][C]2)OC(=[C]1)C1=[C][C]=[C][C]=[C]1)NC(=S)N	-8.3
**71762131**	O1C(=[C]C(=O)C2=C1C(=C([C]=[C]2)O)O[C])C1=[C][C]=[C][C]=[C]1	-8.3
**71621984**	C1(=[C]C(=C2C(=O)C(=[C]OC2=[C]1)C1=[C][C]=C([C]=[C]1)O)O)O[C]1O[C]([C]([C]([C]1O)O)O[C])[C]O	-8.3
**69234627**	O([C@@]1([C][C]C2=[C][C]=[C][C]=C2O1)C1=[C][C]=[C][C]=[C]1)[C]1O[C]([C]([C]([C]1O)O)O)[C]O	-8.3
**67114276**	o1c(c(c(=O)c2=C([C]=[C][C]=c12)C(=O)[C])C(=O)[C])C1=[C][C]=[C][C]=[C]1	-8.3
**66650373**	C1(=[C][C]=[C]C2=C1C(=O)[C]=C(O2)C1=[C][C]=[C][C]=[C]1)[C]Br	-8.3
**66561376**	o1c(c(c(=O)c2c(c3c(c(c12)O[C])O[C]O3)O)O[C])C1=[C]C2=C([C]=[C]1)O[C]O2	-8.3
**57409964**	C1(=[C]C(=C(C(=[C]1)[C][C](C(=[C])[C])O)O)[C]/[C]=C(/[C])\[C]O)c1oc2=[C]C(=C(C(=c2c(=O)c1O[C])O)O[C])O	-8.3
**57340184**	C1(=[C]C(=O)C2=C([C]=C([C]=[C]2)O)O1)C1=[C][C]=C(F)[C]=[C]1	-8.3
**54553686**	O1C2=C([C]=[C][C]=[C]2)C(=O)[C][C]1C1=[C]C(=[C][C]=[C]1)C#N	-8.3
**53247999**	C1(=[C][C]=C([C]=[C]1)[C]1[C]C(=O)C2=[C][C]=[C][C]=C2O1)O[C]1O[C]([C]([C]([C]1O)O)O)[C]	-8.3
**53247946**	C1(=[C]C2=C([C]=[C]1)O[C]([C]C2=O)C1=[C][C]=C([C]=[C]1)O)O[C]1O[C]([C]([C]([C]1O)O)O[C])[C]O	-8.3
**45933941**	O1C2=[C][C]=[C][C]=C2C(=O)[C]=C1C1=[C]C(=[C]C(=[C]1)O)O	-8.3
**44380894**	O1C(=[C]C(=O)C2=C([C]=C(C(=C12)N[C][C][C])O)O)C1=[C][C]=[C][C]=[C]1	-8.3
**44259965**	o1c(c(c(=O)c2=C([C]=C(C(=c12)O)O[C])O)O)C1=[C][C]=C([C]=[C]1)O	-8.3
**44259864**	o1c(c(c(=O)c2c(c(c3c([C]=[C]O3)c12)O[C])O[C])O[C])C1=[C]C2=C([C]=[C]1)O[C]O2	-8.3
**44258614**	O1c2c3C(=O)[C]=C(Oc3c(c(c2[C]=[C]C1([C])[C])O[C])O[C])C1=[C][C]=C(C(=[C]1)O[C])O[C]	-8.3
**44258526**	O1C(=[C]C(=O)C2=C1[C]=C(C1=C2O[C]O1)O[C])C1=[C][C]=C2C(=[C]1)O[C]O2	-8.3
**42608071**	O1C2=C(C(=[C]C(=C2C(=O)[C][C]1C1=[C][C]=C([C]=[C]1)O)O)O[C])[C][C]=C([C])[C]	-8.3
**42608069**	O1C2=C(C(=[C]C3=C2[C]=[C]C(O3)([C])[C])O[C])C(=O)[C][C]1C1=[C]C(=C([C]=[C]1)O[C])O[C]	-8.3
**23644937**	c1(c(c(=O)c2[C]=[C]C(=[C]c2o1)O[C])C1=[C][C]=[C][C]=[C]1)SC1=N[C]=[C]N1[C]	-8.3
**20452436**	O1C2=C([C]=[C][C]=[C]2)[C][C][C]1C1=[C][C]=C([C]=[C]1)O	-8.3
**20394007**	C1(=[C][C]=C2C(=O)C(=[C]OC2=[C]1)C1=[C][C]=[C][C]=[C]1)O[C]([C])[C][C]	-8.3
**20301086**	O1C2=[C][C]=[C][C]=C2C(=O)C(=[C])[C]1C1=[C]C(=[C][C]=[C]1)N(=O)=O	-8.3
**15896301**	O1C(=[C]C(=O)c2c(c(c(c(c12)O[C])O[C])O[C])O)C1=[C][C]=[C]C(=C1O)O[C]	-8.3

**14034288**	O1C2=C(C(=[C]C(=C2O)O[C])O)C(=O)[C][C]1C1=[C][C]=[C][C]=C1O	-8.3
**12674946**	o1c2=[C][C]=[C][C]=c2c(=O)c(c1C1=[C][C]=[C][C]=[C]1)C(=O)O	-8.3
**12114693**	O1[C]([C]C(=O)C2=C1C(=C([C]=C2O)O)C([C])([C])[C]=[C])C1=[C][C]=C([C]=[C]1)O	-8.3
**12098358**	O1C2=C(C(=[C]C(=C2O[C])O[C])O)C(=O)[C][C]1C1=[C][C]=[C][C]=C1O	-8.3
**11661570**	C1(=[C]C2=C([C]=[C]1)C(=O)[C]=C(O2)C1=[C]C(=[C]C(=[C]1)O[C])O[C])O[C][C](O)[C]N[C]([C])[C]	-8.3
**11382711**	C1(=C(C2=C([C]=C1O)O[C]=C(C2=O)C1=[C][C]=C([C]=[C]1)O)O)[C][C]C([C])([C])O	-8.3
**11246736**	c1(c(c(=O)c2[C]=[C]C(=[C]c2o1)O[C])C1=[C][C]=C([C]=[C]1)O[C])S[C]C1=[C][C]=N[C]=[C]1	-8.3
**11176755**	c1(c(c(=O)c2[C]=[C]C(=[C]c2o1)O[C])C1=[C][C]=C([C]=[C]1)[C])S[C]C1=[C][C]=N[C]=[C]1	-8.3
**10904774**	C1(=C([C]=C(C2=C1O[C]=C(C2=O)C1=C([C]=C([C]=[C]1)O)O)O)O)[C][C]C([C])([C])O	-8.3
**10902653**	C1(=[C]C(=O)C2=[C][C]=[C][C]=C2O1)C1=[C][C]=C([C]=[C]1)S(=O)(=O)[C]	-8.3
**10777811**	O1C2=[C][C]=[C][C]=C2C(=O)[C]=C1C1=[C][C]=C([C]=[C]1)[C]C#N	-8.3
**10742453**	O1[C]([C]C(=O)C2=C1C(=C([C]=C2O)O)[C][C](C(=[C])[C])[C][C]C([C])([C])O)C1=C([C]=C([C]=[C]1)O)O[C]	-8.3
**10736576**	O1C2=[C]C3=C(C(=O)C(=[C]O3)C3=[C][C]=C([C]=[C]3)O)C(=C2[C]=[C]C1([C])[C])O[C]	-8.3
**10498462**	O1C2=C(C(=C(C(=[C]2)O)C([C])([C])[C]=[C])O)C(=O)[C][C]1C1=[C]C(=C([C]=[C]1)O)O	-8.3
**10335613**	O1C(=[C]C(=O)C2=C(C(=C([C]=C12)O)N[C]([C])[C])O)C1=[C][C]=[C][C]=[C]1	-8.3
**10022760**	C1(=[C]c2c(C(=[C]1)O)c(=O)c(c(o2)C1=[C]C(=C([C]=[C]1)O)O)O)OS([O])([O])O	-8.3
**9813808**	O1C2=[C][C]=[C][C]=C2[C][C]([C]1C1=[C]C(=C([C]=[C]1)O)O)O	-8.3
**9562299**	[N]([C]1C2=[C][C]=[C][C]=C2O[C]([C]1)C1=[C][C]=[C][C]=[C]1)NC(=S)N	-8.3
**5321859**	O1C(=[C]C(=O)c2c(c(c(c(c12)O[C])O)O[C])O)C1=[C]C(=C([C]=[C]1)O)O	-8.3
**5317435**	O1C2=[C]C(=[C][C]=C2C(=O)[C]([C]1C1=[C][C]=C(C(=[C]1)O)O)O)O	-8.3
**5316733**	O1C2=[C]C(=C(C(=C2C(=O)[C][C]1C1=[C][C]=[C][C]=[C]1)O)O[C])O	-8.3
**5282073**	O1C(=[C]C(=O)C2=[C][C]=C([C]=C12)O)C1=[C][C]=C([C]=[C]1)O	-8.3
**5281894**	O1C(=[C]C(=O)C2=[C][C]=C([C]=C12)O)C1=[C][C]=[C][C]=[C]1	-8.3
**5281674**	O1C(=[C]C(=O)C2=C([C]=C(C(=C12)O)O)O)C1=[C][C]=[C][C]=[C]1	-8.3
**5280445**	O1C(=[C]C(=O)C2=C1[C]=C([C]=C2O)O)C1=[C][C]=C(C(=[C]1)O)O	-8.3
**3826106**	O1C2=C([C]=[C][C]=[C]2)[C]([C][C]1)C1=[C][C]=[C][C]=[C]1	-8.3
**740761**	C1(=[C]OC2=C([C]=[C]C(=[C]2)O[C]([C])[C])C1=O)C1=C(F)[C]=[C][C]=[C]1	-8.3
**688853**	O1C(=[C]C(=O)C2=[C][C]=C(C(=C12)O)O)C1=[C][C]=C([C]=[C]1)O	-8.3
**688672**	O1C(=[C]C(=O)C2=[C][C]=C([C]=C12)O[C])C1=[C]C(=[C][C]=[C]1)O[C]	-8.3

**676034**	O1C2=C([C]=C([C]=[C]2)O)C(=O)[C]=C1C1=C([C]=[C][C]=[C]1)O[C]	-8.3
**676033**	O1C2=C([C]=[C][C]=[C]2)C(=O)[C]=C1C1=C(C(=[C][C]=[C]1)O[C])O[C]	-8.3
**619834**	O1C2=C([C]=[C][C]=[C]2)C(=O)[C]=C1C1=[C][C]=[C]C(=[C]1)O[C]	-8.3
**441251**	O1[C]=C(C(=O)C2=C(C(=C([C]=C12)O[C])[C][C]=C([C])[C])O)C1=C([C]=C([C]=[C]1)O)O	-8.3
**439652**	O1C2=C([C]=[C][C]=[C]2)C(=O)[C][C]1C1=[C][C]=[C][C]=[C]1	-8.3
**369609**	O1C2=C([C]=C([C]=C2[C]([C][C]1C1=[C][C]=C(C(=[C]1)O[C])O[C])O)[C][C]=[C])O[C]	-8.3
**238782**	O1C2=C(C(=[C]C(=[C]2)O)O)C(=O)[C][C]1C1=[C][C]=[C][C]=[C]1	-8.3
**177032**	O1C2=C(C(=C(C(=[C]2)O)O[C])O)C(=O)[C][C]1C1=[C][C]=[C][C]=[C]1	-8.3
**102928**	O1C2=C([C]=[C][C]=[C]2)C(=O)[C][C]1C1=[C][C]=C([C]=[C]1)O[C]	-8.3
**76573**	O1C(=[C]C(=O)c2c(c([C])c(c([C])c12)O[C])O)C1=[C][C]=C([C]=[C]1)O[C]	-8.3
**41449**	C1(=[C][C]=C([C]=[C]1)C1=[C]C(=O)C2=[C][C]=[C][C]=C2O1)O[C][C](O)[C]N[C]([C])[C]	-8.3
**18721**	O1C(=[C]C(=O)C2=C1[C]=C([C]=C2OC(=O)[C])OC(=O)[C])C1=[C][C]=C([C]=[C]1)OC(=O)[C]	-8.3
**11095**	O1C2=C(C(=[C]C(=[C]2)O)O)C(=O)[C][C]1C1=[C]C(=C([C]=[C]1)O)O	-8.3
**10251**	O1C2=C([C]=[C][C]=[C]2)C(=O)[C][C]1C1=[C][C]=[C][C]=[C]1	-8.3
**3747**	C1(=[C][C]=C2C(=O)C(=[C]OC2=[C]1)C1=[C][C]=[C][C]=[C]1)O[C]([C])[C]	-8.3
**138402973**	O1[C]=C(C(=O)C2=C1[C]=C(C(=[C]2)O)[C][C][C]O)C1=[C][C]=C([C]=[C]1)O	-8.2
**134820726**	O1C(=[C]C(=O)C2=[C]C(=[C]C(=C12)O[C])O[C])C1=[C][C]=C([C]=[C]1)O[C]	-8.2
**129882404**	O1[C]=C(C(=O)C2=C1[C]=[C][C]=[C]2)C1=[C][C]=C([C]=[C]1)[C]N	-8.2
**129866052**	o1c2[C]=[C][C]=[C]c2c(=O)c(c1C1=[C][C]=[C][C]=[C]1)[C]1[C]N[C][C]N1[C]C1=[C][C]=[C][C]=[C]1	-8.2
**129824479**	O1[C]2[C]=[C][C]=[C]C2=C([C]=C1C1=[C][C]=C([C]=[C]1)[C]=O)[N]O	-8.2
**129820755**	C1(=[C][C]=c2c(=[C]1)c(=O)c(c(o2)C1=[C][C]=[C][C]=[C]1)O)[C](O)[C][C][C][C]	-8.2
**129762930**	O1C2=C(C(=[C]C(=[C]2)O[C])O)C(=O)[C][C@]1(C1=[C][C]=C([C]=[C]1)O)O[C]	-8.2

**129662374**	o1c2=[C][C]=[C][C]=c2c(=O)c(c1[C])C1=[C][C]=C([C]=[C]1)N(=O)=O	-8.2
**121487929**	O1C2=[C][C]=[C][C]=C2C(=O)[C][C]1C1=[C][C]=[C][C]=[C]1	-8.2
**102320040**	O1[C]=C(C(=O)c2c(c(c(c(c12)O)O)O[C])O)C1=[C][C]=C([C]=[C]1)O	-8.2
**101137054**	C1(=C([C]=C(C2=C1O[C]=C(C2=O)C1=[C][C]=C([C]=C1O)O)O)O)[C]C([C])([C])O	-8.2
**101028407**	O1C(=[C]C(=O)C2=C([C]=C(C(=C12)N[C][C]([C])[C])O[C])O[C])C1=[C][C]=[C][C]=[C]1	-8.2
**71777365**	O1C2=[C][C]=[C][C]=C2C(=O)[C][C]1C1=[C][C]=[C][C]=[C]1	-8.2
**71621987**	C1(=[C][C]=C2C(=O)C(=[C]OC2=[C]1)C1=[C][C]=C([C]=[C]1)O)O[C]1O[C]([C]([C]([C]1O)O)O[C])[C]O	-8.2
**69029207**	C1(=[C][C]=C2C(=O)[C]=C(OC2=[C]1)C1=[C]C(=C([C]=[C]1)O[C])O[C])O[C][C][C]Br	-8.2
**57340186**	O1C2=[C]C(=[C][C]=C2C(=O)[C]=C1C1=[C][C]=C([C]=[C]1)/[C]=[C]/C(=O)O[C][C])O	-8.2
**49788612**	o1c2=[C]C(=[C]C(=c2c(=O)c(c1C1=[C]C2=C([C]=[C]1)O[C]O2)O)O)O[C]	-8.2
**44380950**	O1C(=[C]C(=O)C2=C1C(=C([C]=C2O)O)N[C][C]=[C])C1=[C][C]=[C][C]=[C]1	-8.2
**29920547**	O1C(=[C]C(=O)C2=C1[C]=C([C]=C2[C])OC(=O)O[C][C])C1=[C][C]=[C][C]=[C]1	-8.2
**25201487**	O1[C](C(=O)C(=O)C2=[C][C]=[C][C]=C12)C1=[C][C]=[C][C]=[C]1	-8.2
**24866263**	C1(=[C][C]=C([C]=[C]1)C1=[C]C(=O)C2=C([C]=C([C]=C2O1)O)O)O[C]1O[C]([C]([C]([C]1O)O)O)[C]	-8.2
**24866262**	C1(=C([C]=C([C]=[C]1)C1=[C]C(=O)C2=C([C]=C([C]=C2O1)O)O)O[C])O[C]1O[C]([C]([C]([C]1O)O)O)[C]	-8.2
**23644936**	c1(c(c(=O)c2[C]=[C]C(=[C]c2o1)O[C])C1=[C][C]=[C][C]=[C]1)SC1=N[C]=[C]N1	-8.2
**21576250**	O1[C]=C(C(=O)C2=C([C]=C(C(=C12)C([C])([C])[C]=[C])O)O)C1=[C][C]=C([C]=C1O)O	-8.2
**20522556**	c1(c(c(=O)c2C(=[C]C(=[C]c2o1)O[C])O)O[C])C1=[C][C]=C(C(=[C]1)O[C])OC(=O)C([C])([C])[C]	-8.2
**19835350**	C1(=C2C(=[C][C]=[C]1)C(=O)[C]=C(O2)C1=[C][C]=[C][C]=[C]1)O[C][C]1O[C]1	-8.2
**18324995**	c1(c(c(=O)c2[C]=[C][C]=[C]c2o1)C1=C([C]=[C][C]=[C]1)F)C1=[C][C]=C([C]=[C]1)S(=O)(=O)N	-8.2
**15817847**	o1c2=[C]C(=C([C]=c2c(=O)c(c1C1=[C][C]=C(C(=[C]1)O[C])O)O)O)O	-8.2
**15160703**	O1C2=C([C]=C(C3=C2[C]=[C]C(O3)([C])[C])O[C])C(=O)[C][C]1C1=[C]C(=C([C]=[C]1)O[C])O[C]	-8.2
**13942547**	o1c(c(c(=O)c2=C([C]=C(C(=c12)O[C])O[C][C]=C([C])[C])O[C])O[C])C1=[C]C2=C([C]=[C]1)O[C]O2	-8.2
**13908971**	O1C2=C(C3=C(C(=[C]2)O)C(=O)C(=[C]O3)C2=C([C]=C([C]=[C]2)O)O)[C][C]1C(=[C])[C]	-8.2

**13250353**	O1C2=C(C(=[C][C]=[C]2)O)[C][C][C]1C1=[C][C]=[C][C]=[C]1	-8.2
**12735893**	o1c(c(c(=O)c2[C]=[C]C3=C([C]=[C]O3)c12)O[C])C1=[C][C]=C([C]=[C]1)O[C]	-8.2
**12407866**	O1C(=[C]C(=O)C2=C1[C]=[C]C(=[C]2)C(=O)O[C])C1=C([C]=[C][C]=[C]1)O	-8.2
**12261178**	O1C2=C([C]=[C][C]=[C]2)C(=O)[C]=C1C1=[C][C]=C([C]=[C]1)[C]=O	-8.2
**12136839**	O1C2=C([C]=[C][C]=[C]2)C(=O)[C][C]1C1=[C][C]=[C][C]=[C]1	-8.2
**11847318**	c1(c(c(=O)c2[C]=[C][C]=[C]c2o1)[C]N1[C]=N[C]=[C]1)C1=[C][C]=C(Br)[C]=[C]1	-8.2
**11847316**	o1c2=[C][C]=[C][C]=c2c(=O)c(c1C1=[C][C]=[C][C]=[C]1)[C]N1[C]=N[C]=[C]1	-8.2
**11417114**	c1(c(c(=O)c2[C]=[C]C(=[C]c2o1)O)C1=[C][C]=[C][C]=[C]1)S[C]C1=[C]N=[C][C]=[C]1	-8.2
**11142360**	O1[C]=C(C(=O)C2=C1[C]=C1C(=[C]2)O[C]O1)C1=[C]C(=C2C(=[C]1)O[C]O2)O[C]	-8.2
**11032058**	[C]1([C]C(=O)C2=C([C]=[C][C]=[C]2)O1)C1=[C][C]=C(F)[C]=[C]1	-8.2
**10685123**	C1(=[C][C]=C2C(=[C]1)C(=O)[C]=C(O2)C1=[C][C]=[C][C]=[C]1)[C]N=C=S	-8.2
**10613051**	C1(=[C]C(=O)C2=C([C]=[C][C]=[C]2)O1)C1=[C][C]=C([C]=[C]1)[S@](=O)[C]	-8.2
**6450959**	O1[C]=C(C(=O)C2=C([C]=C(C(=C12)[C]/[C]=C(\[C])/[C]O)O)O)C1=[C][C]=C([C]=[C]1)O	-8.2
**5487268**	C1(=C([C]=C(C(=[C]1)O)C1=[C]OC2=[C]C(=[C]C(=C2C1=O)O)O)C([C])([C])[C]=[C])O	-8.2
**5466137**	o1c2=[C]C(=[C]C(=c2c(=O)c(c1C1=[C]C2=C([C]=[C]1)O[C]O2)O[C])O)O[C]	-8.2
**5417907**	o1c2[C]=C([C]=[C]c2c(=O)c(c1[C])C1=[C][C]=C([C]=[C]1)N(=O)=O)O	-8.2
**5356690**	O1C2=[C][C]=[C][C]=C2[C]([C][C]1C1=[C][C]=[C][C]=[C]1)[N]O	-8.2
**5317481**	O1[C]=C(C(=O)C2=C([C]=C(C(=C12)[C]/[C]=C(\[C])/[C]O)O)O)C1=[C][C]=C(C(=[C]1)O)O[C]	-8.2
**5317284**	O1C(=[C]C(=O)C2=C1[C]=C(C(=C2O)O[C])O)C1=[C][C]=C(C(=[C]1)O)O	-8.2
**5310669**	C1(=[C]OC2=C([C]=[C]C(=[C]2)O)C1=O)C1=[C][C]=C(F)[C]=[C]1	-8.2
**5281954**	O1C(=[C]C(=O)C2=C1[C]=C([C]=C2O)O[C])C1=[C][C]=[C][C]=[C]1	-8.2
**5281805**	C1(=[C]OC2=[C]C(=[C][C]=C2C1=O)O)C1=[C]C2=C(O[C]O2)[C]=[C]1	-8.2
**5281703**	O1C(=[C]C(=O)C2=C([C]=C(C(=C12)O[C])O)O)C1=[C][C]=[C][C]=[C]1	-8.2
**5281692**	o1c2[C]=C([C]=[C]c2c(=O)c(c1C1=[C]C(=C(C(=[C]1)O)O)O)O)O	-8.2
**5281616**	o1c(c(c(=O)c2=C([C]=C([C]=c12)O)O)O)C1=[C][C]=[C][C]=[C]1	-8.2
**5281614**	o1c2[C]=C([C]=[C]c2c(=O)c(c1C1=[C]C(=C([C]=[C]1)O)O)O)O	-8.2
**5281612**	O1C2=[C]C(=[C]C(=C2C(=O)[C]=C1C1=[C][C]=C(C(=[C]1)O)O[C])O)O	-8.2
**5280863**	o1c(c(c(=O)c2C(=[C]C(=[C]c12)O)O)O)C1=[C][C]=C([C]=[C]1)O	-8.2
**5280442**	O1C(=[C]C(=O)C2=C1[C]=C([C]=C2O)O)C1=[C][C]=C([C]=[C]1)O[C]	-8.2

**5272801**	O1C2=C([C]=[C][C]=[C]2)C(=O)[C]=C1C1=[C][C]=C([C]=[C]1)C1=N[C][C]O1	-8.2
**3890073**	[N]([C]1C2=[C][C]=[C][C]=C2O[C]([C]1)C1=[C][C]=[C][C]=[C]1)NC(=S)N	-8.2
**3070240**	c1(c([C])c(=O)c2[C]=[C]C(=C(c2o1)[C]N([C])[C])O[C])C1=[C][C]=C(F)[C]=[C]1	-8.2
**689010**	O1C2=C([C]=[C][C]=[C]2)C(=O)[C][C]1C1=[C][C]=[C][C]=[C]1	-8.2
**676299**	O1C(=[C]C(=O)C2=C1C(=C([C]=[C]2)O[C])O)C1=[C][C]=[C][C]=[C]1	-8.2
**462697**	O1C2=[C]C(=[C]C(=C2C(=O)[C]([C]1C1=[C]C(=C([C]=[C]1)O[C])O[C]C1=[C][C]=[C][C]=[C]1)O[C])O)O	-8.2
**439712**	O1C2=[C][C]=[C][C]=C2[C]([C][C]1C1=[C][C]=[C][C]=[C]1)O	-8.2
**439246**	O1C2=[C]C(=[C]C(=C2C(=O)[C][C]1C1=[C][C]=C([C]=[C]1)O)O)O	-8.2
**378567**	O1C2=[C]C(=[C]C(=C2C(=O)[C][C]1C1=[C][C]=[C][C]=[C]1)O[C])O[C]	-8.2
**373260**	O1C(=[C]C(=O)C2=C1[C]=C(C(=C2O)O)O[C])C1=[C][C]=C(C(=[C]1)O)O[C]	-8.2
**344546**	o1c(c(c(=O)c2=[C][C]=C([C]=c12)O[C])O)C1=[C][C]=[C][C]=[C]1	-8.2
**229016**	O1C2=C([C]=[C][C]=[C]2)C(=O)[C]=C1C1=[C][C]=C([C]=[C]1)O	-8.2
**188308**	O1C2=[C]C(=C(C(=C2C(=O)[C][C]1C1=[C][C]=C([C]=[C]1)O)O)O)O	-8.2
**176925**	O1C2=C([C]=[C][C]=[C]2)C(=O)[C][C]1C1=[C][C]=[C][C]=C1O	-8.2
**160921**	O1C(=[C]C(=O)c2c(c(c(c(c12)O[C])O)O[C])O)C1=[C][C]=C([C]=[C]1)O[C]	-8.2
**137327**	O1C(=[C]C(=O)C2=[C]C(=[C]C(=C12)[C]C(=O)O)[C])C1=[C][C]=[C][C]=[C]1	-8.2
**136418**	O1C(=[C]C(=O)C2=C(C(=C([C]=C12)OC(=O)[C])OC(=O)[C])OC(=O)[C])C1=[C][C]=C([C]=[C]1)OC(=O)[C]	-8.2
**100633**	o1c(c(c(=O)c2[C]=[C]C3=C([C]=[C]O3)c12)O[C])C1=[C][C]=[C][C]=[C]1	-8.2
**77793**	O1C2=C([C]=[C][C]=[C]2)C(=O)[C]=C1C1=[C][C]=C([C]=[C]1)O[C]	-8.2
**72281**	O1C2=[C]C(=[C]C(=C2C(=O)[C][C]1C1=[C]C(=C([C]=[C]1)O[C])O)O)O	-8.2
**72279**	O1C(=[C]C(=O)C2=C1[C]=[C]C(=[C]2)O)C1=[C][C]=[C][C]=[C]1	-8.2
**51459**	o1c2=[C][C]=[C][C]=c2c(=O)c([C])c1C1=[C][C]=[C][C]=[C]1	-8.2
**11055**	O1C(=[C]C(=O)C2=C([C]=[C]C(=C12)O)O)C1=[C][C]=[C][C]=[C]1	-8.2
**3593**	O1C2=[C]C(=[C]C(=C2C(=O)[C][C]1C1=[C]C(=C([C]=[C]1)O[C])O)O)O	-8.2
**1880**	O1C(=[C]C(=O)C2=[C][C]=C(C(=C12)O)O)C1=[C][C]=[C][C]=[C]1	-8.2
**932**	O1C2=[C]C(=[C]C(=C2C(=O)[C][C]1C1=[C][C]=C([C]=[C]1)O)O)O	-8.2
**14668157<br>4**	o1c(c(c(=O)c2C(=C3C(=[C]c12)O[C]O3)O)O[C](O)O)C1=[C][C]=[C][C]=[C]1	-8.1

**129858370**	O1C2=C([C]=[C]C(=[C]2)O[C]C(=O)O)C(=O)[C]=C1C1=[C]C(=C(C(=[C]1)O[C])O[C])O[C]	-8.1
**129852882**	[S]([C]1C(=O)c2c(c(c(c(c2O[C]1C1=[C]C(=C([C]=[C]1)O[C])O[C])O[C])O[C])O[C])O[C])([O])[O]	-8.1
**129834540**	C1(=C([C]=C2C(=C1O)C(=O)[C]=C(O2)C1=[C][C]=C([C]=[C]1)[C])O)S([O])([O])O	-8.1
**129829598**	o1c(c(c(=O)c2c(c(c(c(c12)O)O)O)O[C])O)C1=[C][C]=[C][C]=[C]1	-8.1
**129824490**	O1C2=[C][C]=[C][C]C2=C([C]=C1C1=[C][C]=C([C]=[C]1)[C]=O)[N]O[C][C]	-8.1
**129819557**	O1C2=C([C]=[C][C]=[C]2)C(=O)[C]([C]1C1=[C][C]=[C][C]=[C]1)O	-8.1
**129732270**	o1c2=[C][C]=[C][C]=c2c(=O)c(c1C1=[C][C]=[C][C]=[C]1)OO	-8.1
**129711278**	C1(=[C][C]=[C]C2=C1C(=O)[C]=C(O2)C1=[C][C]=[C][C]=[C]1)C(O)(O)[C]([C]([C])[C])O	-8.1
**129684668**	o1c(c(c(=O)c2c(c(c(c12)O)O)/[C]=[C]/C(=[C])[C])O)O)C1=[C][C]=[C][C]=[C]1	-8.1
**102577926**	O1C(=[C]C(=O)C2=C1[C]=C([C]=C2O)O)C1=[C][C]=C([C]=[C]1)O[C]O[C]	-8.1
**102005374**	C1(=[C]C(=O)C2=C(C(=C([C]=C2O[C])O[C])[C]2[C][C][C][C][C]2O)O1)C1=C(Cl)[C]=[C][C]=[C]1	-8.1
**101930613**	O1[C]2[C]=[C][C]=[C]C2=[C][C]=C1C1=[C][C]=C([C]=[C]1)NC([C])([C])[C]	-8.1
**73829902**	O1C2=C(C(=[C][C]=C2C(=O)[C]([C]1C1=[C][C]=C(C(=[C]1)O)O)O)O)O	-8.1
**70018578**	O1C2=[C]C(=[C][C]=C2C(=O)[C][C]1C1=[C][C]=C([C]=[C]1)O[C])O	-8.1
**57368437**	o1c(c(c(=O)c2[C]=C([C]=C(c12)O)O)O)C1=[C][C]=C([C]=[C]1)O	-8.1
**57363368**	o1c(c(c(=O)c2[C]=C([C]=C(c12)O)O)O)C1=[C][C]=C([C]=[C]1)O[C]	-8.1
**54070148**	C1(=[C][C]=C([C]=[C]1)O[C][C]O)[C]1OC2=[C][C]=[C][C]=C2[C][C]1	-8.1
**53233892**	C1(=[C]C2=C([C]=[C]1)OC(=[C]C2=O)C1=[C][C]=[C][C]=[C]1)OP(=O)(O[C][C])O[C][C]	-8.1

**44260056**	O1C(=[C]C(=O)c2c(c(c(c(c12)O[C])O[C])OC(=O)[C]([C])[C])O)C1=[C][C]=C([C]=[C]1)O	-8.1
**44260022**	o1c(c(c(=O)c2C(=[C]C(=C(c12)O[C])O[C][C]=C([C])[C])O)O[C])C1=[C][C]=C(C(=[C]1)O[C])O	-8.1
**44258732**	o1c(c(c(=O)c2c(c([C])c(c([C])c12)O[C])O)O)C1=[C][C]=[C][C]=[C]1	-8.1
**44258533**	O1C(=[C]C(=O)C2=C(C(=C([C]=C12)O[C])O[C])O[C])C1=[C]C(=C(C(=[C]1)O[C])O[C][C]=C([C])[C])O[C]	-8.1
**44257359**	O1[C]=C(C(=O)C2=C1[C]=C1C(=C2O[C])O[C]O1)C1=[C]C(=C([C]=[C]1)O[C])O	-8.1
**42608063**	O1C2=C(C(=[C]C3=C2[C]=[C]C(O3)([C])[C])O[C])C(=O)[C][C]1C1=[C][C]=C([C]=[C]1)O[C]	-8.1
**25022469**	O1C(=[C]C(=O)C2=C(C(=[C]C(=C12)O[C])O[C])O[C])C1=C([C]=[C]C(=[C]1)O[C])O[C]	-8.1
**21636237**	O1C2=C(C(=[C]C(=C2O[C])O[C])O)C(=O)[C][C]1C1=[C][C]=[C][C]=C1O[C]	-8.1
**21626042**	O1[C]=C(C(=O)C2=C1[C]=C1C(=C2O[C])O[C]O1)C1=C([C]=C2C(=[C]1)O[C]O2)O[C]	-8.1
**21325037**	C1(=[C]C2=C([C]=[C]1)OC(=[C]C2=O)C1=C([C]=[C][C]=[C]1)O[C]([C])[C])C(=O)Cl	-8.1
**21270134**	C1(=[C]OC2=C([C]=C(C(=[C]2)O)O)C1=O)C1=[C]C(=[C][C]=[C]1)Cl	-8.1
**20452422**	O1C2=C([C]=[C][C]=[C]2)[C][C][C]1C1=[C][C]=C([C]=[C]1)N([C])[C]	-8.1
**18325002**	c1(c(c(=O)c2[C]=[C][C]=[C]c2o1)C1=C([C]=[C][C]=[C]1)F)C1=[C][C]=C([C]=[C]1)S(=O)(=O)[C]	-8.1
**14804713**	o1c(c(c(=O)c2=C([C])[C]=C([C]=c12)O[C])O[C])C1=[C][C]=C([C]=[C]1)O	-8.1
**12310641**	O1C2=[C]C(=[C][C]=C2C(=O)[C]([C]1C1=[C][C]=C(C(=[C]1)O)O)O)O	-8.1
**11847319**	o1c2=[C][C]=[C][C]=c2c(=O)c(c1C1=[C][C]=C([C]=[C]1)C#N)[C]N1[C]=N[C]=[C]1	-8.1
**11810763**	O1[C]=C(C(=O)C2=C(C3=C([C]=C12)O[C]O3)O[C])C1=[C]C(=C2C(=[C]1)O[C]O2)O[C]	-8.1
**11485667**	c1(c(c(=O)c2[C]=[C]C(=[C]c2o1)O[C])C1=[C][C]=[C][C]=[C]1)S[C]C1=[C][C]=[C][C]=N1	-8.1
**11316719**	O1C(=[C]C(=O)C2=C1[C]=C(C(=C2OC(=O)[C])OC(=O)[C])O[C]C1=[C][C]=[C][C]=[C]1)C1=[C][C]=[C][C]=[C]1	-8.1
**11164739**	c1(c(c(=O)c2=[C][C]=C([C]=c2o1)O[C])C1=[C][C]=[C][C]=[C]1)S[C]C1=[C][C]=[C]N=[C]1	-8.1
**11153057**	c1(c(c(=O)c2[C]=[C]C(=[C]c2o1)O[C])C1=[C][C]=[C][C]=[C]1)S[C]C1=[C][C]=N[C]=[C]1	-8.1
**10814211**	o1c(c(c(=O)c2c(c(c(c(c12)O[C])O[C][C]=C([C])[C])O[C])O[C])O[C])C1=[C][C]=C2C(=[C]1)O[C]O2	-8.1
**10665235**	o1c(c(c(=O)c2=C([C]=C(C(=c12)O[C])O[C])O)O)C1=[C]C(=C([C]=[C]1)O)O	-8.1
**10612616**	O1C(=[C]C(=O)C2=C1[C]=[C][C]=C2O[C][C]=[C])C1=[C][C]=[C][C]=[C]1	-8.1
**10069029**	c1(c(c(=O)c2[C]=[C]C(=[C]c2o1)O)C1=[C][C]=C([C]=[C]1)O[C])S[C]C1=[C][C]=N[C]=[C]1	-8.1
**9821032**	c1(c(c(=O)c2=[C][C]=[C][C]=c2o1)C1=[C][C]=[C][C]=[C]1)C1=[C][C]=C([C]=[C]1)S(=O)(=O)[C]	-8.1
**6077518**	O1C2=C([C]=[C][C]=[C]2)[C]([C][C]1C1=[C][C]=[C][C]=[C]1)[N]O[C][C]N([C][C])[C][C]	-8.1
**5491929**	O1[C]=C(C(=O)C2=C1[C]=C1C(=C2O)O[C]O1)C1=C([C]=[C][C]=[C]1)O[C]	-8.1
**5490139**	O1C2=[C]C3=C(C(=O)C(=[C]O3)C3=[C][C]=C([C]=[C]3)O)C(=C2[C]=[C]C1([C])[C])O	-8.1

**5469524**	O1C(=[C]C(=O)C2=C1[C]=C(C(=C2O)O[C])O)C1=[C][C]=C(C(=[C]1)O)O[C]	-8.1
**5465888**	O1C2=[C][C]=[C][C]=C2/C(=N/N(C(=O)[C])C(=O)[C])/[C][C]1C1=[C][C]=[C][C]=[C]1	-8.1
**5393152**	o1c(c(c(=O)c2=[C][C]=C([C]=c12)O)O)C1=[C][C]=[C][C]=[C]1	-8.1
**5385026**	o1c(c(c(=O)c2C(=C(C(=[C]c12)O)O[C])O[C])O)C1=[C][C]=[C][C]=[C]1	-8.1
**5378518**	O1C2=C([C]=[C]C(=[C]2)O)C(=O)[C]=C1C1=[C]C(=C([C]=[C]1)O[C])O[C]	-8.1
**5378202**	C1(=C([C]=C(C2=C1O[C]=C(C2=O)C1=[C][C]=C([C]=[C]1)O)O)O)[C][C]C([C])([C])O	-8.1
**5353357**	O1C(=[C]C(=O)C2=[C]C(=C([C]=C12)O)O)C1=[C][C]=[C][C]=[C]1	-8.1
**5321861**	o1c(c(c(=O)c2=C([C]=C(C(=c12)O)O)O)O[C])C1=[C]C(=C([C]=[C]1)O[C])O	-8.1
**5320438**	O1C(=[C]C(=O)C2=C(C(=C([C]=C12)O)O[C])O)C1=[C][C]=C([C]=[C]1)O[C]	-8.1
**5319422**	O1[C]=C(C(=O)C2=C1[C]=C([C]=[C]2)O)C1=[C]C(=C([C]=[C]1)O)O[C]	-8.1
**5318869**	o1c(c(c(=O)c2C(=[C]C(=[C]c12)O[C])O)O[C])C1=[C][C]=C([C]=[C]1)O	-8.1
**5284648**	O1[C]=C(C(=O)C2=C1[C]=C([C]=[C]2)O)C1=[C]C(=C([C]=[C]1)O)O	-8.1
**5282074**	O1[C]=C(C(=O)C2=C1[C]=C([C]=C2O)O)C1=[C][C]=C([C]=C1O)O	-8.1
**5281950**	o1c(c(c(=O)c2=C([C]=C(C(=c12)C([C])([C])[C]=[C])O)O)O)C1=[C][C]=[C][C]=[C]1	-8.1
**5281949**	o1c(c(c(=O)c2C(=[C]C(=C(c12)C([C])([C])[C]=[C])O)O)O)C1=[C][C]=C([C]=[C]1)O[C]	-8.1
**5281811**	O1[C]=C(C(=O)C2=C(C(=C([C]=C12)O)O[C])O)C1=[C][C]=C([C]=[C]1)O	-8.1
**5281691**	o1c(c(c(=O)c2C(=[C]C(=[C]c12)O[C])O)O)C1=[C][C]=C(C(=[C]1)O)O	-8.1
**5281628**	O1C(=[C]C(=O)C2=C1[C]=C(C(=C2O)O[C])O)C1=[C][C]=C([C]=[C]1)O	-8.1
**5281617**	O1C(=[C]C(=O)C2=C1[C]=C([C]=C2O)O[C])C1=[C][C]=C([C]=[C]1)O	-8.1
**5281611**	o1c2=[C]C(=[C][C]=c2c(=O)c(c1C1=[C][C]=C([C]=[C]1)O)O)O	-8.1
**5280961**	O1[C]=C(C(=O)C2=C1[C]=C([C]=C2O)O)C1=[C][C]=C([C]=[C]1)O	-8.1
**5280448**	O1[C]=C(C(=O)C2=C1[C]=C([C]=[C]2)O)C1=[C][C]=C(C(=[C]1)O)O[C]	-8.1
**5280443**	O1C(=[C]C(=O)C2=C1[C]=C([C]=C2O)O)C1=[C][C]=C([C]=[C]1)O	-8.1
**5280417**	o1c(c(c(=O)c2=C([C]=C([C]=c12)O[C])O)O[C])C1=[C]C(=C([C]=[C]1)O)O	-8.1
**4231835**	O1C2=[C][C]=[C][C]=C2C(=O)[C]=C1C1=C([C]=[C]C(=[C]1)O[C])O[C]	-8.1
**3083783**	O1C(=[C]C(=O)c2c(c(c(c(c12)O)O[C])O[C])O)C1=[C][C]=C([C]=[C]1)O[C]	-8.1
**1232440**	O1C2=[C][C]=[C][C]=C2[C][C][C]1C1=[C][C]=[C][C]=[C]1	-8.1
**847733**	O1C2=[C][C]=[C][C]=C2[C][C]([C]1C1=[C][C]=[C][C]=[C]1)O	-8.1
**689014**	O1C(=[C]C(=O)C2=C1C(=C([C]=[C]2)O[C])O[C])C1=[C][C]=[C][C]=[C]1	-8.1

**688715**	o1c2=[C][C]=[C][C]=c2c(=O)c(c1C1=[C][C]=C([C]=[C]1)O)O	-8.1
**688671**	O1C(=[C]C(=O)C2=[C][C]=C([C]=C12)O[C])C1=C([C]=[C][C]=[C]1)O[C]	-8.1
**688659**	o1c(c(c(=O)c2[C]=C([C]=[C]c12)O)O)C1=[C][C]=[C][C]=[C]1	-8.1
**676295**	o1c2=[C][C]=[C][C]=c2c(=O)c(c1C1=[C][C]=[C]C(=[C]1)O)O	-8.1
**676290**	O1C2=[C][C]=[C][C]=C2C(=O)[C]=C1C1=C([C]=C([C]=[C]1)O)O	-8.1
**676289**	O1C2=C([C]=[C][C]=[C]2)C(=O)[C]=C1C1=C(C(=[C][C]=[C]1)O)O	-8.1
**636496**	O1C2=[C]C3=C(C(=O)C(=[C]O3)C3=C([C]=C([C]=[C]3)O[C])O)C(=C2[C]=[C]C1([C])[C])O[C]	-8.1
**633124**	O1C(=[C]C(=O)c2c(c(c(c(c12)O[C])O)O[C])O)C1=[C]C(=C(C(=[C]1)O[C])O[C])O	-8.1
**578729**	o1c2=[C][C]=[C][C]=c2c(=O)c(c1C1=[C][C]=[C][C]=C1O[C])O	-8.1
**468911**	O1C2=C(C(=[C]C(=C2O)O)O)C(=O)[C]([C]1C1=[C][C]=C([C]=[C]1)O)O	-8.1
**443638**	O1C2=C(C(=[C]C(=[C]2)O)O)[C]([C][C]1C1=[C][C]=C([C]=[C]1)O)O	-8.1
**369616**	O1C2=C([C]=C([C]=C2O[C])[C][C]=[C])[C]([C][C]1C1=[C][C]=C(C(=[C]1)O[C])O[C])OC(=O)[C]	-8.1
**369615**	O1C2=C([C]=[C][C]=C2O[C])C(=[C])[C][C]1C1=[C]C(=C([C]=[C]1)O)O[C]	-8.1
**321346**	O1C2=[C]C(=[C]C(=C2C(=O)[C][C]1C1=[C][C]=C([C]=[C]1)O[C])O)O[C]	-8.1
**161271**	O1C(=[C]C(=O)C2=C1[C]=C(C(=C2O)O[C])O[C])C1=[C][C]=C([C]=[C]1)O[C]	-8.1
**154227**	O1C2=C([C]=[C]C(=[C]2)O[C])C(=O)[C]=C1C1=[C][C]=C(C(=[C]1)O[C])O[C]	-8.1
**94156**	O1C2=[C][C]=[C][C]=C2[C][C][C]1C1=[C][C]=[C][C]=[C]1	-8.1
**73202**	O1C2=C(C(=[C]C(=[C]2)O)O)C(=O)[C]([C]1C1=[C][C]=[C][C]=[C]1)O	-8.1
**72304**	O1[C]=C(C(=O)C2=C1[C]=[C][C]=[C]2)C1=[C][C]=[C][C]=[C]1	-8.1
**55748**	O1C(=[C]C(=O)C2=[C][C]=[C]C(=C12)[C]C(=O)O)C1=[C][C]=[C][C]=[C]1	-8.1
**53645**	O1C(=[C]C(=O)C2=[C][C]=[C]C(=C12)O)C1=[C][C]=[C][C]=[C]1	-8.1
**25585**	o1c([C])c(c(=O)c2=[C][C]=[C]C(=c12)O[C][C]N([C])[C])C1=[C][C]=C([C]=[C]1)O[C]	-8.1
**20399**	O1C2=[C]C(=[C][C]=C2C(=O)[C]([C]1C1=[C]C(=C(C(=[C]1)O)O)O)O)O	-8.1
**10680**	O1C2=C([C]=[C][C]=[C]2)C(=O)[C]=C1C1=[C][C]=[C][C]=[C]1	-8.1
**139030757**	O1[C]=C(C(=O)C2=C1C(=C([C]=[C]2)[C][C][C]O)O[C])C1=[C][C]=C([C]=[C]1)O[C]	-8
**134822278**	O1[C]=C(C(=O)C2=C(C3=C([C]=C12)O[C]O3)O)C1=[C][C]=C([C]=[C]1)O[C]	-8

**129864635**	O1C(=[C]C(=O)C2=C1[C]=[C][C]=C2O[C]O)C1=[C][C]=[C][C]=[C]1	-8
**129847910**	O1C2=C(C(=[C]C(=[C]2)O)O)[C](C(=O)[C]1C1=[C][C]=[C]C(=[C]1)O[C])O	-8
**129847909**	o1c(c(c(=O)c2=[C][C]=C([C]=c12)O[C](O)O)O[C]O)C1=[C][C]=[C][C]=[C]1	-8
**129821705**	o1c(c([C])c(=O)c2[C]=C(C(=C(c12)N([C])[C])O)O)C1=[C][C]=C([C]=[C]1)O	-8
**101665962**	o1c2=[C]C(=[C][C]=c2c(=O)c(c1C1=[C][C]=C(C(=[C]1)O[C][C]O)O[C][C]O)O[C][C]O)O[C][C]O	-8
**101028409**	O1C(=[C]C(=O)C2=C1C(=C([C]=C2O[C])O[C])N[C][C]=[C])C1=[C][C]=[C][C]=[C]1	-8
**101028406**	O1C(=[C]C(=O)C2=C1C(=C([C]=C2O[C])O[C])N[C][C][C])C1=[C][C]=[C][C]=[C]1	-8
**100943379**	C1(=[C][C]=C([C]=[C]1)C1=[C]C(=O)C2=[C][C]=[C][C]=C2O1)S(=O)(=O)N([C][C])[C][C]	-8
**86606987**	C1(=[C][C]=C2C(=O)[C]=C(OC2=[C]1)C1=[C]C(=C(C(=[C]1)O[C])O[C])O[C])O[C][C]1O[C]1	-8
**86083806**	O1[C]2[C]=[C][C]=[C]C2=[C][C]=C1C1=[C][C]=C([C]=[C]1)NC(=O)[C]	-8
**73265447**	C1(=[C]OC2=C([C]=[C]C(=C2[C])O)C1=O)C1=[C][C]=C([C]=[C]1)C(F)(F)F	-8
**70696494**	O1C2=[C]C(=[C]C(=C2C(=O)[C][C]1C1=[C]C(=[C]C(=[C]1)O)O)O)O[C]	-8
**66968868**	C1(=[C][C]=c2c(=[C]1)c(=O)c(c(o2)C1=[C][C]=C([C]=[C]1)N([C][C])[C][C])O)[C]Br	-8
**54073202**	C1(=[C]C2=C(C(=[C]1)O)C(=O)[C][C](O2)C1=[C]C(=C([C]=[C]1)O[C])O)O[C][C]Br	-8
**49788611**	o1c(c(c(=O)c2=C([C]=C(C(=c12)O[C])O[C])O)O)C1=[C]C2=C([C]=[C]1)O[C]O2	-8
**44260080**	o1c(c(c(=O)c2c(c3c(c(c12)O[C])O[C]O3)O[C])O[C])C1=[C]C2=C(C(=[C]1)O[C])O[C]O2	-8
**25232840**	O1C2=C(C(=[C]C(=[C]2)O)O)[C][C][C]1C1=[C][C]=C([C]=[C]1)O[C]	-8
**24721178**	o1c(c(c(=O)c2[C]=[C]C(=C(c12)O)O)O)C1=[C][C]=[C][C]=C1O	-8
**23724666**	C1(=[C]OC2=[C]C(=[C][C]=C2C1=O)O)C1=[C]C2=C(O[C]O2)[C]=C1O	-8
**21291576**	O1[C]=C(C(=O)C2=C([C]=C([C])[C]=C12)O)C1=[C][C]=C([C]=[C]1)O	-8
**20394006**	o1c([C])c(c(=O)c2=C([C]=C([C]=c12)OC(=O)[C])OC(=O)[C])C1=[C][C]=[C][C]=[C]1	-8
**18702583**	O1C(=[C]C(=O)C2=C(C(=[C][C]=C12)O[C])O)C1=[C]C(=C([C]=[C]1)O)O	-8

**18324974**	c1(c(=O)c2=[C][C]=[C][C]=c2oc1C1=[C][C]=C([C]=[C]1)S(=O)(=O)[C])C1=[C][C]=C(Cl)[C]=[C]1	-8
**18324902**	c1(c(c(=O)c2=[C][C]=[C][C]=c2o1)C1=[C][C]=C([C]=[C]1)F)C1=[C][C]=C([C]=[C]1)S(=O)(=O)[C]	-8
**16213663**	O1C(=[C]C(=C2[C]=[C]C(=[C][C]12)N([C][C])[C][C])C1=C([C]=[C][C]=[C]1)C(=O)O)C1=[C][C]=C([C]=[C]1)N([C])[C]	-8
**14886014**	o1c2=C(C(=[C]C(=c2c(=O)c(c1C1=[C]C2=C([C]=[C]1)O[C]O2)O[C])O[C])O)O[C]	-8
**14791392**	O1C2=C([C]=[C]C(=[C]2)O)C(=O)[C]=C1C1=[C][C]=C([C]=[C]1)N	-8
**14730798**	O1C2=[C]C(=[C][C]=C2C(=O)[C]([C]1C1=[C][C]=[C][C]=[C]1)O)O	-8
**14704648**	o1c(c(c(=O)c2C(=C3C(=[C]c12)O[C]O3)O[C])O[C])C1=[C]C(=C2C(=[C]1)O[C]O2)O[C]	-8
**14440496**	o1c2[C]=C([C]=[C]c2c(=O)c(c1C1=C([C]=[C][C]=[C]1)O[C])O)O[C]	-8
**13889022**	O1C(=[C]C(=O)C2=C1C(=C([C]=C2O)O[C])O[C])C1=C([C]=[C][C]=C1O[C])O	-8
**13873818**	O1C(=[C]C(=O)C2=C(C(=C([C]=C12)O[C]C1=[C][C]=[C][C]=[C]1)O[C])O[C])C1=[C]C(=C([C]=[C]1)O[C])O[C]	-8
**12444947**	C1(=[C]C2=C(C(=O)C(=[C]O2)C2=[C][C]=C([C]=[C]2)O[C])[C]=C1O[C])O[C]1O[C]([C]([C]([C]1O)O)O)[C]O	-8
**12318033**	O1C2=[C][C]=[C][C]=C2[C]C(=O)[C]1C1=[C][C]=[C][C]=[C]1	-8
**12310757**	C1(=C([C]=C(c2c(=O)c(c(oc12)C1=[C][C]=C([C]=[C]1)O[C])O)O)O)[C][C]C([C])([C])O	-8
**11777425**	O1C(=[C]C(=O)C2=[C]C(=[C][C]=C12)[C]C#N)C1=[C][C]=[C][C]=[C]1	-8
**11609345**	O1C(=[C]C(=O)c2c(c(c(c(c12)O[C])O)O[C])O)C1=[C][C]=[C][C]=[C]1	-8
**11289628**	O1C2=[C]C(=[C]C(=C2C(=O)[C][C]1C1=[C]C(=[C]C(=[C]1)O)O)O)O[C]	-8
**10759582**	O1C2=[C]C(=C(C(=C2C(=O)[C][C]1C1=[C][C]=C([C]=[C]1)O)O)C([C])([C])[C]=[C])O	-8
**10643413**	Brc1c(=O)c2=[C][C]=[C][C]=c2oc1C1=[C][C]=C([C]=[C]1)S(=O)(=O)[C]	-8
**10542374**	O1[C]=C(C(=O)C2=C1C(=C([C]=C2O)O)O)C1=[C][C]=C(C(=[C]1)O)O	-8
**10491973**	O1C(=[C]C(=O)C2=C1[C]=[C][C]=C2O[C][C])C1=[C][C]=[C][C]=[C]1	-8
**9864504**	c1(c(c(=O)c2=[C][C]=[C][C]=c2o1)C1=[C][C]=[C][C]=[C]1)C1=[C][C]=C([C]=[C]1)S(=O)(=O)N	-8
**6028923**	C1(=[C]C(=O)C2=[C][C]=C([C]=C2O1)O)C1=[C][C]=C(Br)[C]=[C]1	-8
**5748553**	o1c(c(c(=O)c2=C([C]=C(C(=c12)O[C])O)O)O[C])C1=[C]C(=C([C]=[C]1)O)O	-8
**5492944**	O1[C]=C(C(=O)C2=C1C(=C([C]=C2O)O)O)C1=[C][C]=C([C]=[C]1)O	-8
**5481647**	O1C(=[C]C(=O)C2=C(C(=C([C]=C12)O)O[C])O[C])C1=[C][C]=[C][C]=[C]1	-8
**5468257**	O1C2=[C][C]=[C][C]=C2[C]([C][C]1C1=[C][C]=[C][C]=[C]1)[N]N	-8
**5395695**	o1c([C])c(c(=O)c2=C([C]=C([C]=c12)O)O)C1=[C][C]=[C][C]=[C]1	-8
**5321538**	O1C2=C([C]=C(C(=[C]2)O)O[C])C(=O)[C][C]1C1=[C]C(=C([C]=[C]1)O)O	-8
**5317747**	O1C2=[C]C(=[C]C(=C2C(=O)[C]([C]1C1=[C][C]=[C][C]=[C]1)O)O)O[C]	-8

**5317652**	O1C2=[C][C]=C(C(=C2[C]=[C]C1([C])[C])O)C1=[C]OC2=C([C]=[C]C(=[C]2)O)C1=O	-8
**5284649**	O1[C]=C(C(=O)C2=[C]C(=C([C]=C12)O)O)C1=[C][C]=C([C]=[C]1)O	-8
**5281607**	O1C(=[C]C(=O)C2=C([C]=C([C]=C12)O)O)C1=[C][C]=[C][C]=[C]1	-8
**5281603**	o1c(c(c(=O)c2=C(C(=C([C]=c12)O)O[C])O)O[C])C1=[C][C]=C(C(=[C]1)O)O	-8
**5280681**	o1c2=[C]C(=[C]C(=c2c(=O)c(c1C1=[C]C(=C([C]=[C]1)O)O)O[C])O)O	-8
**5280362**	c1(c(=O)c2C(=[C]C(=[C]c2oc1C1=[C]C(=C([C]=[C]1)O)O)O)O)OS([O])([O])O	-8
**5280343**	o1c(c(c(=O)c2C(=[C]C(=[C]c12)O)O)O)C1=[C][C]=C(C(=[C]1)O)O	-8
**5106787**	O1C2=C([C]=C([C]=[C]2)O)C(=O)[C][C]1C1=[C][C]=[C][C]=C1O	-8
**4872981**	O1C2=C(C(=[C]C(=[C]2)O[C])O)C(=O)[C][C]1C1=[C]C(=C([C]=[C]1)O)O	-8
**4183640**	C1(=[C]C2=C([C]=[C]1)C(=O)C(=[C]O2)C1=[C][C]=C([C]=[C]1)O)O[C]1O[C]([C]([C]([C]1O)O)O)[C]O	-8
**4169706**	FC1=[C]C2=C([C]=[C]1)O[C]([C]C2=O)C1=[C][C]=[C][C]=[C]1	-8
**3070245**	c1(c([C])c(=O)c2[C]=[C]C(=C(c2o1)[C]N([C])[C])O[C])C1=[C][C]=C(Cl)[C]=[C]1	-8
**911486**	o1c2=[C]C(=[C][C]=c2c(=O)c(c1[C])C1=[C][C]=C([C]=[C]1)N(=O)=O)O[C]	-8
**688836**	o1c(c(c(=O)c2=[C][C]=C([C]=c12)O[C])O)C1=[C]C(=[C][C]=[C]1)O[C]	-8
**555407**	o1c(nc(=O)c2=[C][C]=[C][C]=c12)C1=[C][C]=[C][C]=[C]1	-8
**471706**	O1[C]=C(C(=O)C2=[C]C(=C([C]=C12)OC(=O)[C])OC(=O)[C])C1=[C][C]=C([C]=[C]1)OC(=O)[C]	-8
**466277**	BrC1=[C][C]=C2C(=O)[C]=C(OC2=[C]1)C1=[C][C]=[C][C]=[C]1	-8
**462691**	O1C2=C([C]=[C][C]=C2C(=O)[C][C]1C1=[C][C]=[C][C]=[C]1)[C]C(=O)O	-8
**455313**	o1c2[C]=[C][C]=[C]c2c(=O)c(c1C1=C([C]=[C][C]=[C]1)O)O	-8
**369599**	O1C(=[C]C(=O)C2=C(C(=C([C]=C12)O[C])[C])O)C1=[C][C]=[C][C]=[C]1	-8
**348130**	O1C2=[C]C(=[C]C(=C2C(=O)[C][C]1C1=[C][C]=C([C]=[C]1)O)O)O[C]	-8
**250312**	O1C(=[C]C(=C2[C][C]=[C][C]=C12)[N]O)C1=[C][C]=[C][C]=[C]1	-8
**213728**	O1C(=[C]C(=O)C2=C1C(=[C][C]=[C]2)O[C])C1=[C][C]=[C][C]=[C]1	-8
**194690**	O1C(=[C]C(=O)C2=[C]C(=[C]C(=C12)O[C])O[C])C1=[C][C]=[C][C]=[C]1	-8
**182026**	O1C2=C(C(=C(C(=[C]2)O)[C])O)C(=O)[C]([C]1C1=[C]C(=C([C]=[C]1)O)O)O	-8
**161860**	O1C2=C([C]=[C][C]=[C]2)C(=O)[C]=C1C1=[C][C]=[C][C]=C1O	-8
**147157**	O1C(=[C]C(=O)C2=[C]C(=[C][C]=C12)O[C])C1=[C][C]=[C][C]=[C]1	-8
**97214**	O1C(=[C]C(=O)C2=C1[C]=C(C(=C2O)O[C])O[C])C1=[C][C]=C(C(=[C]1)O)O[C]	-8
**88881**	O1C(=[C]C(=O)C2=C1[C]=C([C]=C2O[C])O[C])C1=[C][C]=[C][C]=[C]1	-8

**73571**	O1C2=[C]C(=[C]C(=C2C(=O)[C][C]1C1=[C][C]=C([C]=[C]1)O)O)O[C]	-8
**11349**	o1c2=[C][C]=[C][C]=c2c(=O)c(c1C1=[C][C]=[C][C]=[C]1)O	-8
**10185**	O1C2=[C]C(=[C]C(=C2C(=O)[C]([C]1C1=[C][C]=C(C(=[C]1)O)O)O)O)O	-8
**1686**	C1(=[C]C(=O)C2=[C][C]=[C][C]=C2O1)C1=[C][C]=C(Br)[C]=[C]1	-8
**135121498**	c1(c(c(=O)c2C(=[C][C]=[C]c2o1)O)O)C1=[C]C(=C2C(=[C]1)[C]2)O[C]	-7.9
**129844531**	O1C2=[C]C(=[C]C(=C2C(=O)[C@@]([C])([C]1C1=[C][C]=C(C(=[C]1)[C])O)O)O)O	-7.9
**129822390**	O1C(=[C]C(=O)c2c(c(c(c(c12)N(=O)=O)OC(=O)[C])OC(=O)[C])OC(=O)[C])C1=[C][C]=[C][C]=[C]1	-7.9
**129787765**	O1C(=[C]C(=C2[C][C]=[C][C]=C12)[N]N)C1=[C][C]=[C][C]=[C]1	-7.9
**129734164**	o1c(c(c(=O)c2c(c(c(c12)O)O[C])O[C])O[C])O)C1=[C][C]=[C][C]=C1O	-7.9
**129728743**	c1(c(c(=O)c2=[C][C]=[C][C]=c2o1)C1=[C][C]=[C][C]=[C]1)[C]1O[C]([C]([C]([C]1O)O)O)[C]O	-7.9
**129716149**	C1(=C([C]=C2C(=C1O)C(=O)C(=[C]O2)C1=[C][C]=C([C]=[C]1)O)O)[C]([C]1O[C]1)[C][C]=C([C])[C]	-7.9
**129704118**	O1C(=[C]C(=O)C2=C([C]=C([C]=C12)O[C]O)O[C]O)C1=[C][C]=[C][C]=[C]1	-7.9
**129670476**	O1C2=C([C])[C]=[C]C(=C2C(=O)[C][C]1C1=[C][C]=[C][C]=[C]1)[C]O	-7.9
**101740538**	C1(=[C]C2=C(C(=O)[C]=C(O2)C2=[C][C]=[C][C]=[C]2)C(=C1O[C])O)OS([O])([O])O	-7.9
**101214937**	o1c(c(c(=O)c2[C]=C([C]=[C]c12)[C]N([C][C])[C][C])O)C1=[C][C]=C([C]=[C]1)N([C][C])[C][C]	-7.9
**86641845**	O1C(=[C]C(=O)C2=C1[C]=C([C]=C2O)O[C]C#[C])C1=[C][C]=C(C(=[C]1)O[C])O[C]	-7.9
**86236608**	C1(=[C]C2=C([C]=[C]1)C(=O)[C]=C(O2)C1=[C]C(=[C]C(=[C]1)O[C])O[C])O[C][C]1O[C]1	-7.9
**69033169**	C1(=[C]C2=C([C]=[C]1)C(=O)[C]=C(O2)C1=[C]C(=C(C(=[C]1)O[C])O[C])O[C])O[C][C]Br	-7.9
**69030799**	C1(=[C]C2=C([C]=[C]1)C(=O)[C]=C(O2)C1=[C]C(=C(C(=[C]1)O[C])O[C])O[C])O[C][C][C]Br	-7.9

**69030155**	C1(=[C][C]=C2C(=O)[C]=C(OC2=[C]1)C1=[C]C(=[C]C(=[C]1)O[C])O[C])O[C][C][C]Br	-7.9
**56649088**	O1C(=[C]C(=O)C2=C(C(=C([C]=C12)O[C][C]=[C])O)O)C1=[C][C]=[C][C]=[C]1	-7.9
**25073757**	O1C2=[C]C(=[C]C(=C2C(=O)[C][C]1C1=C([C]=[C]C(=[C]1)O)O)O)O	-7.9
**22134047**	o1c(c(c(=O)c2C(=C([C]=[C]c12)O[C])O[C])O)C1=[C][C]=[C][C]=[C]1	-7.9
**21673114**	O1C(=[C]C(=O)C2=C1[C]=C([C]=C2O)O[C]O[C])C1=[C][C]=[C][C]=[C]1	-7.9
**17585058**	C1(=[C][C]=C2C(=O)C(=[C]OC2=[C]1)C1=[C][C]=[C][C]=[C]1)O[C][C]Br	-7.9
**14504257**	C1(=C(C(=[C]C(=C1C1=[C]OC2=C([C]=[C]C(=[C]2)O)C1=O)O)O)O[C])[C][C]C([C])([C])O	-7.9
**13942548**	o1c(c(c(=O)c2c(c3c(c(c12)O[C])O[C]O3)O[C])O[C])C1=[C]C2=C([C]=[C]1)O[C]O2	-7.9
**13886894**	O1C2=[C]C(=[C][C]=C2[C]([C]([C]1C1=[C][C]=C([C]=[C]1)O)O)O)O	-7.9
**13568446**	o1c2[C]=[C][C]=[C]c2c(=O)c(c1C1=[C][C]=[C][C]=[C]1)C(=O)[C]	-7.9
**12000157**	O1C2=[C]C(=[C]C(=C2C(=O)[C][C]1C1=[C][C]=C([C]=C1O)O)O)O[C]	-7.9
**11499970**	O1C2=C([C]=[C][C]=[C]2)C(=O)[C][C]1C1=[C][C]=C([C]=[C]1)N([C])[C]	-7.9
**11033582**	O1C2=C(C(=[C][C]=C2[C][C]([C]1C1=[C][C]=C(C(=[C]1)O)O)O)O)O	-7.9
**10761665**	O1C2=[C]C(=C(C(=C2C(=O)[C][C]1C1=[C][C]=C(C(=[C]1)O[C])O)O)C([C])([C])[C]=[C])O	-7.9
**10468234**	O1C2=[C][C]=[C][C]=C2C(=O)[C@@]([C])([C]1C1=[C][C]=C([C])[C]=[C]1)O	-7.9
**10315196**	O1C2=C(C(=C(C(=[C]2)O)C([C])([C])[C]=[C])O)C(=O)[C][C]1C1=[C][C]=C([C]=[C]1)O	-7.9
**5487855**	o1c2[C]=C([C]=C(c2c(=O)c(c1C1=[C][C]=C(C(=[C]1)O[C])O[C])O)O)O	-7.9
**5385091**	C1(=[C]OC2=C([C]=[C]C(=[C]2)O)C1=O)C1=[C]C2=C(O[C]O2)[C]=C1O[C]	-7.9
**5380976**	o1c2=[C]C(=[C][C]=c2c(=O)c(c1[C])C1=[C][C]=[C][C]=[C]1)O	-7.9
**5378260**	O1[C]=C(C(=O)C2=C1C(=C([C]=[C]2)O)O)C1=[C][C]=C([C]=C1O)O	-7.9
**5362017**	o1c(c(c(=O)c2C(=[C]C(=C(c12)O)O)O)O)C1=[C][C]=[C][C]=[C]1	-7.9
**5360930**	o1c(c(c(=O)c2=C([C]=C(C(=c12)O)O)O)O[C])C1=[C][C]=[C][C]=[C]1	-7.9
**5351234**	O1C2=C(C(=[C]C(=[C]2)O)O)C(=O)[C]=C1C1=[C]C(=C([C]=[C]1)O[C])O[C]	-7.9
**5322009**	O1C(=[C]C(=O)C2=C1[C]=C(C(=C2OC(=O)[C])O[C])OC(=O)[C])C1=[C][C]=C([C]=[C]1)OC(=O)[C]	-7.9
**5320287**	o1c(c(c(=O)c2=C([C]=C([C]=c12)O[C])O)O)C1=[C]C(=C([C]=[C]1)O[C])O	-7.9
**5316653**	O1[C]=C(C(=O)C2=C1[C]=C1C(=C2O)O[C]O1)C1=[C]C(=C(C(=[C]1)O)O[C])O[C]	-7.9
**5315202**	O1C(=[C]C(=O)C2=C1[C]=C([C]=C2O[C])O)C1=[C][C]=C([C]=[C]1)O	-7.9
**5282154**	o1c2=[C]C(=[C]C(=c2c(=O)c(c1C1=[C]C(=C(C(=[C]1)O)O)O[C])O)O)O	-7.9
**5281804**	O1[C]=C(C(=O)C2=C1[C]=C([C]=C2O)O[C])C1=[C][C]=C([C]=[C]1)O	-7.9

**5281801**	O1[C]=C(C(=O)C2=C([C]=C([C]=C12)O)O)C1=[C][C]=C(C(=[C]1)O)O	-7.9
**5281779**	O1[C]=C(C(=O)C2=C(C3=C([C]=C12)O[C]O3)O)C1=[C][C]=C([C]=[C]1)O	-7.9
**5281708**	O1[C]=C(C(=O)C2=C1[C]=C([C]=[C]2)O)C1=[C][C]=C([C]=[C]1)O	-7.9
**5281702**	O1C2=C(C(=[C]C(=[C]2)O)O)C(=O)[C]=C1C1=[C]C(=C(C(=[C]1)O[C])O)O[C]	-7.9
**5281697**	O1C(=[C]C(=O)C2=C1[C]=C(C(=C2O)O)O)C1=[C][C]=C([C]=[C]1)O	-7.9
**5280378**	O1[C]=C(C(=O)C2=C1[C]=C([C]=[C]2)O)C1=[C][C]=C([C]=[C]1)O[C]	-7.9
**5271991**	O1C(=[C]C(=O)C2=C1C(=C([C]=C2O)O)O[C])C1=C(C(=[C][C]=C1O)O)O[C]	-7.9
**3080750**	O1C(=[C]C(=O)c2c(c(c(c(c12)O[C])O[C])O[C])O)C1=[C][C]=C(C(=[C]1)O)O[C]	-7.9
**3070241**	c1(c([C])c(=O)c2=[C][C]=C(C(=c2o1)[C]N([C][C])[C][C])O[C])C1=[C][C]=C(F)[C]=[C]1	-7.9
**688827**	o1c2=[C][C]=[C][C]=c2c(=O)c(c1C1=[C]C(=C(C(=[C]1)O[C])O[C])O[C])O	-7.9
**586090**	O1C2=C(C(=[C][C]=[C]2)O)C(=O)[C][C]1C1=[C][C]=[C][C]=[C]1	-7.9
**513103**	O1C2=[C][C]=[C][C]=C2C(=O)[C]([C]1)[C]C1=[C][C]=[C][C]=[C]1	-7.9
**439533**	O1C2=C(C(=[C]C(=[C]2)O)O)C(=O)[C]([C]1C1=[C]C(=C([C]=[C]1)O)O)O	-7.9
**369608**	O1C2=C([C]=C([C]=C2[C]([C][C]1C1=[C][C]=[C][C]=[C]1)O)[C][C]=[C])O[C]	-7.9
**265699**	o1c(c(c(=O)c2[C]=[C]C(=[C]c12)O[C])O)C1=[C][C]=C([C]=[C]1)O[C]	-7.9
**125540**	ClC1=C2C(=C([C]=C1O)O)C(=O)[C]([C])[C](O2)C1=[C][C]=C([C]=[C]1)O	-7.9
**125526**	O1C2=C(C(=[C]C(=[C]2)O)O)C(=O)[C]([C])[C]1C1=[C][C]=C([C]=[C]1)O	-7.9
**68112**	O1C(=[C]C(=O)C2=C1[C]=[C][C]=C2O)C1=[C][C]=[C][C]=[C]1	-7.9
**31161**	O1C(=[C]C(=O)C2=C1[C]=C(C(=C2O)O)O[C])C1=[C][C]=C(C(=[C]1)O)O	-7.9
**3078**	o1c(c([C])c(=O)c2[C]=[C]C(=C(c12)[C]N([C])[C])O[C])C1=[C][C]=[C][C]=[C]1	-7.9
**132517368**	O1C(=[C]C(=O)c2c(c([C])c(c(c12)C([C])([C])O)O[C])O)C1=[C][C]=C([C]=[C]1)O[C]	-7.8
**132279663**	O1[C]=C(C(=O)C2=C1[C]=C(C(=C2O[C])O[C])O)C1=C2C(=[C][C]=[C]1)O[C]O2	-7.8
**129887481**	o1c(c(c(=O)c2C(=C([C]=[C]c12)O)O[C])C1=[C][C]=[C][C]=[C]1)O[C]	-7.8
**129847959**	O1C2=C([C]=[C]C(=C2O)O)[C]([C]([C]1C1=[C][C]=C([C]=[C]1)O)O)O	-7.8

**129837195**	o1c(c(c(=O)c2c(c(c(c12)O)O)O)O[C])O[C])C1=[C][C]=[C][C]=C1O	-7.8
**129824510**	O1C(=[C]C(=O)C2=C1C(=C([C]=C2O[C])O[C])C1=[C]C(=C(C(=[C]1)O[C])O[C])O[C])C1=[C][C]=[C][C]=[C]1	-7.8
**129711222**	O1C2=C([C]C(=O)[C]=[C]2)[C][C][C]1C1=[C][C]=[C][C]=[C]1	-7.8
**102320039**	O1[C]=C(C(=O)c2c(c(c(c12)O)O)O[C])O)C1=[C]C(=C([C]=[C]1)O)O[C]	-7.8
**102145698**	O1C2=C([C]=C(C(=[C]2)O[C])O)C(=O)[C][C]1C1=[C][C]=C(C(=[C]1)O)O	-7.8
**101890693**	O1c2c([C])c(c([C])c(c2C(=O)[C][C]1C1=[C][C]=[C][C]=[C]1)O[C])O	-7.8
**101624373**	O1C2=[C]C(=[C][C]=C2[C]([C][C]1C1=[C][C]=[C][C]=[C]1)O)O[C]	-7.8
**101591281**	C1(=C(c2c(C(=[C]1)O[C])c(=O)c(c(o2)C1=[C][C]=C(C(=[C]1)O[C])O)O[C])O[C])O[C][C][C][C][C]	-7.8
**101584232**	O1C2=C(C(=[C]C(=C2C(=O)[C][C]1C1=[C][C]=[C][C]=[C]1)O[C])O[C])/[C]=[C]/C(=[C])[C]	-7.8
**71586772**	O1C(=[C]C(=O)C2=C(C(=C([C]=C12)OC(=O)[C])O[C])OC(=O)[C])C1=[C][C]=[C][C]=[C]1	-7.8
**56648912**	O1C(=[C]C(=O)C2=C1[C]=C(C(=C2OC(=O)[C])OC(=O)[C])O[C][C]=[C])C1=[C][C]=[C][C]=[C]1	-7.8
**54728923**	o1c(=O)c(c(c2C(=[C]C(=[C]c12)O)O)O)C1=[C][C]=C([C]=[C]1)O	-7.8
**53796934**	O1[C]=C(C(=O)C2=C([C]=C([C]=C12)O[C])O)C1=[C][C]=C([C]=C1O)O[C]	-7.8
**21315732**	O1C2=C([C]=C([C]=[C]2)C(=O)O[C][C])C(=O)[C]=C1C1=[C]C(=[C][C]=C1O[C]([C])[C])[C]	-7.8
**19972541**	o1c(c([C])c(=O)c2[C]=[C][C]=C(c12)C(=O)O[C][C])C1=[C][C]=[C][C]=[C]1	-7.8
**15761525**	O1C2=[C][C]=[C][C]=C2C(=O)C([C]1C1=[C][C]=[C][C]=[C]1)([C]O)[C]O	-7.8
**15126657**	O1[C]=C(C(=O)C2=C1C(=C([C]=[C]2)O)[C])C1=[C][C]=C([C]=[C]1)O	-7.8
**14057034**	O1C2=C(C3=C(C(=[C]2)O)C(=O)C(=[C]O3)C2=[C]C(=C([C]=C2O[C])O)O[C])[C]=[C]C1([C])[C]	-7.8
**13916267**	o1c(c(c(=O)c2=C([C]=C(C(=c12)O[C])O)O[C])O[C])C1=[C]C(=C([C]=[C]1)O[C])O[C]	-7.8
**11772232**	O1C(=[C]C(=O)C2=C1[C]=C([C]=C2O)O[C]O[C][C]O[C])C1=[C][C]=[C][C]=[C]1	-7.8
**10755200**	O1C2=C([C]=C([C]=[C]2)N)C(=O)[C]=C1C1=[C][C]=C([C]=[C]1)N([C])[C]	-7.8

**10422310**	O1C(=[C]C(=O)C2=C1[C]=[C][C]=C2N)C1=[C][C]=C([C]=[C]1)N	-7.8
**7454026**	O1[C]=C(C(=O)C2=[C][C]=C([C]=C12)O)C1=[C][C]=C([C]=[C]1)N(=O)=O	-7.8
**6253276**	C1(=C(C(=[C]C(=C1C(=O)/[C]=[C]/C1=[C][C]=C([C]=[C]1)O[C])O)O[C])O[C])O[C]	-7.8
**5748605**	O1[C]=C(C(=O)C2=C1[C]=C([C]=[C]2)O)C1=[C]C(=C([C]=[C]1)O[C])O[C]	-7.8
**5491412**	o1c2[C]=C([C]=C(c2c(=O)c(c1C1=[C]C(=C(C(=[C]1)O)O[C])O)O[C])O)O[C]	-7.8
**5466136**	o1c2[C]=C(C(=C(c2c(=O)c(c1C1=[C][C]=C2C(=[C]1)O[C]O2)O[C])O)O[C])O[C]	-7.8
**5465885**	O1C2=[C][C]=[C][C]=C2[C]([C][C]1C1=[C][C]=[C][C]=[C]1)[N]N	-7.8
**5386297**	o1c(c(c(=O)c2C(=C(C(=[C]c12)O)O[C])O)C1=[C]C(=C([C]=C1O[C])O[C])O[C])C(=O)O	-7.8
**5383438**	o1c2=[C]C(=[C]C(=c2c(=O)c(c1C1=[C]C(=C([C]=[C]1)O[C])O[C])O[C])O)O	-7.8
**5381920**	O1C2=C([C]=[C]C(=[C]2)O)C(=O)[C]=C1C1=[C]C(=C(C(=[C]1)O[C])O[C])O[C]	-7.8
**5380905**	o1c2=[C]C(=[C]C(=c2c(=O)c(c1C1=[C]C(=C([C]=[C]1)O[C])O)O[C])O)O	-7.8
**5379265**	O1C2=C(C(=[C]C(=[C]2)O)O)C(=O)[C]=C1C1=[C]C(=C(C(=[C]1)O[C])O[C])O[C]	-7.8
**5378210**	O1C(=[C]C(=O)C2=C1[C]=C([C]=C2O)O)C1=[C][C]=[C][C]=C1O[C]	-7.8
**5377945**	o1c(c(c(=O)c2=C(C(=C([C]=c12)O)O[C])O)O)C1=[C][C]=C([C]=[C]1)O	-7.8
**5376891**	O1[C]=C(C(=O)C2=C1[C]=C([C]=[C]2)O)C1=[C][C]=[C][C]=[C]1	-7.8
**5353911**	O1[C]=C(C(=O)C2=C([C]=C(C(=C12)O[C])O)O)C1=[C][C]=C([C]=[C]1)O	-7.8
**5352032**	o1c(c(c(=O)c2=C(C(=C([C]=c12)O)O[C])O)O[C])C1=[C][C]=C([C]=[C]1)O	-7.8
**5352005**	o1c2=[C]C(=[C]C(=c2c(=O)c(c1C1=[C]C(=C([C]=[C]1)O[C])O[C])O[C])O)O[C]	-7.8
**5320945**	o1c(c(c(=O)c2C(=[C]C(=[C]c12)O[C])O)O)C1=[C][C]=C(C(=[C]1)O[C])O	-7.8
**5320181**	O1C(=[C]C(=O)C2=C(C(=C([C]=C12)O)O)O)C1=[C]C(=C([C]=[C]1)O)O[C]	-7.8
**5281695**	o1c(c(c(=O)c2=C(C(=C([C]=c12)O)O[C])O)O[C])C1=[C][C]=C([C]=[C]1)O[C]	-7.8
**5281654**	o1c2[C]=C([C]=C(c2c(=O)c(c1C1=[C][C]=C(C(=[C]1)O[C])O)O)O)O	-7.8
**5280666**	O1C2=[C]C(=[C]C(=C2C(=O)[C]=C1C1=[C][C]=C(C(=[C]1)O[C])O)O)O	-7.8
**5271551**	O1C2=C(C(=[C]C(=[C]2)O[C])O[C])C(=O)[C][C]1C1=[C][C]=C([C]=[C]1)O	-7.8
**3245800**	C1(=[C]C(=O)C2=[C][C]=[C][C]=C2O1)C1=[C][C]=C([C]=[C]1)S[C]	-7.8
**3084508**	O1C(=[C]C(=O)c2c(c(c(c(c12)O[C])O[C])O[C])O)C1=[C]C(=C(C(=[C]1)O)O[C])O	-7.8
**3070246**	c1(c([C])c(=O)c2=[C][C]=C(C(=c2o1)[C]N([C][C])[C][C])O[C])C1=[C][C]=C(Cl)[C]=[C]1	-7.8
**2734290**	O1[C]=C(C(=O)C2=C1[C]=C([C]=C2[C])O[C])C1=[C][C]=[C][C]=[C]1	-7.8
**2017481**	C1(=[C][C]=C2C(=O)C(=[C]OC2=[C]1)C1=[C][C]=[C][C]=[C]1)OS([C])([O])[O]	-7.8

**1241435**	O1C2=C([C]=[C][C]=[C]2)C(=O)[C]=C1C1=[C][C]=C([C]=[C]1)N([C])[C]	-7.8
**736738**	O1C2=[C][C]=[C][C]=C2C(=O)[C]([C]1C1=[C][C]=[C][C]=[C]1)O	-7.8
**632255**	O1C(=[C]C(=O)c2c(c(c(c(c12)O[C])O[C])O)O)C1=[C][C]=C(C(=[C]1)O[C])O[C]	-7.8
**631782**	o1c2[C]=C([C]=[C]c2c(=O)c(c1[C])C1=C([C]=C([C]=[C]1)O[C])O[C])O[C][C]=[C]	-7.8
**369612**	O1C2=C([C]=C([C]=C2[C]([C][C]1C1=[C][C]=C(C(=[C]1)O[C])O[C])O[C])[C][C]=[C])O[C]	-7.8
**343083**	C1(=[C]OC2=C([C]=[C]C(=[C]2)O[C])C1=O)C1=[C]C2=C(O[C]O2)[C]=C1O[C]	-7.8
**321347**	O1C2=[C]C(=[C]C(=C2C(=O)[C][C]1C1=[C]C(=C([C]=[C]1)O)O[C])O)O[C]	-7.8
**246330**	O1C2=[C]C(=[C][C]=C2C(=O)[C]([C]1C1=[C][C]=C(C(=[C]1)O)O)O)O	-7.8
**195603**	O1C2=C(C(=[C]C(=[C]2)O[C])O)C(=O)[C][C]1C1=C([C]=[C][C]=C1O)O	-7.8
**188323**	O1C(=[C]C(=O)C2=C(C(=C([C]=C12)O[C])O[C])O)C1=[C][C]=C([C]=[C]1)O	-7.8
**177000**	o1c2=[C]C(=[C][C]=c2c(=O)c([C])c1C1=[C]C(=C(C(=[C]1)O[C])O[C])O[C])O[C]	-7.8
**160237**	O1C(=[C]C(=O)C2=C1[C]=C(C(=C2O)O[C])O[C])C1=[C][C]=C(C(=[C]1)O)O	-7.8
**158311**	O1C(=[C]C(=O)C2=C1[C]=C(C(=C2O)O[C])O[C])C1=C([C]=C(C(=[C]1)O[C])O)O	-7.8
**153441**	O1C(=[C]C(=O)c2c(c(c(c(c12)O)O[C])O[C])O)C1=[C][C]=[C][C]=[C]1	-7.8
**102674**	O1C2=[C][C]=[C][C]=C2[C]([C][C]1C1=[C][C]=[C][C]=[C]1)[N]N	-7.8
**96539**	O1C(=[C]C(=O)c2c(c(c(c(c12)O[C])O[C])O[C])O)C1=[C][C]=C([C]=[C]1)O[C]	-7.8
**26034**	o1c(c(c(=O)c2=C([C]=C([C]=c12)O[C])O[C])O)C1=[C]C(=C([C]=[C]1)O)O	-7.8
**9064**	O1C2=[C]C(=[C]C(=C2[C][C]([C]1C1=[C][C]=C(C(=[C]1)O)O)O)O)O	-7.8
**471**	O1C2=[C]C(=[C]C(=C2C(=O)[C]([C]1C1=[C]C(=C([C]=[C]1)O)O)O)O)O	-7.8
**139592630**	O1C2=C([C]=[C]C(=[C]2)O)[C]2[C][C]1[C]1[C][C]=[C][C](O[C]O2)[C]1	-7.7
**138454529**	O1C2=[C][C]=[C][C]=C2C(=O)[C]([C@]1(C1=[C][C]=[C][C]=[C]1)OC(O)(O)O)O	-7.7
**129864265**	O1C2=C([C]=[C][C]=C2C(=O)[C][C@@]1(C1=[C][C]=[C][C]=[C]1)O)O[C]	-7.7
**129822386**	Brc1c(c(c(c2C(=O)[C]=C(Oc12)C1=[C][C]=[C][C]=[C]1)OC(=O)[C])OC(=O)[C])OC(=O)[C]	-7.7
**102330217**	O1C2=C([C]=[C]C(=[C]2)O[C])C(=O)[C]([C]1C1=[C][C]=C([C]=[C]1)O[C])O	-7.7

**102321610**	C1(=[C]OC2=[C]C(=[C]C(=C2C1=O)O)O)C1=C(C2=C(O[C]O2)[C]=[C]1)O[C]	-7.7
**102017163**	o1c(c(c(=O)c2=C([C]=C(C(=c12)[C][C]C(=[C])[C])O)O)O)C1=[C][C]=[C][C]=[C]1	-7.7
**101591282**	C1(=C(c2c(C(=[C]1)O[C])c(=O)c(c(o2)C1=[C][C]=C(C(=[C]1)O[C])O[C])O[C])O[C])O[C][C][C][C][C]	-7.7
**101179165**	o1c(c(c(=O)c2=C([C]=C([C]=c12)O[C])O[C][C][C]O)O[C])C1=[C]C(=C([C]=[C]1)O[C])O[C]	-7.7
**90000191**	o1c(c(c(=O)c2c(c(c(c(c12)O[C])O[C])O[C])O)O)C1=C(C(=[C][C]=[C]1)O[C])O[C]	-7.7
**74977227**	O1C2=[C]C(=[C]C(=C2[C][C][C]1C1=[C][C]=C(C(=[C]1)O[C])O[C])O)O	-7.7
**70253991**	o1c(c(c(=O)c2c(c(c(c12)O)O[C])O[C])O)O)C1=[C][C]=[C][C]=[C]1	-7.7
**69030367**	O1C2=[C]C(=[C][C]=C2C(=O)[C]=C1C1=[C]C(=[C]C(=[C]1)O[C])O[C])O	-7.7
**54140898**	o1c(c(c(=O)c2=C(C(=[C][C]=c12)O[C][C])O[C][C])O[C][C])C1=[C][C]=[C][C]=[C]1	-7.7
**44412009**	O1C2=C([C])C(=[C]C(=C2C(=O)[C][C]1C1=[C][C]=[C][C]=[C]1)O[C])O[C]	-7.7
**44266353**	O1C2=[C][C]=[C][C]=C2C(=O)/C(=[C]/C2=[C][C]=N[C]=[C]2)/[C]1C1=[C][C]=[C][C]=[C]1	-7.7
**44259869**	o1c(c(c(=O)c2=C(C(=C([C]=c12)O)O[C])O)O)C1=[C][C]=C(C(=[C]1)O[C])O[C]	-7.7
**44258621**	O1c2c3C(=O)[C]=C(Oc3c(c(c2[C]=[C]C1([C])[C])O[C])O[C])C1=[C]C(=C(C(=[C]1)O[C])O[C])O[C]	-7.7
**35028119**	O1C(=[C]C(=O)c2c(c(c(c(c12)O[C])O[C])O[C])O[C])C1=[C][C]=[C]C(=[C]1)O[C]	-7.7
**25068463**	O1C2=[C]C(=[C]C(=C2[C]C(=O)[C]1C1=[C][C]=C(C(=[C]1)O[C])O[C])O[C])O[C]	-7.7
**18324985**	Ic1c(=O)c2=[C][C]=[C][C]=c2oc1C1=[C][C]=C([C]=[C]1)S(=O)(=O)[C]	-7.7
**15389441**	O1C2=C([C]=C3C(=[C]2)O[C]=C(C3=O)C2=[C]C(=C([C]=C2O[C])O[C])O[C])[C]=[C]C1([C])[C]	-7.7
**14372618**	O1C(=[C]C(=O)C2=C1[C]=C(C(=C2O[C])O)O[C])C1=[C][C]=[C][C]=[C]1	-7.7
**14254494**	O1[C]=C(C(=O)C2=C1[C]=C([C]=C2[C])O)C1=[C][C]=[C][C]=[C]1	-7.7
**13964544**	O1C(=[C]C(=O)C2=C1[C]=C([C]=C2O[C])O)C1=[C][C]=C(C(=[C]1)O[C])O[C]	-7.7
**13916278**	o1c(c(c(=O)c2C(=[C]C(=C(c12)O[C])O)O)O[C])C1=[C][C]=C([C]=[C]1)O[C]	-7.7
**12360936**	O1C2=[C]C(=[C][C]=C2[C]([C][C]1C1=[C][C]=[C][C]=[C]1)O)O[C]	-7.7
**12241083**	O1[C]=C(C(=O)C2=C1[C]=C([C]=C2O)O)C1=[C][C]=C([C]=[C]1)N(=O)=O	-7.7
**12132943**	O1c2c(c(c([C])c(c2C(=O)[C][C]1C1=[C][C]=[C][C]=[C]1)O)O[C])[C]=O	-7.7
**12132870**	O1[C]=C(C(=O)C2=C1[C]=C(C(=[C]2)O)O[C])C1=[C][C]=C([C]=[C]1)O[C]	-7.7

**11726019**	O1C(=[C]C(=O)c2c(c(c(c(c12)O)O[C])O[C])O)C1=[C][C]=C(C(=[C]1)O[C])O	-7.7
**10594862**	Brc1c(=O)c2=[C][C]=[C][C]=c2oc1C1=[C][C]=C([C]=[C]1)[S@@](=O)[C]	-7.7
**5491643**	O1C(=[C]C(=O)C2=C(C(=C([C]=C12)O)O[C])O[C])C1=[C][C]=C(C(=[C]1)O[C])O[C]	-7.7
**5488781**	O1[C]=C(C(=O)C2=C1[C]=C(C(=C2O)O[C])O)C1=[C][C]=C(C(=[C]1)O)O[C]	-7.7
**5386961**	o1c(c(c(=O)c2=C([C]=C(C(=c12)O[C])O)O)O[C])C1=[C]C(=C([C]=[C]1)O)O[C]	-7.7
**5317287**	o1c(c(c(=O)c2=C(C(=C([C]=c12)O[C])O[C])O)O)C1=[C]C(=C([C]=[C]1)O[C])O	-7.7
**5316900**	o1c2=[C]C(=[C]C(=c2c(=O)c(c1C1=[C]C(=C([C]=[C]1)O)O[C])O[C])O)O	-7.7
**5281706**	O1[C]=C(C(=O)C2=C([C]=C([C]=C12)O[C])O)C1=C([C]=C([C]=[C]1)O)O	-7.7
**5281666**	o1c(c(c(=O)c2=C([C]=C([C]=c12)O)O)O)C1=[C][C]=C([C]=[C]1)O[C]	-7.7
**5280373**	O1[C]=C(C(=O)C2=C1[C]=C([C]=C2O)O)C1=[C][C]=C([C]=[C]1)O[C]	-7.7
**4303567**	O1C(=[C]C(=O)C2=C1[C]=C([C]=C2OC(=O)[C])OC(=O)[C])C1=[C][C]=C([C]=[C]1)O[C]	-7.7
**3825918**	o1c2[C]=[C][C]=[C]c2c(=O)c(c1C1=[C][C]=[C][C]=[C]1)N1[C]=NN=N1	-7.7
**1666397**	o1c2=[C][C]=[C][C]=c2c(=O)c(c1C1=[C][C]=[C][C]=[C]1)OC(=O)[C]	-7.7
**634113**	o1c2=[C]C(=[C]C(=c2c(=O)c(c1C1=[C]C(=C(C(=[C]1)O[C])O[C])O[C])O[C])O[C])O[C]	-7.7
**631791**	o1c(c(c(=O)c2c(c(c3c([C]=[C]O3)c12)O[C])O[C])O[C])C1=[C][C]=[C][C]=[C]1	-7.7
**624789**	O1[C]=C(C(=O)C2=C1[C]=C(C(=[C]2)O[C])O[C])C1=[C][C]=[C][C]=[C]1	-7.7
**602425**	O1C2=C(C(=[C][C]=C2C(=O)[C]([C]1C1=[C][C]=C(C(=[C]1)O[C])O[C])O)O[C])O[C]	-7.7
**586387**	O1C2=[C]C(=[C]C(=C2C(=O)[C]([C]1C1=[C][C]=C([C]=[C]1)O[C])O)O)O	-7.7
**462696**	O1C2=[C]C(=[C]C(=C2C(=O)[C][C]1C1=[C]C(=C(C(=[C]1)O[C])O[C])O[C])O)O	-7.7
**442621**	o1c(c(c(=O)c2C(=C(C(=[C]c12)O[C])O)O)O[C])C1=[C][C]=C(C(=[C]1)O)O[C]	-7.7
**343081**	O1[C]=C(C(=O)C2=C1C1=C([C]=[C]2)O[C]O1)C1=[C]C(=C([C]=[C]1)O[C])O[C]	-7.7
**331149**	O1C(=[C]C(=O)c2c(c(c(c(c12)O[C])O[C])O[C])O[C])C1=[C]C2=C(C(=[C]1)O[C])O[C]O2	-7.7
**147806**	O1C2=[C][C]=[C][C]=C2C(=O)[C]([C]1C1=[C][C]=[C][C]=[C]1)O	-7.7
**129394**	O1c2c(c(c([C])c(c2[C]([C][C]1C1=[C][C]=[C][C]=[C]1)O)O[C])O)[C]=O	-7.7
**49676**	o1c(c([C])c(=O)c2=[C][C]=C(C(=c12)[C]N([C][C])[C][C])O[C])C1=[C][C]=[C][C]=[C]1	-7.7
**41451**	C1(=[C][C]=C([C]=[C]1)c1c([C])c(=O)c2=[C][C]=[C][C]=c2o1)O[C][C](O)[C]N[C]([C])[C]	-7.7
**134159420**	o1c2[C]=[C][C]=[C]c2c(=O)c(c1C1=C([C]=C(C(=[C]1)O)O)C(=O)O)C1=[C][C]=[C][C]=[C]1	-7.6

**129887676**	O1C2=C([C]=C(C(=[C]2)O)O[C])C(=O)[C]([C]1C1=[C][C]=[C][C]=[C]1)O	-7.6
**129858707**	O1C2=C(C(=[C]C(=[C]2)O)O)C(=O)[C@]([C]1C1=[C]C(=C([C]=[C]1)O)O)(O)O[C]	-7.6
**129858341**	O1C2=C([C]=[C]C(=C2O[C])O[C])C(=O)C([C@@]1(C1=[C][C]=[C][C]=[C]1)O)(O)O	-7.6
**129844543**	O1[C]=C(C(=O)c2c(c(c(c(c12)O)O)O[C])O)C1=[C]C(=C(C(=[C]1)O)O[C])O[C]	-7.6
**129711220**	O1C2=C([C]=[C][C]C2=O)[C][C][C]1C1=[C][C]=[C][C]=[C]1	-7.6
**129710808**	O1C2=[C][C]=[C][C]=C2C(=O)[C][C@@]1(C1=[C][C]=[C][C]=[C]1)/[C]=[C]/C(=[C])[C]	-7.6
**129680256**	O1C2=[C][C]=[C][C]=C2[C]C([C]1C1=[C][C]=[C][C]=[C]1)(O)O	-7.6
**129661517**	O1C2=[C]C(=[C]C(=C2[C](C(=O)[C]1C1=[C][C]=[C][C]=[C]1)O)O)O	-7.6
**129633578**	o1c(c(c(=O)c2[C]=[C]C(=C(c12)O[C])O[C])O)C1=[C][C]=C(C(=[C]1)O[C])O[C]	-7.6
**101584236**	O1C2=C(C(=[C]C(=C2C(=O)[C][C]1C1=[C][C]=[C][C]=[C]1)O[C])O[C])/[C]=[C]\C([C])([C])O	-7.6
**67743168**	O1C2=[C]C(=[C]C(=C2[C]([C]([C]1C1=[C]C(=[C][C]=[C]1)O)O)O)O)O	-7.6
**67425485**	C1(=[C]C2=C(C(=[C]1)O[C])C(=O)[C]=C(O2)C1=[C][C]=C(C(=[C]1)O[C])O[C])O[C]P(=O)([C]([C])[C])[C]([C])[C]	-7.6
**57340185**	C1(=[C]C(=O)C2=C([C]=C([C]=[C]2)O)O1)C1=[C][C]=C(I)[C]=[C]1	-7.6
**54423602**	c1(c(c(=O)c2=[C][C]=[C][C]=c2o1)C1=[C][C]=[C][C]=[C]1)O[C]([C])[C]	-7.6
**51041990**	o1c2=[C]C(=[C]C(=c2c(=O)c(c1C1=[C]C(=[C]C(=C1[C][C]=C([C])[C])O)O[C])O[C])O)O	-7.6
**44258304**	O1C(=[C]C(=O)C2=C1[C]=C([C]=C2O[C])O[C])C1=[C]C(=C([C]=C1O)O)O	-7.6
**44257206**	o1c([C])c(c(=O)c2=[C][C]=C(C(=c12)C(=O)[C])O)C1=[C][C]=[C][C]=[C]1	-7.6
**44257187**	O1C2=C(C(=[C]C(=C2[C][C]C([C])([C])O)O[C])O[C])[C][C][C]1C1=[C][C]=[C][C]=[C]1	-7.6
**23786424**	O1C2=C(C(=[C]C(=C2O[C])O)O[C])C(=O)[C][C]1C1=[C][C]=[C][C]=[C]1	-7.6
**21270152**	O1[C]=C(C(=O)C2=[C][C]=C(C(=C12)[C])O)C1=[C][C]=C([C]=[C]1)N(=O)=O	-7.6

**16681753**	O1C(=[C]C(=O)C2=C1[C]=C(C(=C2O)O[C])O[C])C1=[C]C(=C(C(=[C]1)O[C])O)O[C]	-7.6
**14732294**	O1C2=[C]C(=[C]C(=C2[C][C]([C]1C1=[C][C]=C(C(=[C]1)O)O)O)O[C])O[C]	-7.6
**14057036**	O1C2=C(C3=C(C(=[C]2)O)C(=O)C(=[C]O3)C2=[C]C(=C([C]=C2O[C])O[C])O[C])[C]=[C]C1([C])[C]	-7.6
**13291608**	o1c(c(c(=O)c2C(=C(C(=[C]c12)O[C])O[C])O)O)C1=[C][C]=[C][C]=[C]1	-7.6
**13045799**	O1C2=[C][C]=[C][C]=C2[C][C]([C]1C1=[C][C]=[C][C]=[C]1)OC(=O)[C]	-7.6
**10665307**	Brc1c(=O)c2[C]=[C][C]=[C]c2oc1C1=[C][C]=C([C]=[C]1)S[C]	-7.6
**10475482**	o1c(c(c(=O)c2c(c(c(c12)O[C])O[C])O[C])O)O)C1=[C]C(=C([C]=[C]1)O)O[C]	-7.6
**9798295**	O1C2=C(C(=C(C(=[C]2)O[C])O[C])O[C])C(=O)[C][C]1C1=[C]C(=C([C]=[C]1)O)O	-7.6
**5491798**	o1c(c(c(=O)c2=C([C]=C(C(=c12)O[C])O[C])O)O)C1=[C][C]=[C][C]=[C]1	-7.6
**5490001**	o1c2=[C]C(=[C][C]=c2c(=O)c(c1C1=[C]C(=C(C(=[C]1)O[C])O[C])O[C])O[C])O	-7.6
**5381919**	O1C(=[C]C(=O)C2=C([C]=C([C])[C]=C12)O)C1=[C]C(=C(C(=[C]1)O[C])O[C])O[C]	-7.6
**5378391**	o1c([C])c(c(=O)c2=[C][C]=C(C(=c12)[C][C]=[C])O)C1=[C][C]=[C][C]=[C]1	-7.6
**5281606**	c1(c(c(=O)c2C(=[C]C(=C(c2o1)O[C])O[C])O)O[C])C1=C(C(=[C][C]=[C]1)Cl)O	-7.6
**5273755**	O1C(=[C]C(=O)C2=C1[C]=C(C(=C2O)O[C])O)C1=[C][C]=C(C(=[C]1)O[C])O[C]	-7.6
**5272653**	O1C(=[C]C(=O)C2=C1[C]=C([C]=C2O)O[C])C1=[C][C]=C(C(=[C]1)O[C])O[C]	-7.6
**3082330**	O1C2=C(C(=[C]C(=[C]2)O)O)C(=O)[C]([C]1C1=[C][C]=[C][C]=C1O)O	-7.6
**2064449**	O1C2=C(C(=[C]C(=[C]2)O[C])O[C])C(=O)[C]([C]1C1=[C]C(=C([C]=[C]1)O[C])O[C])O	-7.6
**636642**	ClC1=C([C]=C2C(=C1O)[C][C]([C](O2)C1=[C][C]=C(C(=[C]1)O)O)O)O	-7.6
**628555**	o1c2=[C]C(=[C][C]=c2c(=O)c(c1C1=[C][C]=C([C]=[C]1)O[C])O[C])O[C]	-7.6
**493376**	O1C2=[C]C(=[C]C(=C2C(=O)[C]=C1C1=[C]C(=C(C(=[C]1)O[C])O[C])O[C])O[C])O[C]	-7.6
**315709**	O1C(=[C]C(=O)C2=C1[C]=C(C(=[C]2)O[C])O[C])C1=[C][C]=C(C(=[C]1)O[C])O[C]	-7.6
**282014**	O1C2=[C]C(=[C]C(=C2[C][C]([C]1C1=[C][C]=C([C]=[C]1)O)O)O)O	-7.6
**189551**	O1C(=C(C(=O)[C@]2([C][C]=[C]C(=C12)O[C])O)O[C])C1=[C][C]=[C][C]=[C]1	-7.6
**182659**	O1C2=[C]C(=[C]C(=C2[C][C]([C]1C1=[C][C]=C(C(=[C]1)O[C])O[C])O)O[C])O[C]	-7.6
**124211**	O1C(=[C]C(=O)c2c(c(c(c(c12)O[C])O[C])O[C])O)C1=C([C]=[C][C]=C1O)O[C]	-7.6
**97332**	o1c(c(c(=O)c2C(=[C]C(=[C]c12)O[C])O[C])O[C])C1=[C]C(=C([C]=[C]1)O[C])O[C]	-7.6
**97142**	o1c(c(c(=O)c2=C([C]=C([C]=c12)O[C])O[C])O)C1=[C]C(=C([C]=[C]1)O[C])O[C]	-7.6
**129881027**	O1[C]=C(C(=O)C2=[C][C]=C(C(=C12)[C]O)O)C1=[C]C(=C(C(=[C]1)[C]O)O)[C]O	-7.5

**129818277**	O1C2=[C]C(=[C][C]=C2[C](C(=O)[C]1C1=[C][C]=[C][C]=[C]1)O[C])O	-7.5
**129661527**	O1C2=[C]C(=[C]C(=C2[C](C(=O)[C@@]1(C1=[C][C]=[C][C]=[C]1)O)O)O)O	-7.5
**102194909**	o1c(c(c(=O)c2c(c(c(c(c12)O[C])O[C][C]=C([C])[C])O[C])O[C])O[C])C1=[C][C]=C(C(=[C]1)O[C])O[C]	-7.5
**91727421**	O1C2=C(C(=[C]C(=[C]2)O[C])O[C])C(=O)[C]([C]1C1=[C][C]=C(C(=[C]1)O[C])O[C])OC(=O)[C]	-7.5
**69047096**	C1(=C([C]=C([C]=[C]1)O[C]O[C])O[C]O[C])[C]1OC2=[C][C]=[C][C]=C2C(=O)[C]1	-7.5
**56658060**	O1C2=[C]C(=[C]C(=C2C(=O)[C]([C]1C1=[C][C]=C(C(=[C]1)O[C])O)O)O)O	-7.5
**54368735**	c1(c(=O)c2[C]=[C][C]=[C]c2oc1C1=[C]C(=C(C(=[C]1)O[C])O[C])O[C])C(=O)N[C][C]Br	-7.5
**44566483**	O1C2=[C]C(=C(C(=C2C(=O)[C][C]1C1=[C][C]=C([C]=[C]1)O[C])O[C])O)O[C]	-7.5
**44260053**	o1c(c(c(=O)c2c(c(c(c(c12)OC(=O)[C][C]([C])[C])O)O[C])O)O[C])C1=[C][C]=C([C]=[C]1)O[C]	-7.5
**44257043**	O1C(=[C][C]=C2[C](C(=C([C]=C12)O)O)O[C])C1=[C][C]=C(C(=[C]1)O)O[C]	-7.5
**44133602**	O1[C]=C(C(=O)C2=C(C(=C([C]=C12)O)O[C])O)C1=[C]C(=C(C(=[C]1)O[C])O)O[C]	-7.5
**42608126**	O1c2c(c(c(c(c2C(=O)[C][C]1C1=[C]C(=C2C(=[C]1)O[C]O2)O[C])O[C])O[C])O[C])O[C]	-7.5
**25058047**	o1c(c(c(=O)c2C(=[C]C(=C(c12)[C][C]=C([C])[C])O[C][C]O)O)O[C][C]O)C1=[C][C]=C([C]=[C]1)O[C]	-7.5
**19358638**	O1C(=[C]C(=O)C2=C1[C]=C([C]=C2O[C])O[C][C]O)C1=[C][C]=C(C(=[C]1)O[C])O[C]	-7.5
**15761521**	o1c2=[C][C]=[C][C]=c2c(=O)c(c1C1=[C][C]=[C][C]=[C]1)[C]([C])C#N	-7.5
**14055876**	O1C(=[C]C(=O)C2=C1[C]=[C]C(=C2O[C])O[C])C1=[C][C]=C(C(=[C]1)O[C])O[C]	-7.5
**11795367**	o1c(c(c(=O)c2c(c(c(c(c12)O[C])O)O[C])O[C])O[C])C1=[C]C2=C([C]=[C]1)O[C]O2	-7.5
**11709692**	o1c(c(c(=O)c2C(=[C]C(=[C]c12)O)O[C])O)C1=[C][C]=C([C]=C1O[C])O	-7.5
**11610052**	O1[C]=C(C(=O)C2=C1[C]=C([C]=C2O)O)C1=C(C(=C([C]=[C]1)O)O)[C][C]=C([C])[C]	-7.5
**11500888**	O1C2=C(C(=[C]C(=[C]2)O)O[C])C(=O)[C]([C]1C1=[C][C]=C([C]=C1O[C])O)O	-7.5
**11471620**	O1[C]=C(C(=O)C2=C1[C]=C([C]=[C]2)O[C])C1=[C][C]=C([C]=C1O)O	-7.5
**9972910**	o1c(c(c(=O)c2=C([C]=C(C(=c12)O[C])O)O)O[C])C1=[C][C]=[C][C]=[C]1	-7.5
**7309334**	O1C2=[C][C]=[C][C]=C2[C][C]([C]1C1=[C][C]=[C][C]=[C]1)O	-7.5
**6453535**	o1c(c(c(=O)c2C(=C(C(=[C]c12)O[C])O[C])O)O)C1=[C][C]=C(C(=[C]1)O[C])O	-7.5
**5459184**	o1c(c(c(=O)c2=C([C]=C(C(=c12)O[C])O[C])O)O[C])C1=[C]C(=C([C]=[C]1)O)O[C]	-7.5
**5407860**	O1[C]=C(C(=O)C2=C1C(=C([C]=[C]2)O)O)C1=[C][C]=[C][C]=[C]1	-7.5

**5380187**	o1c(c(c(=O)c2C(=[C]C(=[C]c12)O)O)C1=[C][C]=C([C]=[C]1)O[C])C(=O)O[C][C]	-7.5
**5379262**	o1c(c(c(=O)c2c(c(c(c12)O[C])O[C])O[C])O)O)C1=[C][C]=[C][C]=[C]1	-7.5
**5377381**	O1[C]=C(C(=O)C2=C1[C]=C([C]=C2O)O)C1=[C][C]=[C][C]=[C]1	-7.5
**5321435**	o1c(c(c(=O)c2C(=C(C(=[C]c12)O)O[C])O)O)C1=[C][C]=C(C(=[C]1)O[C])O	-7.5
**5318041**	O1C(=[C]C(=O)c2c(c(c(c12)O[C])O[C])O[C])O[C])C1=[C][C]=C(C(=[C]1)O)O	-7.5
**5281781**	O1[C]=C(C(=O)C2=C1[C]=C(C(=C2O)O[C])O)C1=[C][C]=C([C]=[C]1)O[C]	-7.5
**5281676**	o1c2=[C]C(=[C]C(=c2c(=O)c(c1C1=[C]C(=C([C]=C1O)O[C])O)O[C])O)O[C]	-7.5
**5280699**	o1c(c(c(=O)c2C(=C(C(=[C]c12)O[C])O[C])O)O[C])C1=[C]C(=C([C]=[C]1)O)O	-7.5
**3083840**	O1[C]=C(C(=O)C2=[C]C(=C([C]=C12)OC(=O)[C])O[C])C1=[C][C]=C([C]=[C]1)O[C]	-7.5
**638006**	O1[C]=C(C(=O)C2=C1[C]=C([C]=[C]2)O[C])C1=[C][C]=[C][C]=[C]1	-7.5
**634724**	o1c(c(c(=O)c2C(=C(C(=[C]c12)O[C][C])O[C])O[C][C])O[C])C1=[C]C(=C([C]=[C]1)O[C][C])O[C]	-7.5
**631176**	O1[C]=C(C(=O)C2=[C]C(=C([C]=C12)O[C])O[C])C1=[C][C]=C(C(=[C]1)O[C])O[C]	-7.5
**631171**	o1c2=[C]C(=[C][C]=c2c(=O)c(c1C1=[C]C(=C([C]=[C]1)O[C])O[C])O[C])O[C]	-7.5
**442583**	O1C(=[C]C(=O)C2=C1[C]=C(C(=C2O[C])O[C])O[C])C1=[C][C]=[C][C]=[C]1	-7.5
**386331**	o1c(c(c(=O)c2=C(C(=C([C]=c12)O[C])O[C])O[C])O[C])C1=[C]C(=C([C]=[C]1)O[C])O[C]	-7.5
**358832**	O1C(=[C]C(=O)c2c(c(c(c12)O[C])O[C])O[C])O)C1=[C][C]=C(C(=[C]1)O[C])O[C]	-7.5
**253959**	O1C2=[C][C]=[C][C]=C2[C]([C][C]1C1=[C][C]=[C][C]=[C]1)O	-7.5
**245874**	O1C2=[C]C(=C(C(=C2C(=O)[C][C]1C1=[C][C]=C([C]=[C]1)O[C])O[C])O)O[C]	-7.5
**181092**	O1C(=[C]C(=O)c2c(c(c(c12)O[C])O[C])O[C])O)C1=[C]C(=C([C]=[C]1)O)O[C]	-7.5
**167616**	O1C2=C(C(=[C]C(=[C]2)O[C])O[C])C(=O)[C][C]1C1=[C]C(=C([C]=[C]1)O[C])O[C]	-7.5
**162464**	O1C(=[C]C(=O)C2=C1[C]=C(C(=C2O)O[C])O[C])C1=[C][C]=C(C(=[C]1)O[C])O	-7.5
**136420**	O1[C]=C(C(=O)C2=C([C]=C([C]=C12)O[C])O[C])C1=[C][C]=C([C]=[C]1)O[C]	-7.5
**97151**	O1C(=[C]C(=O)c2c(c(c(c12)O[C])O[C])O[C])O[C])C1=[C][C]=C2C(=[C]1)O[C]O2	-7.5
**54799**	o1c(c(c(=O)c2c(c(c(c12)O[C])O[C])O[C])O)O[C])C1=[C][C]=C(C(=[C]1)O)O	-7.5
**129887801**	o1c2=[C][C]=C(C(=c2c(=O)c(c1C1=[C]C(=C([C]=[C]1)O[C])O[C])O[C])O)O	-7.4
**129820554**	o1c(c(c(=O)c2=C([C]=C(C(=c12)O[C])O[C])O[C])O)C1=[C]C(=C([C]=[C]1)O[C])O[C]	-7.4

**129733750**	Cl[C]1C2=C([C]=[C][C]=[C]2)O[C]([C]1O)C1=[C][C]=[C][C]=[C]1	-7.4
**129733622**	O1C2=C(C(=C([C]=[C]2)O[C])O)C(=O)C([C@@]1(C1=[C][C]=[C][C]=[C]1)O)(O)O	-7.4
**129674781**	O1C2=C([C]=[C][C]=[C]2)[C][C]([C]1C1=[C][C]=[C][C]=[C]1)O[C]	-7.4
**129035976**	O1C2=C([C]=[C][C]=[C]2)[C][C]([C]1C1=[C][C]=[C][C]=[C]1)O	-7.4
**102180739**	o1c2[C]=[C][C]=[C]c2c(=O)c(c1C1=C(C(=C([C]=[C]1)O[C])O[C])O[C])C1=[C][C]=C([C]=[C]1)O[C]	-7.4
**101561898**	o1c2[C]=[C][C]=[C]c2c(=O)c(c1C1=[C][C]=[C][C]=[C]1)O[C]C#[C]	-7.4
**86641846**	O1C(=[C]C(=O)C2=C([C]=C([C]=C12)O[C]C#[C])O[C])C1=[C]C(=C([C]=[C]1)O[C])O[C]	-7.4
**71428798**	O1[C]=C(C(=O)C2=C1C(=C([C]=[C]2)O)O[C])C1=[C][C]=C([C]=[C]1)O	-7.4
**71346541**	o1c2[C]=[C][C]=[C]c2c(=O)c(c1C1=[C][C]=C([C]=[C]1)N([C])[C])O[C]	-7.4
**67200543**	O1C2=C([C]=[C][C]=[C]2)C(=O)[C]([C]1C1=[C][C]=[C][C]=[C]1)C(=O)OC([C])([C])[C]	-7.4
**66916672**	o1c2=[C][C]=[C][C]=c2c(=O)c(c1C(=O)[C])C1=[C][C]=[C][C]=[C]1	-7.4
**66692593**	O1C2=C([C]=[C][C]=[C]2)[C][C]([C]1C1=[C][C]=[C][C]=[C]1)O	-7.4
**59331779**	O1C(=[C]C(=O)C2=C1[C]=C([C]=C2O[C])O[C]C#N)C1=[C][C]=C(C(=[C]1)O[C])O[C]	-7.4
**54140747**	c1(c(=O)c2=[C][C]=[C][C]=c2oc1C1=[C][C]=[C][C]=[C]1)OS([O])([O])O	-7.4
**23644935**	o1c2=[C]C(=[C][C]=c2c(=O)c(c1N1[C]=N[C]=N1)C1=[C][C]=[C][C]=[C]1)O[C]	-7.4
**23644932**	o1c2=[C]C(=[C][C]=c2c(=O)c(c1N1[C]=N[C]=[C]1)C1=[C][C]=C([C]=[C]1)O[C])O[C]	-7.4
**23644623**	o1c2=[C]C(=[C][C]=c2c(=O)c(c1N1[C]=N[C]=[C]1)C1=[C][C]=[C][C]=[C]1)O[C]	-7.4
**21270160**	O1[C]=C(C(=O)C2=[C][C]=C(C(=C12)[C])O)C1=[C][C]=C([C]=[C]1)[C]	-7.4
**15625549**	O1c2c(c(c(c(c2C(=O)[C][C]1C1=[C]C(=C([C]=[C]1)O[C])O[C])O)O[C])O[C])O[C]	-7.4
**14756305**	O1C2=[C]C(=[C]C(=C2C(=O)[C]([C])[C]1C1=[C][C]=C([C]=[C]1)O)O)O	-7.4
**14630603**	O1[C]=C(C(=O)C2=C(C3=C([C]=C12)O[C]O3)O[C])C1=[C][C]=[C]C(=C1O[C])O[C]	-7.4
**13045744**	O1C2=C(C(=[C]C(=[C]2)O[C])O[C])[C][C][C]1C1=[C]C(=C([C]=[C]1)O[C])O[C]	-7.4
**13045742**	O1C2=C(C(=[C]C(=[C]2)O[C])O[C])[C][C][C]1C1=[C]C(=C([C]=[C]1)O[C])O[C]	-7.4
**11797078**	o1c(c(c(=O)c2c(c(c(c12)O[C])O[C])O[C]O[C])O[C])O)C1=[C]C(=C([C]=[C]1)O)O[C]	-7.4

**10937632**	O1C2=C(C(=C(C(=[C]2)O[C])O[C])O[C])C(=O)[C][C]1C1=[C]C(=C([C]=[C]1)O[C])O[C]	-7.4
**5386963**	o1c(c(c(=O)c2c(c(c(c12)O[C])O)O[C])O)O[C])C1=[C]C(=C(C(=[C]1)O[C])O[C])O[C]	-7.4
**5384810**	o1c(c(c(=O)c2=C([C]=C(C(=c12)OC(=O)[C][C][C])O[C])O)O)C1=[C][C]=C([C]=[C]1)O[C]	-7.4
**5281704**	O1[C]=C(C(=O)C2=C1[C]=C(C(=[C]2)O[C])O)C1=[C][C]=C([C]=[C]1)O[C]	-7.4
**5003667**	O1C2=[C]C(=[C]C(=C2C(=O)[C][C]1C1=[C][C]=C([C]=[C]1)O[C])O[C])O[C]	-7.4
**3084507**	O1C(=[C]C(=O)c2c(c(c(c(c12)O[C])O[C])O[C])O)C1=[C]C(=C(C(=[C]1)O[C])O[C])O	-7.4
**1560918**	C1(=[C]C2=C([C]=[C]1)C(=O)C(=[C]O2)C1=C([C]=[C][C]=[C]1)O[C])OS([C])([O])[O]	-7.4
**633456**	o1c2=[C]C(=[C][C]=c2c(=O)c(c1[C])C1=C([C]=C(C(=[C]1)O[C])O[C])O[C])OC(=O)[C]	-7.4
**631170**	O1C(=[C]C(=O)C2=C1[C]=C([C]=C2O[C])O[C])C1=[C][C]=C(C(=[C]1)O[C])O[C]	-7.4
**631095**	o1c(c(c(=O)c2C(=[C]C(=[C]c12)O[C])O[C])O[C])C1=[C][C]=C([C]=[C]1)O[C]	-7.4
**628412**	o1c(c(c(=O)c2C(=[C]C(=[C]c12)O[C])O[C])O[C])C1=[C][C]=[C][C]=C1O[C]	-7.4
**614295**	[C]1(C(=O)C2=C([C]=[C][C]=[C]2)O[C]1C1=[C][C]=[C][C]=[C]1)S[C][C][C]	-7.4
**462698**	O1c2c(c(c(c(c2C(=O)[C][C]1C1=[C][C]=C([C]=[C]1)O[C])O[C])O[C])O[C])O[C]	-7.4
**462695**	O1C2=C(C(=[C]C(=[C]2)O[C])O[C])C(=O)[C]([C]1C1=[C]C(=C([C]=[C]1)O[C])O[C])O	-7.4
**242486**	O1C2=C(C(=C(C(=[C]2)O[C])O[C])O[C])C(=O)[C][C]1C1=[C][C]=C([C]=[C]1)O[C]	-7.4
**129864121**	o1c2[C]=C([C]=C(c2c(=O)c(c1C1=[C][C]=C([C]=[C]1)O[C](C(=[C])[C])[C]O)O[C])O)O[C]	-7.3
**129834788**	o1c(c(c(=O)c2C(=C(C(=[C]c12)O[C])O[C])O[C])O[C][C])C1=[C][C]=C(C(=[C]1)O[C][C])O[C]	-7.3
**129825943**	O1C2=[C]C(=[C]C(=C2C(=O)[C]=C1C1=[C][C]=[C][C]=C1C(=O)[C][C][C][C][C])O)O[C]	-7.3
**44257042**	O1C(=[C][C]=C2[C](C(=C([C]=C12)O)O)O[C])C1=[C][C]=C(C(=[C]1)O)O	-7.3
**14606539**	o1c(c(c(=O)c2=C(C(=C([C]=c12)O[C])O[C])O[C])O)C1=[C][C]=[C][C]=[C]1	-7.3
**14304977**	o1c2=[C][C]=[C][C]=c2c(=O)c(c1C1=[C]C(=C(C(=[C]1)O[C])O[C])O[C])C(=O)O	-7.3
**12578908**	O1C2=[C]C(=[C]C(=C2C(=O)[C]([C]1)C1=[C][C]=[C][C]=[C]1)O)O	-7.3
**12088189**	o1c2[C]=[C][C]=[C]c2c(=O)c(c1C1=[C][C]=C([C]=[C]1)N([C][C])[C][C])O	-7.3
**11244229**	c1(c(c(=O)c2[C]=[C]C(=[C]c2o1)O[C])C1=[C][C]=[C][C]=[C]1)S[C][C]=[C]	-7.3
**10386481**	O1C(=[C]C(=O)c2c(c(c(c(c12)O[C])O[C])O[C])O)C1=[C]C(=C(C(=[C]1)O[C])O)O[C]	-7.3
**6710704**	O1[C]=C(C(=O)C2=C([C]=C([C]=C12)O[C])O[C])C1=[C][C]=[C][C]=[C]1	-7.3

**5318979**	O1C2=[C][C]=[C][C]=C2[C]([C]([C]1C1=[C][C]=[C][C]=[C]1)O)O	-7.3
**631105**	o1c2[C]=C([C]=C(c2c(=O)c(c1C1=C([C]=C([C]=[C]1)O[C])O[C])O[C])O[C])O[C]	-7.3
**150893**	o1c(c(c(=O)c2c(c(c(c(c12)O[C])O[C])O[C])O[C])O[C])C1=[C]C(=C([C]=[C]1)O[C])O[C]	-7.3
**96118**	O1C(=[C]C(=O)C2=C1[C]=C(C(=C2O[C])O[C])O[C])C1=[C][C]=C([C]=[C]1)O[C]	-7.3
**79730**	O1C(=[C]C(=O)C2=C1[C]=C([C]=C2O[C])O[C])C1=[C][C]=C([C]=[C]1)O[C]	-7.3
**129891972**	O1[C]=C(C(=O)C2=[C][C]=C(C(=C12)O[C])O)C1=[C][C]=C([C]=C1O)O[C]	-7.2
**129762742**	O1C2=C(C(=[C]C(=[C]2)O[C])O)C(=O)C([C]1C1=[C][C]=C([C]=[C]1)O[C])(O)O	-7.2
**129674619**	[C]1(C2=C([C]=[C][C]=[C]2)O[C]([C]1)C1=[C][C]=[C][C]=[C]1)S	-7.2
**124037363**	O1C2=C([C]=[C][C]=[C]2)[C]([C]([C]1C1=[C][C]=[C][C]=[C]1)O)O	-7.2
**91584129**	O1C2=[C]C(=C(C(=C2C(=O)[C]=C1C1=[C][C]=C(C(=C1O[C])O[C])O)O)O[C])O	-7.2
**44257041**	O1C(=[C][C]=C2[C](C(=C([C]=C12)O)O)O[C])C1=[C][C]=C([C]=[C]1)O	-7.2
**21573556**	O1C2=C(C(=[C]C(=[C]2)O[C])O[C])C(=O)[C][C]1C1=[C][C]=C(C(=C1O[C])O[C])O[C]	-7.2
**12405140**	O1C2=[C]C(=C(C(=C2C(=O)[C][C]1C1=[C][C]=C(C(=[C]1)O[C])O[C])O[C])O[C])O[C]	-7.2
**6399103**	O1C2=[C][C]=[C][C]=C2C(=O)[C]([N]O)[C]1C1=[C][C]=[C][C]=[C]1	-7.2
**5487785**	O1[C]=C(C(=O)C2=C(C(=C([C]=C12)O[C])O[C])O)C1=[C][C]=C([C]=[C]1)O	-7.2
**1546800**	O1C2=C([C]=[C][C]=[C]2)C(=O)[C]([C]1C1=[C][C]=[C][C]=[C]1)O	-7.2
**632958**	O1[C]=C(C(=O)C2=[C]C(=C([C]=C12)O[C])O[C])C1=[C]C(=C([C]=C1O[C])O[C])O[C]	-7.2
**632135**	O1C(=[C]C(=O)C2=C([C]=C(C(=C12)O[C])O[C])O[C])C1=[C]C(=C([C]=[C]1)O[C])O[C]	-7.2
**624831**	o1c(c(c(=O)c2=C([C]=C([C]=c12)O[C])O[C])O)C1=[C][C]=C([C]=[C]1)O[C]	-7.2
**462694**	O1C2=[C]C(=[C][C]=C2C(=O)[C]([C]1C1=[C][C]=C([C]=[C]1)O[C])O[C])O	-7.2
**261859**	O1C(=[C]C(=O)c2c(c(c(c(c12)O[C])O[C])O[C])O)C1=[C]C(=C(C(=[C]1)O[C])O[C])O[C]	-7.2
**155365**	O1C2=C(C3=C(C(=[C]2)O[C])C(=O)[C]=C(O3)C2=[C][C]=[C][C]=[C]2)[C]([C]1)[C](C([C])([C])OC(=O)[C])OC(=O)[C]	-7.2
**145659**	O1C(=[C]C(=O)C2=C1[C]=C(C(=C2O[C])O[C])O[C])C1=[C][C]=C(C(=[C]1)O[C])O[C]	-7.2
**117900**	o1c(c(c(=O)c2C(=[C]C(=[C]c12)O[C])O[C])O[C])C1=[C][C]=[C][C]=[C]1	-7.2
**68077**	O1C(=[C]C(=O)c2c(c(c(c(c12)O[C])O[C])O[C])O[C])C1=[C][C]=C([C]=[C]1)O[C]	-7.2

**132507960**	O1[C]=C(C(=O)C2=[C]C(=C([C]=C12)[C]O)O[C])C1=[C][C]=C([C]=[C]1)O	-7.1
**129889097**	O1C2=C(C(=[C][C]=[C]2)[C])[C@]([C])([C@@]([C])([C@]1([C])C1=[C][C]=[C][C]=[C]1)O)O	-7.1
**129882752**	O1[C]=C(C(=O)C2=C([C]=C(C(=C12)O[C])O[C])O[C])C1=[C][C]=C([C]=[C]1)O	-7.1
**129882615**	O1[C]([C]2C(=O)[C]3[C]=[C][C][C]1[C]3C2=O)C1=[C][C]=[C][C]=[C]1	-7.1
**129848680**	o1c(c(c(=O)c2C(=C([C]=C(c12)O[C])O[C])O[C])O[C])C1=[C][C]=C(C(=[C]1)O[C])O[C]	-7.1
**23270524**	O1C2=[C]C(=[C]C(=C2C(=O)[C]([C]1C1=C([C]=C([C]=[C]1)O[C])O[C])OC(=O)[C])O[C])O[C]	-7.1
**15761527**	C1(C(=O)C2=[C][C]=[C][C]=C2O[C]1C1=[C][C]=[C][C]=[C]1)([C]Cl)[C]Cl	-7.1
**14034216**	o1c(c(c(=O)c2=C([C]=C(C(=c12)O[C])O[C])O)O)C1=[C]C(=C([C]=[C]1)O[C])O[C]	-7.1
**10526049**	o1c(c(c(=O)c2c(c(c(c12)O[C])O[C])O[C]O[C])O[C])O)C1=[C][C]=C([C]=[C]1)O[C]	-7.1
**5320351**	o1c(c(c(=O)c2=C(C(=C([C]=c12)O[C])O[C])O)O[C])C1=[C][C]=C(C(=[C]1)O[C])O[C]	-7.1
**72344**	O1C(=[C]C(=O)c2c(c(c(c12)O[C])O[C])O[C])O[C])C1=[C][C]=C(C(=[C]1)O[C])O[C]	-7.1
**139030767**	O1[C]=C(C(=O)C2=[C][C]=C(C(=C12)O[C])C(=O)[C][C]O)C1=[C][C]=C([C]=[C]1)O[C]	-7
**129824418**	O1C2=[C][C]=[C][C]=C2[C][C]([C@]1(C1=[C][C]=[C][C]=[C]1)[C]C(=O)O)O	-7
**129818088**	Br[C@]1(C(=O)C2=[C][C]=[C][C]=C2O[C]1C1=[C][C]=[C][C]=[C]1)N(=O)=O	-7
**66780856**	O1C2=C(C(=[C][C]=[C]2)O)C(=O)C([C@]1(C1=[C][C]=[C][C]=[C]1)O[C])(O)O	-7
**44257040**	O1C(=[C][C]=C2[C](C(=C([C]=C12)O)O)O[C])C1=[C][C]=C([C]=[C]1)O[C]	-7
**24850296**	O1C2=[C]C(=[C]C(=C2C(=O)[C@@]([C@]1(C1=[C][C]=C(C(=[C]1)O)O)O[C])(O)[C]C(=O)O[C])O)O	-7
**631156**	O1[C]=C(C(=O)C2=C1C(=C([C]=[C]2)O[C])O[C])C1=C([C]=C([C]=[C]1)O[C])O[C]	-7
**185670**	O1C(=[C]C(=O)C2=C1[C]=C(C(=C2O[C])O[C])O[C])C1=[C]C(=C(C(=[C]1)O[C])O[C])O[C]	-7
**165203**	O1[C]=C(C(=O)C2=C1[C]=C([C]=[C]2)O[C])C1=[C]C(=C([C]=C1O[C])O[C])O[C]	-7
**101958862**	O1C2=[C][C]=[C][C]=C2C(=O)[C][C@]1(C1=[C][C]=[C][C]=[C]1)C(=O)O	-6.9

**44257169**	O1c2c3[C]=[C]Oc3c(c(c2[C]([C]([C]1C1=[C][C]=[C][C]=[C]1)O[C])O[C])O[C])O[C]	-6.9
**389000**	o1c(c(c(=O)c2c(c(c(c(c12)O[C])O[C])O[C])O[C])O[C])C1=[C]C(=C(C(=[C]1)O[C])O[C])O[C]	-6.9
**132281498**	O1C2=C(C(=C(C(=[C]2)O[C])O[C])O[C])C(=O)C([C@]1(C1=[C][C]=[C][C]=[C]1)O[C])(O[C])O[C]	-6.8
**129876105**	O1C2=[C][C]=[C][C]=C2C(=O)[C][C@]1(C1=[C]NN=[C]1)C1=[C][C]=[C][C]=[C]1	-6.8
**11741814**	o1c(c(c(=O)c2c(c(c(c(c12)O[C])O[C])O[C])O[C])O[C])C1=[C][C]=C([C]=[C]1)O[C]	-6.8
**634440**	o1c(c(c(=O)c2C(=C(C(=[C]c12)O[C])O[C][C])O[C][C])O[C])C1=[C]C(=C([C]=[C]1)O[C])O[C][C]	-6.8
**129713274**	Cl[C@@]1([C]C(=S)C2=[C][C]=[C][C]=C2O1)C1=[C][C]=[C][C]=[C]1	-6.7
**129678968**	[C]1(C2=[C][C]=[C][C]=C2O[C@]([C]1)(C1=[C][C]=[C][C]=[C]1)O[C])SOC(=O)[C]	-6.7
**629965**	O1C(=[C]C(=O)C2=C1[C]=[C]C(=C2O[C])O[C])C1=C([C]=[C][C]=C1O[C])O[C]	-6.7
**129776122**	O1C2=[C]C(=C(C(=C2C(=O)C([C]1C1=[C][C]=[C][C]=[C]1)(O[C])O[C])O)O)O[C]	-6.6
**129674706**	O1C2=C([C]=[C][C]=[C]2)[C][C]([C@]1(C1=[C][C]=[C][C]=[C]1)[C]=O)O	-6.6
**90690372**	O1C2=[C][C]=[C][C]=C2C(=O)C([C@@]1(C1=[C][C]=[C][C]=[C]1)O[C])(O[C])O[C]	-6.3
**238791**	C1(=C([C]=C([C]=C1C1=C(C(=[C]C(=[C]1)C(=O)[C])O[C])O[C])C(=O)[C])O[C])O[C]	-6.3
**16092266**	ClC1=[C]C(=N[C]=N1)N1[C][C]N[C][C]1	-6
